# Femto- to microsecond excited-state dynamics of aggregated and monomeric regiopure zinc and copper phthalocyanines

**DOI:** 10.1039/d6cp02573k

**Published:** 2026-08-25

**Authors:** Eleanor R. Windle, Donald G. MacKay, Daniel Graczyk, Christopher C. Rennie, Partha Malakar, Susan J. Quinn, Robert M. Edkins

**Affiliations:** a School of Chemistry, University College Dublin Dublin 4 D04 V1W8, Ireland susan.quinn@ucd.ie; b Department of Pure and Applied Chemistry, University of Strathclyde Glasgow G1 1XL, UK robert.edkins@strath.ac.uk; c Science and Technology Facilities Council, Rutherford Appleton Laboratory Didcot Oxfordshire OX11 0QX UK

## Abstract

Understanding how aggregation state and metal identity affect excited-state dynamics of phthalocyanines (Pcs) is essential for rationalising their performance as photosensitisers in phototherapeutic applications. Here, we investigate the steady-state and time-resolved photophysics of a matched pair of regiopure Pcs with either diamagnetic Zn(ii) (1) or paramagnetic Cu(ii) (2) in solvents that promote either monomeric (DMSO) or mixed aggregate–monomer (H_2_O and MeCN) speciation. Using time-resolved multiple probe (490–900 nm) transient-absorption spectroscopy continuously spanning femtoseconds to hundreds of microseconds, complemented by fluorescence lifetime and steady-state measurements, we correlate speciation with excited-state behaviour for 1 and 2. ZnPc 1 is fluorescent and long-lived in its monomeric form, exhibiting efficient intersystem crossing to an oxygen-sensitive triplet state with a lifetime of 196 µs. Aggregation leads to rapid non-radiative decay on a tens-of-picoseconds timescale, quenching fluorescence and suppressing triplet formation. In contrast, the CuPc analogue 2 undergoes ultrafast intersystem crossing (<2 ps) and displays room-temperature phosphorescence with a triplet lifetime of ∼20 ns that persists even under aggregating conditions. These results highlight potential switchable photodynamic and photothermal behaviour in structurally related Pcs based on aggregation state and the nature of the metal centre acting as kinetic controls of excited-state pathways.

## Abstract 2

Transient-absorption spectroscopy from femto- to microseconds reveals the differences in excited-state dynamics of a matched pair of phthalocyanines depending on metal (Zn *vs*. Cu) and speciation (monomer *vs*. aggregate).

## Graphical abstract

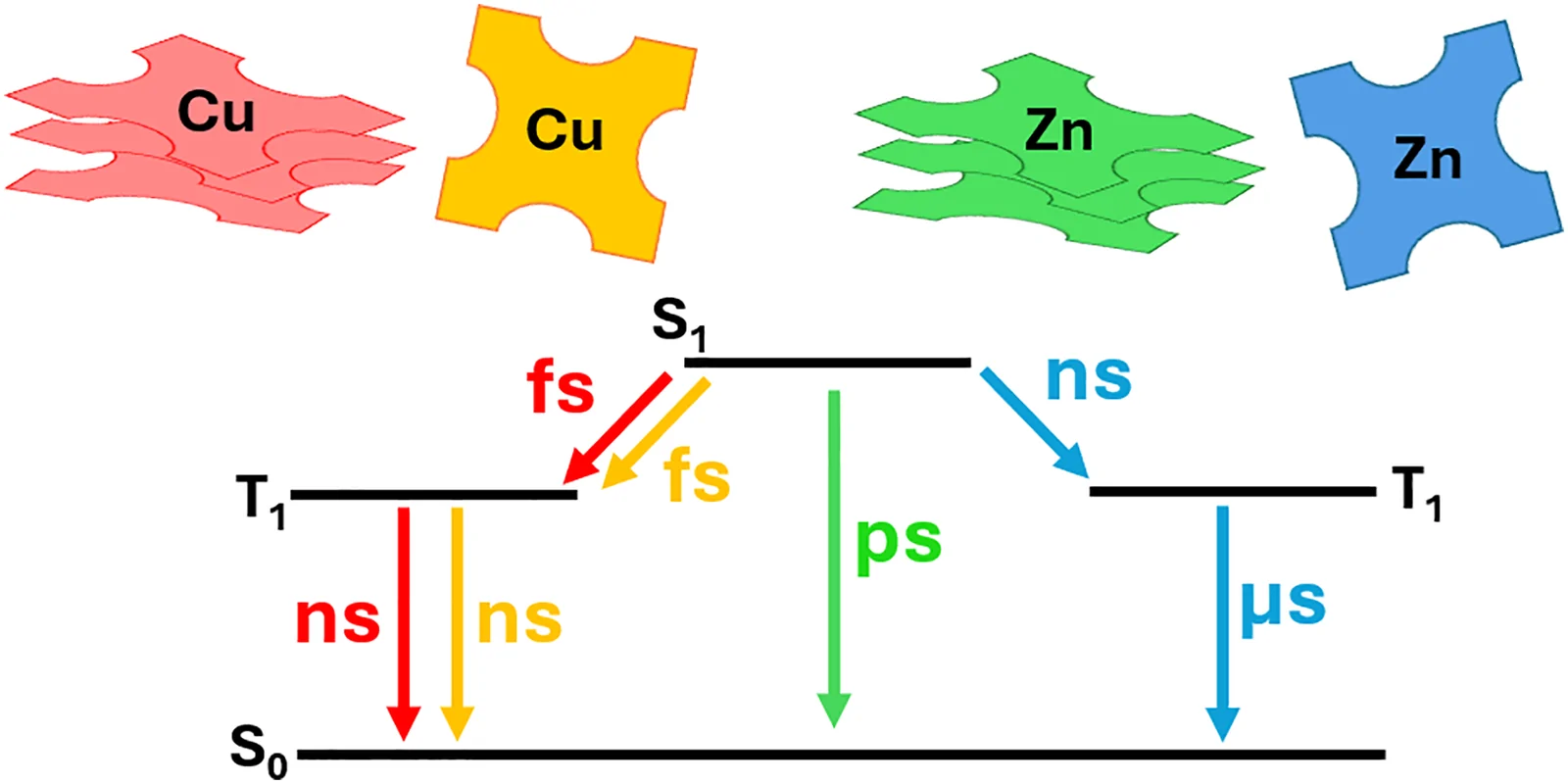

## Introduction

Phthalocyanines (Pcs) are well-established photosensitizers in the cancer phototherapeutic field with the development of new derivatives and applications continuing apace.^1–7^ The photophysical behaviour of Pcs is strongly dictated by their speciation.^8^ For zinc phthalocyanines (ZnPcs), the monomeric form is typically fluorescent and often exhibits a high triplet-state yield (*Φ*_T_ > 0.5, depending on the substituents), enabling efficient singlet oxygen (^1^O_2_) generation for photodynamic therapy (PDT). Upon aggregation, however, ZnPcs undergo pronounced suppression of both fluorescence and triplet formation,^9,10^ restricting their utility for PDT,^11^ with absorbed energy instead being rapidly dissipated as heat that can be harnessed in photothermal therapy (PTT).^12^ The balance between PDT and PTT can also be modulated through structural design.^13^ In some cases, aggregation may also promote alternative deactivation pathways, including charge-transfer or type-I photochemical processes.^14^ Therefore, determining the interplay between speciation and photophysical behaviour is critical for rationalising their phototherapeutic performance, because both the monomeric and aggregated forms may exist and dynamically interchange, influenced by environmental factors such as medium, pH, temperature, and binding to biomolecules, including proteins.^15^ More broadly, Pc aggregation has implications for a range of photonic applications, which has led to theoretical investigations correlating ZnPc speciation and optical properties.^16,17^

Particularly challenging from a spectroscopic viewpoint is the wide range of timescales required to describe the excited-state processes of a new Pc derivative, when the complexed metal is exchanged, or for different aggregation states of the same Pc. Key processes range from ultrafast (fs to ps) dynamics associated with internal conversion (IC), vibrational cooling and intersystem crossing (ISC) to long-lived triplet states with lifetimes of hundreds of µs.^18^ Time-resolved spectroscopy with a wide temporal dynamic range is therefore required to build a complete picture when such varied excited-state dynamics are present. However, it remains challenging to acquire complete excited-state dynamics on a single sample under consistent excitation and sample conditions across all relevant timescales, though recent studies in other areas of photochemistry show the value in doing so.^19,20^ In the case of Pcs, typically, either ultrafast (fs–ns)^21^ transient absorption spectroscopy (TAS) targeting the initial processes following excitation or ns–µs TAS^22^ optimised for the triplet-state dynamics are used in isolation. It is much rarer to study both fs- and ns-TAS on the same phthalocyanine, but where this is done, it has been achieved using two orthogonal experimental setups with non-overlapping timescales and with necessarily different sample preparations.^23^

There have been several investigations of zinc and copper Pcs using ultrafast TA spectroscopy. In 2008, a report compared the literature at the time of the fs–ns dynamics of ZnPc and related complexes showing the lack of consistency within the reports.^21^ They concluded that the conventional energy-flow model of three competing processes (fluorescence, IC and ISC) from the initially formed singlet excited state does not explain the entire kinetics and faster processes must be present. Subsequently a number of studies have used TAS to study the early decays of related monomeric phthalocyanines in different solvents including DMSO and THF.^24,25^ These ultrafast spectroscopic studies only described the kinetics out to a few nanoseconds; however, often the ground state population has not fully recovered in this time due to a long-lived triplet excited state being accessed. The longer-lived states of monomeric ZnPc have been studied in toluene with 1% pyridine^26^ and as well as in DMSO, while for other substituted ZnPc derivatives, the triplet lifetimes were measured in, for example, ethanol and toluene with lifetimes ranging from 220–504 µs,^27^ revealing an oxygen-sensitive µs-scale lifetime of the triplet.

The dynamics of aggregated ZnPcs are remarkably different to their monomeric counterparts, often with significant aggregation-caused quenching observed and a much-reduced triplet yield.^8^ Indeed many researchers have tried to avoid aggregation through various design strategies,^28–30^ but aggregation is often unavoidable. Their fast and speciation-dependent kinetics have hindered studies on aggregated ZnPcs, but they have been studied in hexafluoroisopropanol (HFIP)^24^ and water as well as in CHCl_3_,^31^ revealing rapid relaxation under 100 ps. Addition of disaggregation agents such as poly(ethylene oxide) can restore the monomer and the long-lived triplet state.^32^ Studies of the excited-state dynamics of the same ZnPc in different aggregation states measured under comparable conditions have been limited by their very different timescale regimes.

In contrast to their luminescent Zn(ii) analogues, Cu(ii) phthalocyanines have very different excited-state dynamics due to their open-shell d^9^ configuration and are comparatively less well studied, particularly in the context of phototherapy. One study examined the dynamics using fs pump–probe spectroscopy of a tetrasulfonated copper phthalocyanine in water over 100 ps and observed complete decay in this time period, underscoring the rapid deactivation pathways characteristic of CuPcs.^33,34^

**Fig. 1:**
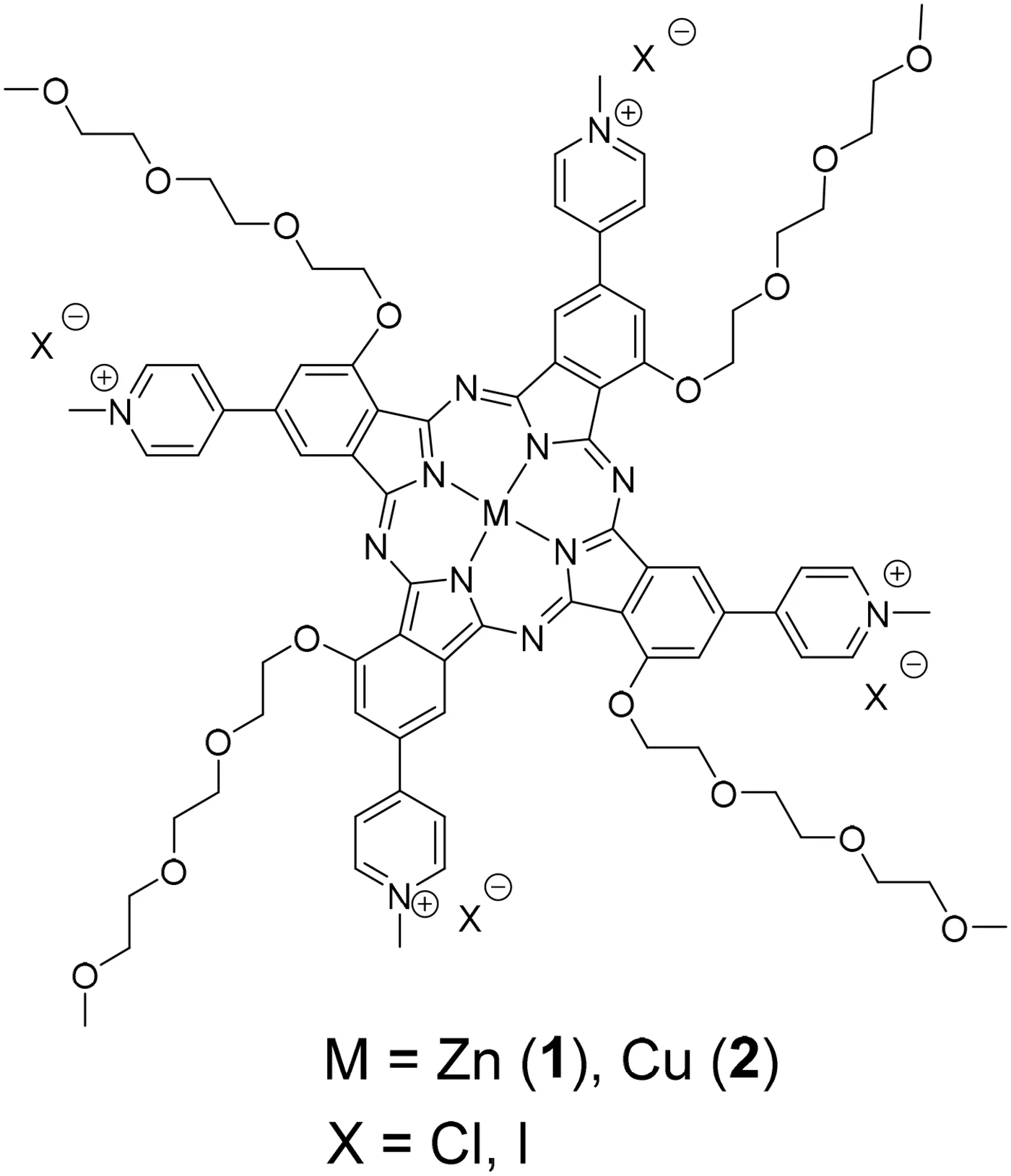
Molecular structure of 1 and 2 as either chloride or iodide salts.

In the present study, we combine steady-state absorption, time-resolved fluorescence, and time-resolved multiple-probe transient-absorption^35–37^ spectroscopies to study the speciation and excited-state behaviour of 1 and 2, a matched pair of regiopure *C*_4h_ phthalocyanines modified with four triethylene glycol methyl ether α-substituents and four *N*-methyl-4-pyridinium β-substituents (Fig. 1).^38^ This allowed us to investigate the solvent-dependent excited-state dynamics of 1 and 2 in both monomeric and aggregated forms over a continuous time range from hundreds of femtoseconds to hundreds of microseconds. We are currently investigating these compounds for their application in photodynamic and photothermal therapy and have recently reported the DNA-binding interactions of 1 with quadruplex-forming sequences.^38^ In this context, understanding their excited-state dynamics and speciation in detail will be important in rationalising their performance in future phototherapeutic application.

## Results

The room-temperature (RT) steady-state absorption and luminescence properties of 1 (X = I, Fig. 1) have been reported by us previously.^38^ Briefly, the absorption spectrum of 1 at 2.3 µM in DMSO solution has a broad Soret band absorbance below 500 nm and a sharp Q band at 737 nm (*ε* = 1.74 × 10^5^ M^−1^ cm^−1^) with a secondary Q_01_ peak at 662 nm with maximum emission at 755 nm (Fig. S1a and b). Upon counter ion exchange to chloride (X = Cl) the absorption and emission are unchanged (Fig. S1c and d). The sharp Q band and fluorescence are characteristic of a monomeric complex. In H_2_O at 2.3 µM, several spectral changes are observed for 1 (X = I) with a decrease in the Soret band absorbance at 416 nm and a decrease and blue-shift in the Q-band absorption maximum to 730 nm (Fig. S1a). An additional band is observed at 687 nm with similar absorbance to the band at 730 nm, which is characteristic of H-type aggregation.^39^ Spectral broadening is also observed for the aggregated form on the red-edge, tailing beyond 850 nm, which is much deeper into the NIR than the monomer Q band. These aggregate absorption features either side of the monomeric Q band may indicate the structure of these aggregates involves face-to-face π-stacking with twisting around the intermolecular Zn–Zn axis.^11^

Weak fluorescence is still observed for a sample of 1 (X = I) in H_2_O at 746 nm with a comparable spectral profile to that of the monomer in DMSO but with greatly reduced intensity (Fig. S1a and b). The excitation profile of 1 is also very similar in DMSO and H_2_O, but it is unlike the UV-vis absorption spectrum in H_2_O, which indicates the fluorescence observed in H_2_O is due to a small population of emissive monomers while the dominant aggregates are non-emissive (Fig. S1b). As was observed in DMSO, the absorption and emission spectral profiles are not significantly affected upon counterion exchange to chloride (X = Cl) (Fig. S1e and f). In DMSO/H_2_O mixtures, there are varying populations of monomers and soluble aggregates depending on the percentage of each solvent (Fig. S2), which for parent ZnPc has previously been related not only to better solvation in DMSO but also solvent microstructure as a function of DMSO/H_2_O ratio.^40^ For 1, as the H_2_O percentage increases, the absorbance decreases at 737 nm and increases at 687 nm and there is a decrease in emission intensity, indicating increasing aggregation. A fully monomeric absorption profile is observed at 80% v/v of DMSO with H_2_O (∼50 mol% DMSO).

**Fig. 2:**
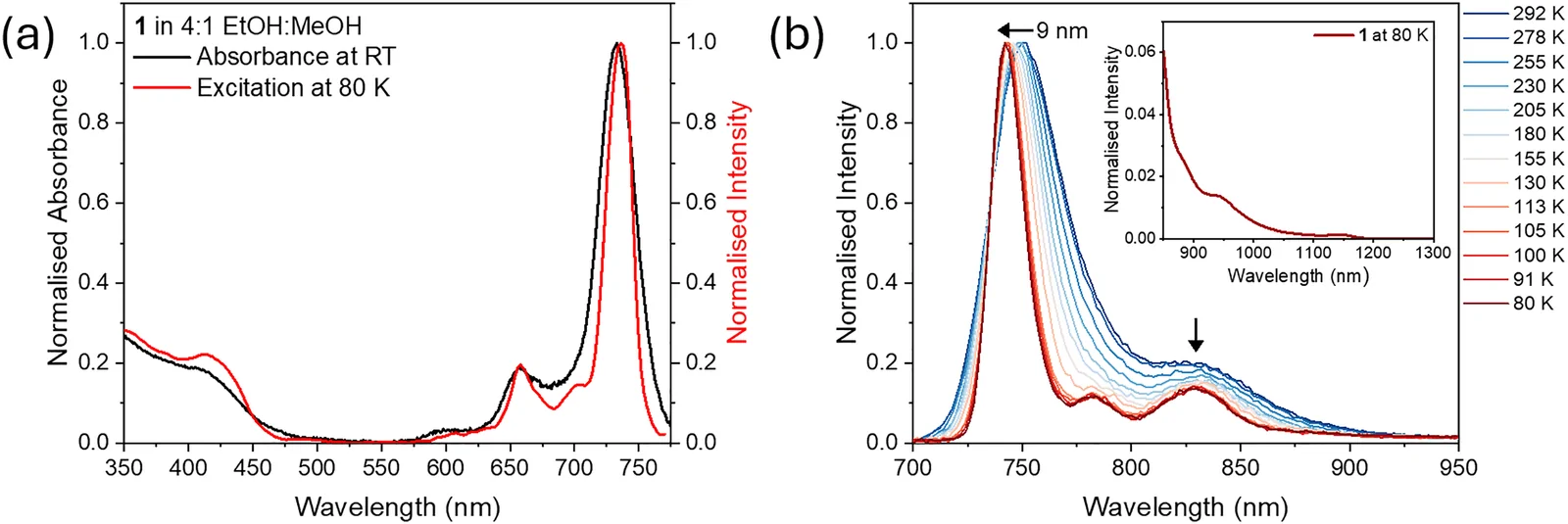
Compound 1 (1 µM) in 4 : 1 EtOH : MeOH mixture. (a) Normalised UV-visible absorption spectrum at RT and excitation spectrum at 80 K (*λ*_em_ = 782 nm). (b) Normalised emission spectra with arrows indicating spectral changes with decreasing temperature from 292 to 80 K (*λ*_ex_ = 400 nm). Inset shows emission spectrum recorded above 850 nm at 80 K.

The emission spectrum of 1 (X = Cl) as a function of temperature was investigated in a 4 : 1 EtOH : MeOH solvent mix that forms an optically clear glass at *ca.* 100 K.^41^ At RT, 1 is monomeric in this solvent mixture, as determined by UV-visible absorption spectroscopy (Fig. 2a). At 292 K, excitation of 1 in this solvent mix at 400 nm results in a similar profile but blue-shifted emission spectrum to that recorded in DMSO with maximum intensity at 751 nm and a shoulder at 830 nm, see Fig. 2b. As the temperature is decreased to 80 K, the maximum further blue shifts from 751 nm to 742 nm (*δ* = 161 cm^−1^) and sharpens significantly with a FWHM of 42 nm (740 cm^−1^) at 292 K but only 16 nm (320 cm^−1^) at 80 K, also providing greater resolution of the additional bands at 782 nm and 830 nm. The tail of the fluorescence spectrum was observed at 80 K to extend to 1200 nm, see inset of Fig. 2b, but there was no detectable phosphorescence from 1, *i.e.* emission was not observed from the triplet state relevant to PDT. The excitation spectrum at 80 K had a similar profile to the RT absorption profile but with a narrower Q_00_ band showing well-resolved vibrational bands at 658 nm and 702 nm, see Fig. 2a.

The fluorescence lifetime of 1 (X = Cl) at RT was measured by time-correlated single-photon counting (TCSPC) in different solvents (Table 1). The lifetimes in DMSO and DMSO-d_6_ were very similar (1.93 *vs.* 1.97 ns), indicating this particular solvent deuteration does not have a large impact on the non-radiative decay rate of 1. However, deuteration of water led to a 37% increase in the fluorescence lifetime (1.81 ns in H_2_O and 2.48 ns in D_2_O), despite the absorption profile indicating a similar aggregate population (Fig. S3). D_2_O extends the lifetime due to the decreased energy of the O–D *vs.* O–H oscillator.^42^ Lifetimes of 1 in 50 : 50 mixtures of D_2_O with DMSO and in D_2_O with 3% pyridine-d_5_, which coordinates to the Zn centre and makes the Pc fully monomeric in water, are also extended compared to those in H_2_O or DMSO.

**Table 1:** Fluorescence lifetimes of 1 (2.3 µM) in various solvents (*λ*_ex_ = 450 nm)

Solvent	*τ* (ns)	*χ* ^2^
DMSO	1.93	1.25
DMSO-d_6_	1.97	1.18
H_2_O	1.81	1.26
D_2_O	2.48	1.27
50 : 50 (v/v) DMSO : D_2_O	2.39	1.13
H_2_O + 3% (v/v) pyridine	1.95	1.08
D_2_O + 3% (v/v) pyridine-d_5_	2.56	1.17
MeCN	1.61	1.17

**Fig. 3:**
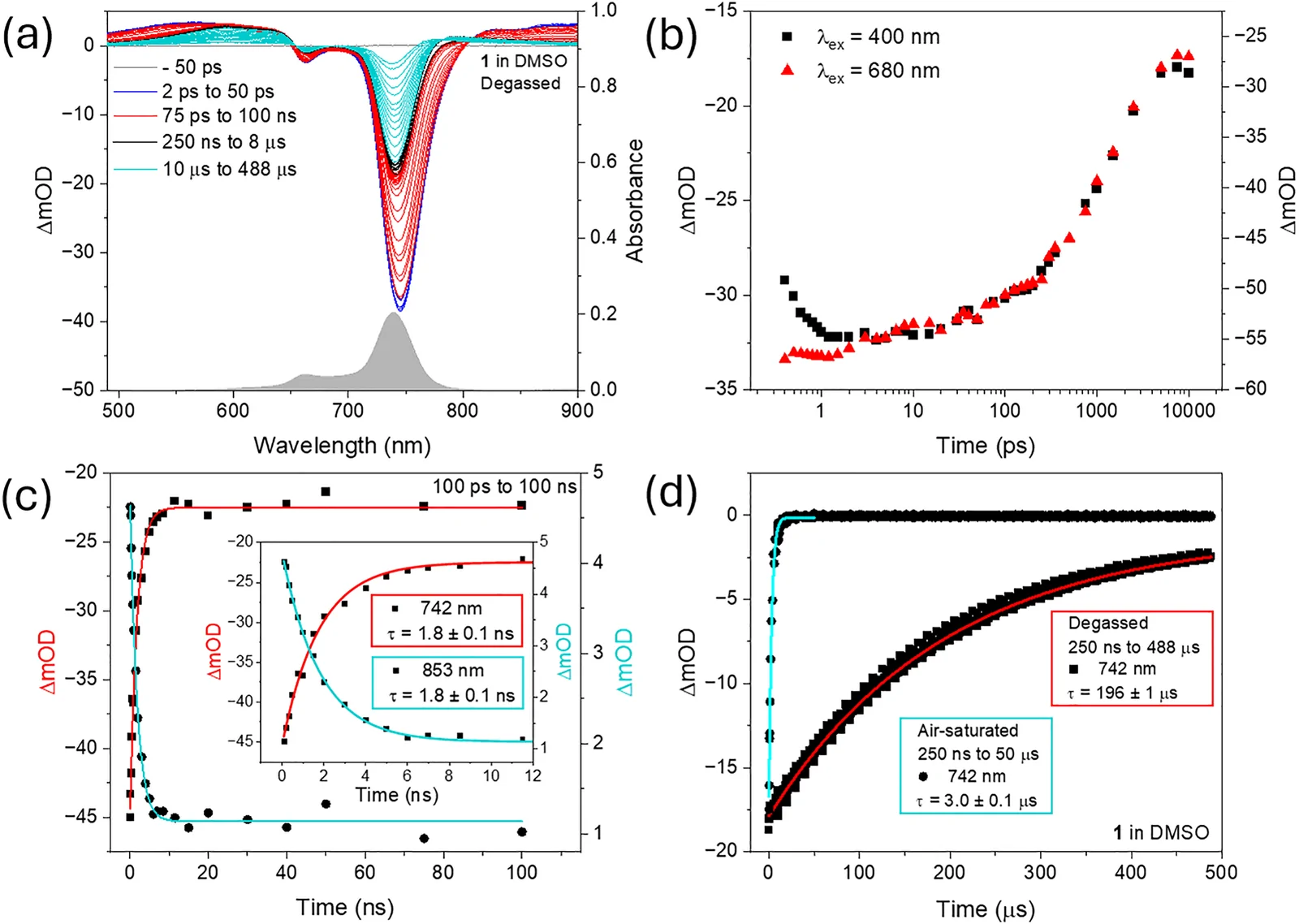
TA spectra and kinetics of 1 (12 µM) in DMSO. (a) TA spectra between 2 ps and 488 µs following bubbling N_2_ for 5 min (left axis). The steady-state absorption spectrum at the same concentration is shown in grey (right axis). (b) Comparison of GSB intensity with two excitation wavelengths (400 and 680 nm) between 400 fs and 10 ns. (c) Recovery of GSB at 742 nm and ESA at 853 nm between 100 ps and 100 ns in DMSO following bubbling with N_2_ for 5 min. (d) Recovery of GSB at 742 nm from 250 ns in DMSO following bubbling with compressed air or N_2_ for 5 min. The data for the air-saturated sample is fit to 50 µs as the GSB is fully recovered by 15 µs (Fig. S7); however, the full data to 488 µs is shown for clarity.

The transient-absorption (TA) spectra of 1 (X = Cl) in DMSO excited at the Soret band (S_2_) at 400 nm are shown in Fig. 3a. The ground-state bleach (GSB) and transient bands grow in over the first 1 ps, likely as S_2_ → S_1_ internal conversion occurs, as this is absent when excited into the Q band with *λ*_ex_ = 680 nm (Fig. 3b). At 2 ps the GSB has a similar profile to the ground state Q band but with maximum at 742 nm. Both GSB and excited-state absorption (ESA) bands show only a small amount of recovery in the initial 100 ps, which is likely due to vibrational relaxation (VR) to the lowest energy level in S_1_, see Fig. S4. This is the case independent of whether excitation is to S_2_ or to S_1_ (*λ*_ex_ = 400 nm or 680 nm). These dynamics were not observed for unsubstituted zinc phthalocyanine^21^ and are reproducible. This is followed by a blue shift to 738 nm by 6 ns, at which point the GSB position matches the maximum ground-state absorption (Fig. S5a). This shift is likely due to the loss of stimulated emission. The transient bands undergo a change in profile over the initial 6 ns (Fig. S5b), where there is a large decrease in the broad transient band above 800 nm (−71% at 850 nm). Meanwhile, the broad transient below 650 nm undergoes a 53% decrease in intensity at 534 nm and a 29% increase at 608 nm. These changes correspond to depletion of S_1_ to (i) the ground state through internal conversion and radiative decay and (ii) T_1_ through ISC. No photobleaching of the sample was observed, as indicated by comparison of pre- and post-TAS experiment UV-visible absorption measurements (Fig. S6).

The recovery of the bleach and transient bands of 1 in DMSO were monitored between 100 ps and 488 µs. Between 100 ps and 100 ns, there is a decay of the bleach band at 742 nm and transient band at 853 nm with a lifetime of 1.8 ns (Fig. 3c). This initial lifetime matches the recorded fluorescence lifetime of 1 (1.93 ns) and is not impacted by the presence of oxygen (Fig. S7) indicating this is the lifetime of the S_1_ state. Monitoring the decay between 250 ns and 488 µs, there is a longer decay of the long-lived transient band at 608 nm and the same bleach band with lifetime of 196 µs under N_2_ purged conditions (Fig. 3d and Fig. S8). This is a very long-lived state that can be assigned as the triplet. At 10 ns, *ca.* 57% of the Q band bleach signal remains, giving an indication of the triplet yield. Upon air-saturation the lifetime of this state is reduced to 3 µs (Fig. 3d), consistent with efficient quenching by O_2_ and in line with the high singlet oxygen quantum yield (*Φ*_Δ_ = 0.56 in DMSO, measured *via* the relative method by comparing reaction rate of singlet-oxygen scavenger 1,3-diphenylisobenzofuran (DPBF) with photosensitized ^1^O_2_ using 1*versus* unsubstituted ZnPc under identical excitation conditions). The long-lived transient bands with maxima at 608 and 784 nm are then likely to be ESA from the triplet state and the change in profile described above is due to the change in population from the S_1_ to T_1_ excited states. Analysis of the photophysical quantum yields (*Φ*_f_ = 0.12, *Φ*_T_ ≈ 0.57) and fluorescence lifetime (*τ* = 1.93 ns) shows that ISC is the dominant S_1_ decay pathway in DMSO, with *k*_ISC_ ≈ 3 × 10^8^ s^−1^, 4.8 times larger than *k*_r_.

**Fig. 4:**
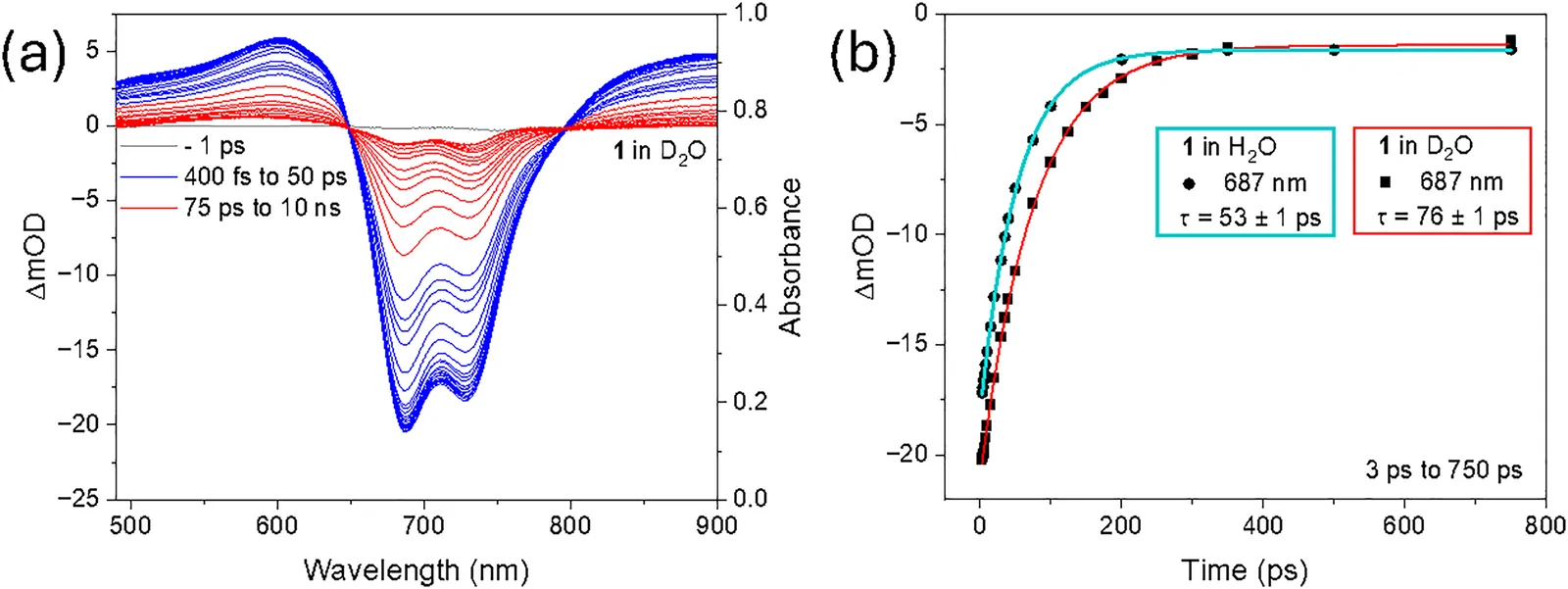
TA spectra and kinetics of 1 in D_2_O (23 µM) (*λ*_ex_ = 400 nm, pump power = 200 nJ). (a) TA spectra in D_2_O between 400 fs to 10 ns (left axis). The steady-state absorption spectrum at the same concentration is shown in grey (right axis). (b) Recovery of GSB at 687 nm in D_2_O and H_2_O (35 µM, *λ*_ex_ = 400 nm, pump power = 120 nJ) between 3 ps and 750 ps. Solid lines show monoexponential fits.

**Fig. 5:**
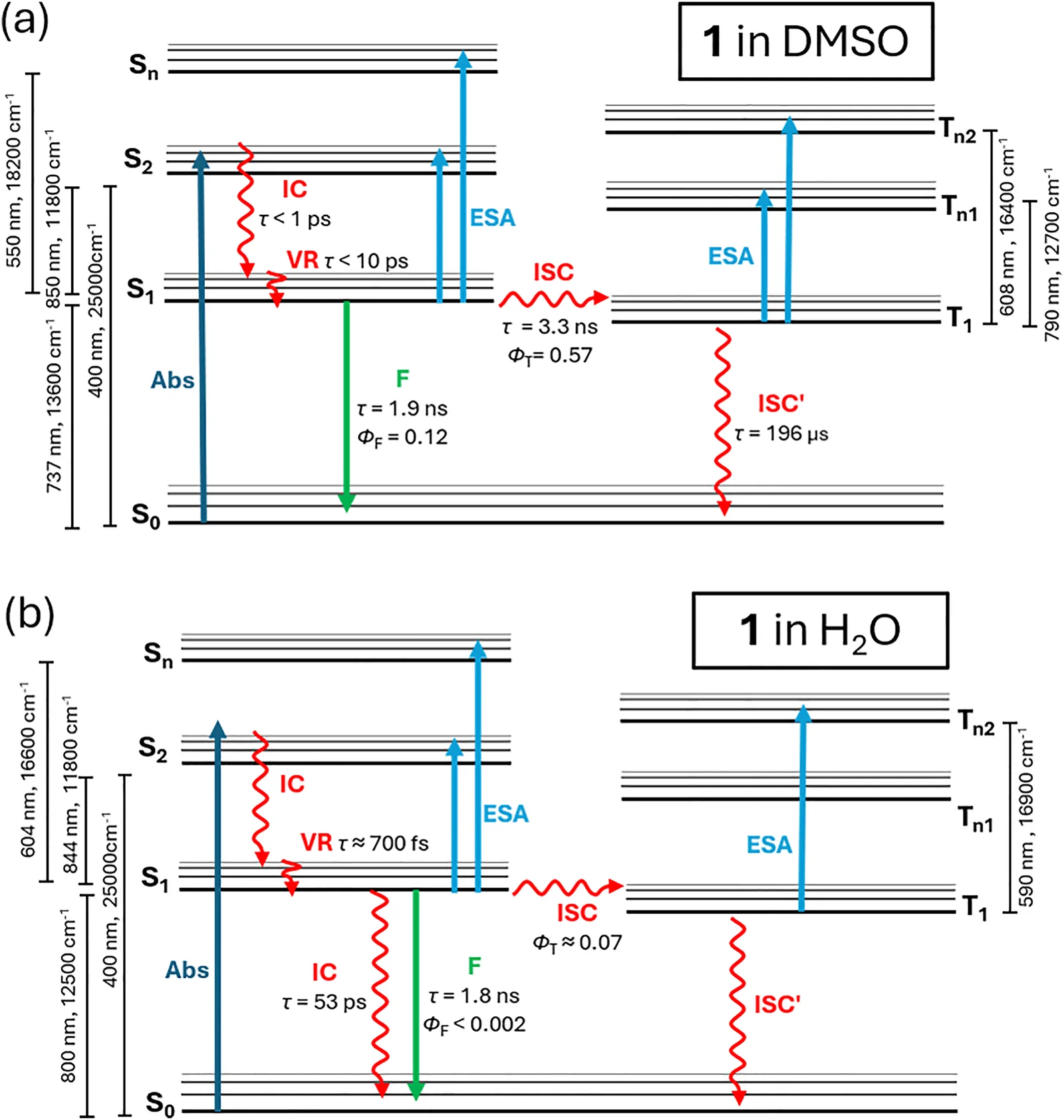
Simplified Jablonski Diagram of 1 in (a) DMSO and (b) in H_2_O. Dark blue arrow shows absorption at pump wavelength. Light blue arrows show absorption of the excited states. Red wavy arrows show non-radiative processes. Green arrow shows radiative relaxation. Approximate energy differences between states, time constants, and quantum yields shown.

The TA spectrum of 1 in D_2_O at 400 fs following excitation has a bleach band with two peaks of similar intensity at 687 and 728 nm corresponding to the ground state absorption of aggregated 1 (Fig. 4a). Two broad transient bands are present at 650 and 850 nm. In contrast to what is observed in DMSO, the majority of the bleach and transient band intensities recover over 750 ps with lifetime of 76 ps (Fig. 4b and Fig. S9a and b). Spectral profile changes are observed in the transient bands over this 750 ps with full recovery of the transient band at 850 nm while significant intensity at 585 nm remains (Fig. S9c). The initial recovery (*τ* = 76 ps) is due to non-radiative decay from S_1_ to S_0_. A persistent signal is observed at 750 ps that recovers on a ns to µs range (Fig. S9d) that is then likely due to access to T_1_. If only monomers of 1 were still in an excited state at this point, the GSB would be expected to have a monomeric profile, similar to that in DMSO; however, there is still significant intensity at 685 nm (Fig. S9c), which indicates aggregates may also access T_1_ The profile and recovery were not found to change significantly when excited at 680 nm (Fig. S10), so the relaxation from S_2_ to S_1_ likely occurs faster than the time resolution of our experiment (∼200 fs). The TA spectrum of 1 in H_2_O has a similar profile to that in D_2_O (Fig. S11); however, the GSB and ESA bands recover 30% faster in H_2_O than in D_2_O (Fig. 4b and Table 2) due to the more efficient quenching by O–H oscillators.^42^ The transient absorption results for 1 in DMSO and H_2_O have been summarised in the form of Jablonski diagrams for comparison in Fig. 5.

**Table 2:** Lifetimes of 1 in aqeuous solution as measured by TAS

Solvent	*λ*_ex_ (nm)	Conc. (µM)	Power (nJ)	GSB *τ* (ps)	Residual GSB band integral at 10 ns
D_2_O	400	46	120	81 (± 1)^a^	6.4%
D_2_O	400	23	200	76 (± 1)^a^	6.6%
D_2_O	680	23	200	74 (± 1)^b^	N/A^c^
H_2_O	400	35	120	53 (± 1)^a^	6.7%

a At 687 nm.

b At 728 nm.

c Overlapping pump precludes calculation.

When pyridine is added to an aqueous solution of 1, a monomeric Pc is produced (*vide supra*). The triplet lifetimes of 1·pyridine in H_2_O under N_2_- and air-saturation are 40 and 2.1 µs, respectively, suggesting the triplet lifetime of the pyridine-bound monomeric form of 1 in water is also sensitive to oxygen concentration and remains a singlet-oxygen photosensitizer (Fig. S12). However, the triplet lifetime of monomeric 1·pyridine is clearly reduced compared to the monomer of 1 in DMSO.

Considering that a large isotope effect was observed for D_2_O *versus* H_2_O, the effect of isotopic substitution on the kinetics of excited monomeric 1 was further investigated by using 1-d_12_ (wherein the four methyl groups of the 4-methylpyridinoium substituents were deuterated) in DMSO and 1 in DMSO-d_6_. The fitted transient-absorption kinetic data are shown in Table 3. It was found that both the singlet and triplet state lifetimes were reduced by approximately 10% in both cases, though the latter may be affected by oxygen concentration. The triplet yield was similar in all cases, and so it can be concluded that these deuterations lead to a relatively minor effect. Furthermore, changing from X = Cl to X = I had little effect on the kinetics in DMSO.

**Table 3:** Lifetimes of 1 in DMSO solution as measured by TAS (*λ*_ex_ = 400 nm)

	Conc. (µM)	Power (nJ)	*τ* @ 742 nm	*τ* @ 608 nm	Residual GSB band integral at 8 µs
1 DMSO	23	200	1.7 (±0.1) ns	194 (±1) µs	43%
			196 (±1) µs		
1-d_12_ DMSO	23	200	1.4 (± 0.1) ns	180 (±2) µs	44%
			184 (±1) µs		
1 DMSO-d_6_	23	200	1.5 (±0.1) ns	173 (±2) µs	44%
			175 (±1) µs		

A solution of 1 in a 50 : 50 by volume mixture of DMSO and D_2_O was studied by TA in which there is a mixture of aggregates and monomers of 1. Fig. S13a and b show the ground state and TA profile of 1 in this mixture with a strong Q band bleach at 738 nm and an aggregate band shoulder at 694 nm. The kinetics are shown in Fig. S13c and d, with the Q band across the full 5 ps and 488 µs time range fitting to a biexponential with lifetimes 140 ps and 2.6 ns at short times and a second bi-exponential function with 24 and 124 µs time constants at longer times. These kinetics can be interpreted as singlet-state non-radiative decay, fluorescence (*cf.* 2.39 ns measured by TCSPC), and triplet lifetimes respectively in this mixed speciation sample. Using this interpretation, the non-radiative process is slower in this solvent mixture than in 100% D_2_O, and singlet lifetime is longer than in D_2_O or DMSO, but the triplet lifetime is shorter than in 100% DMSO, though it is possible that different oxygen concentrations could affect the triplet lifetime. Spectral changes are observed with more rapid recovery of the aggregate band, and a fully monomeric profile is observed at 6 µs confirming only monomers remain in an excited state at this time point (Fig. S14). Additional details of the kinetic fittings and a summary table are provided in Fig. S23–S29 and Table S1.

**Fig. 6:**
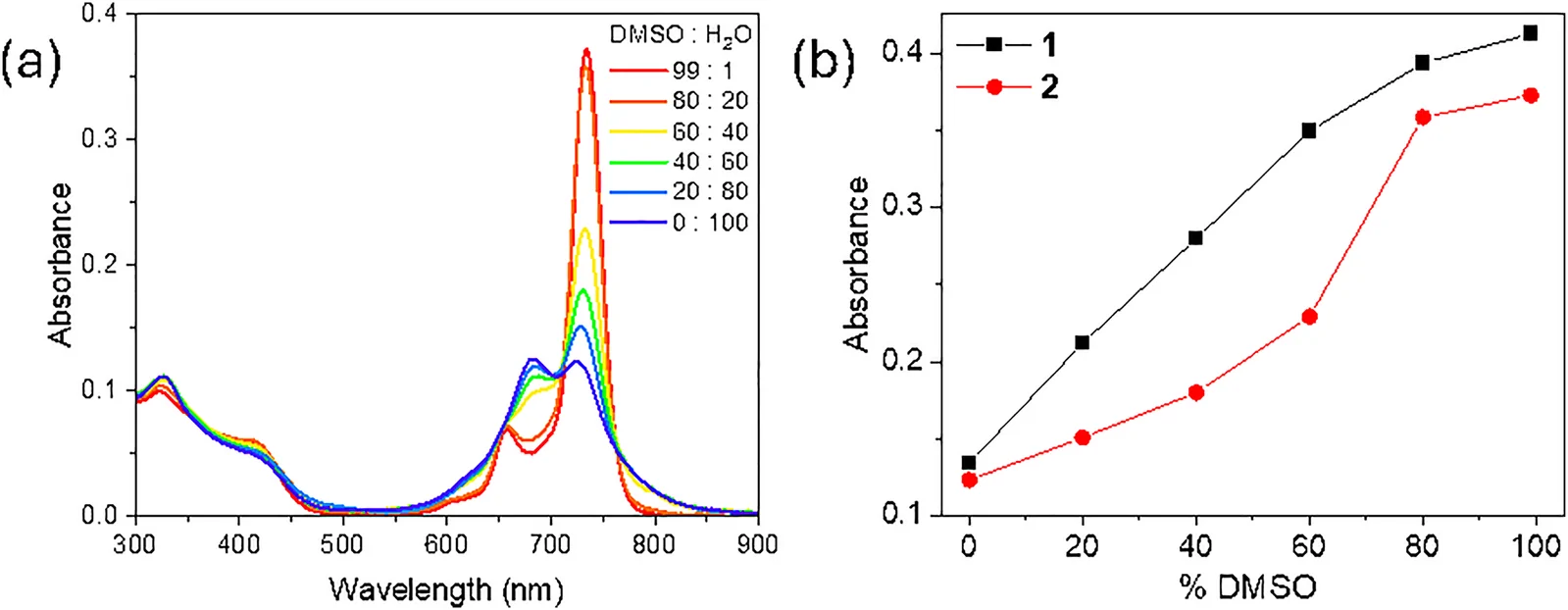
(a) UV-visible absorption spectrum of 2 in varying DMSO : H_2_O ratios.(b) Absorbance of Q band for 1 (737 nm, 2.3 µM) and 2 (734 nm, 2.5 µM) for given volume % DMSO in H_2_O.

Turning attention to the copper analogue, the absorption profiles of 2 in DMSO, H_2_O and their mixtures (Fig. 6a) are similar to 1 (Fig. S1a). In DMSO, 2 has a Soret band absorbance at 480 nm and a sharp Q_00_ band with a maximum at 734 nm (*ε* = 1.50 × 10^5^ M^−1^ cm^−1^) and a Q_01_ band at 658 nm that is characteristic for a monomer. In H_2_O, the Q band is weaker and blue-shifted to 726 nm and an additional band due to H-type aggregation is observed at 683 nm with a long tail beyond 800 nm. In DMSO/H_2_O mixtures, as the volume percentage of DMSO is decreased, the absorption band at 735 nm decreases due to an increasing population of aggregates. There is a significant spectral change between 80% and 60% DMSO with a significantly more aggregated profile at 60% DMSO than for 1 (Fig. 6b). This indicates that 2 potentially has a greater tendency to aggregate than 1, as a higher percentage of DMSO is required to disrupt these aggregates. The DLS autocorrelation coefficient in DMSO and H_2_O show scattering of aggregates in H_2_O but not in DMSO (Fig. S15), as we previously reported for 1, which indicates that similar aggregation behaviour is occurring for both complexes 1 and 2 in these two solvents.

**Fig. 7:**
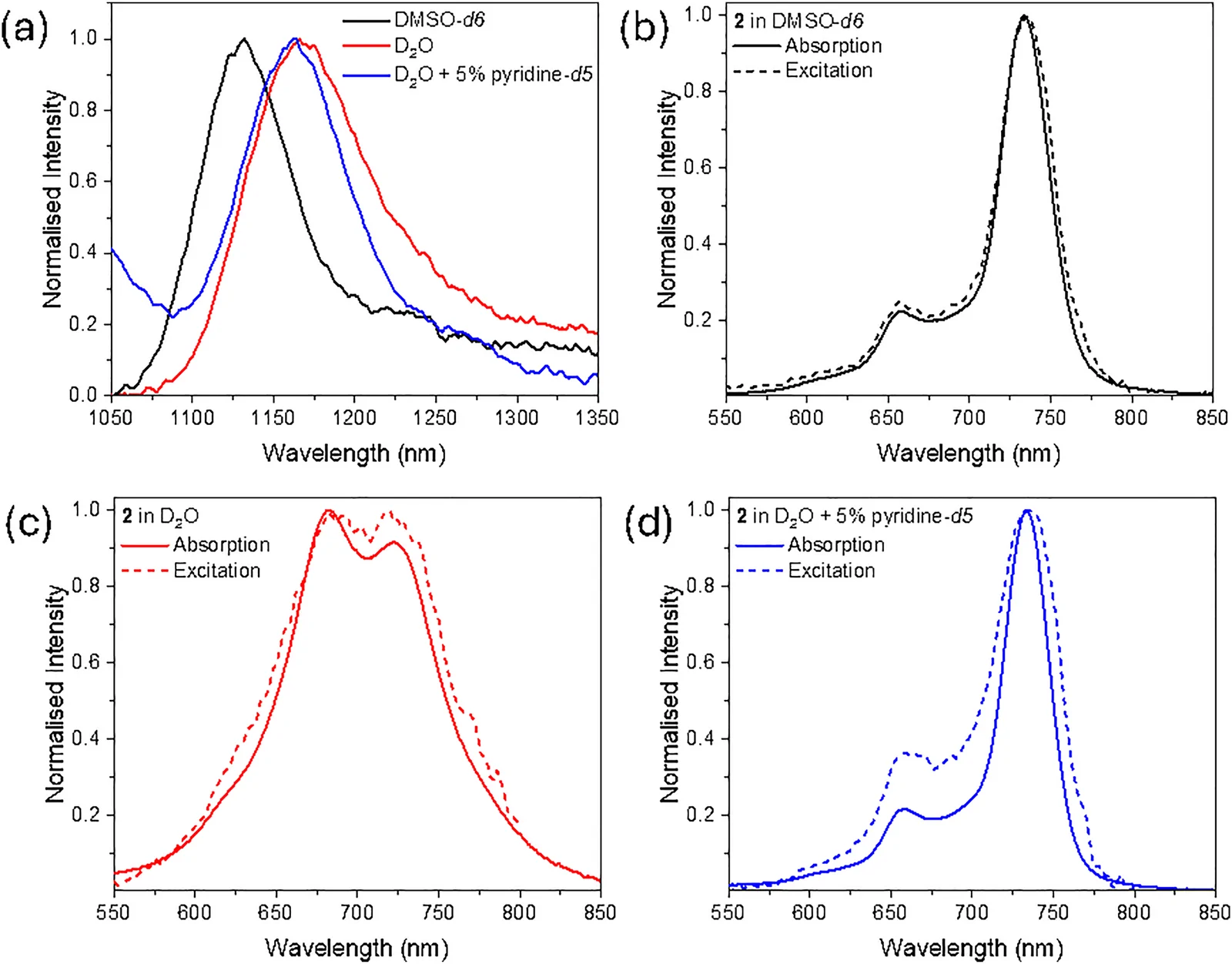
(a) Normalised emission spectra (*λ*_ex_ = 400 nm) of 2 in DMSO-d_6_ (1.5 µM), D_2_O (5.3 µM), and D_2_O with 5% pyridine-d_5_ (5.3 µM). Normalised absorption and excitation spectra for 2 in (b) DMSO-d_6_ (*λ*_em_ = 1130 nm), (c) D_2_O (*λ*_em_ = 1168 nm), (d) D_2_O with 5% pyridine-d_5_ (*λ*_em_ = 1162 nm).

The ground and excited states of 2 are labelled using Gouterman's nomenclature originally developed for copper porphyrins and later adapted to CuPcs with the total multiplicity added as a leading superscript to the Pc ligand state label.^43,44^ This emphasises the weak coupling of the Pc singlet and triplet excited states with the unpaired electron of the d^9^ Cu(ii) ion to give sing-doublet (^2^S_0/1_), trip-doublet (^2^T_1_) and trip-quartet (^4^T_1_) states. Unlike compound 1, 2 is non-fluorescent due to ultrafast ISC from ^2^S_1_ to ^2^T_1_ induced by the spin–orbit coupling of the paramagnetic Cu(ii) d^9^ centre.^45^ However, 2 does exhibit RT phosphorescence at 1132 nm in DMSO-d_6_, 1168 nm in D_2_O, and 1164 nm in D_2_O with 5% pyridine-d_5_, see Fig. 7. Interestingly, the excitation spectra in both DMSO-d_6_ and in D_2_O measured at the phosphorescence emission peak correspond to the respective absorption profiles, meaning that, unlike the fluorescence of 1 that only originates from monomers, aggregated 2 is phosphorescent, presumably alongside the small population of monomer. While fluorescence from suitably orientated ZnPc dimers has been reported,^46^ emission from aggregated Pcs is in general rare due to efficient quenching, Aggregates of the parent CuPc have been proposed to exhibit phosphorescence redshifted by approximately 320 cm^−1^ relative to the monomer.^47^ This is consistent with our observations for 2, where the emission spectra in DMSO-d_6_ and D_2_O differ by 270 cm^−1^. However, the earlier assignment of aggregate emission for CuPc remained inconclusive due to the lack of excitation spectra.

Emission from 2 at RT is likely from ^2^T_1_, thermally populated from the slightly lower energy ^4^T_1_ state. 2 has an almost monomeric absorption profile in D_2_O with 5% pyridine-d_5_, see Fig. 7d, indicating pyridine can effectively disrupt aggregates of the Cu(ii) complex. However, the phosphorescence excitation profile in this solvent mixture was slightly broader than the absorption profile and had greater relative intensity at 650 nm. Either the aggregates have a higher emission quantum yield than the monomer or the emission wavelength selected had a greater contribution from the aggregate, but either way it points to a small population of aggregate likely remaining under these conditions.

**Fig. 8:**
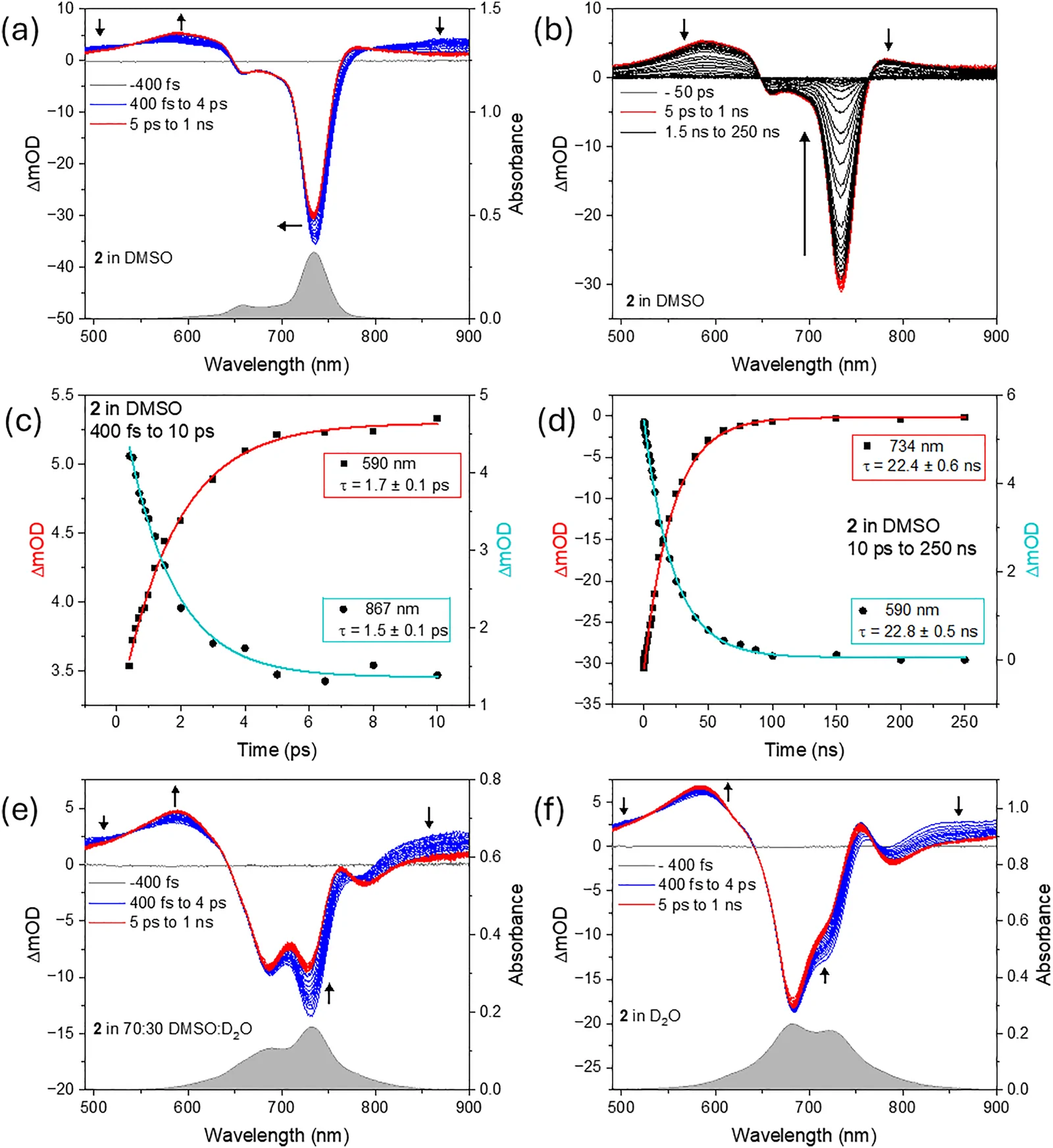
TA spectra and kinetics of 2 in DMSO (a–d, 24 µM), and TA spectra in 70 : 30 DMSO : D_2_O (e, 24 µM) and D_2_O (f, 48 µM) over various timescales (*λ*_ex_ = 400 nm, pump power = 120–200 nJ). In a, e and f, the steady-state absorption spectrum at the same concentration as the respective TA spectrum is shown in grey (right axes). In c the error on the mathematical fit is reported; for this short lifetime process, the uncertainty including the instrument response is approximately 0.2 ps (Fig. S30), which is reflected in the reported values in Table 4.

Fig. 8a and b show the TA spectra (*λ*_ex_ = 400 nm) of 2 in DMSO at early (400 fs to 1 ns) and late (5 ps to 250 ns) times. The initial Q band bleach has a maximum at 736 nm that then blue shifts to 734 nm over 5 ps. This may be due to changes in the transient band over this time. Similar to 1 in DMSO, there is an initial change in profile of the broad transient bands either side of the GSB; however, this process is much faster for 2 than 1. Between 400 fs and 4 ps, the band intensity increases at 590 nm and decreases in intensity at 510 nm while the lower energy transient band increases in intensity at 750 nm and decreases in intensity at 867 nm with time constants of 1.5–1.7 ps (Fig. 8c). In this time, the GSB band at 688 nm does not show significant recovery. Therefore, this early process can be interpreted as an ultrafast ISC leading to new ESAs of the thermally equilibrating ^4^T_1_ and ^2^T_1_ states. The resulting profiles after 10 ps are very similar to the transient profile of 1 in DMSO at 11.5 ns when the triplet state is populated (Fig. S16); for 1, only small changes in profile occurred prior to 100 ps, which highlights the dramatic difference in kinetics of ISC between these two species. As shown in Fig. 8b and 8d, 2 then fully recovers with a lifetime of 22.8 ns, as measured by recovery of the ESA at 590 nm, that is similar to the time constants for relaxation of the ^4/2^T_1_ states of other CuPcs.^48^ The triplet lifetime of 2 is *ca.* four orders of magnitude shorter than that of 1. Both the ISC and the relaxation to the ground state are therefore clearly significantly enhanced by the spin–orbit coupling of the paramagnetic Cu(ii) centre.

Fig. 8e and f show the TA spectra of 2 in a 70 : 30 (v/v) DMSO : D_2_O solvent mixture and D_2_O at the early times out to 1 ns. The initial spectra at 400 fs reflect the ground state absorption profiles; however, this changes rapidly over the initial 4 ps. In the solvent mixture, the peak at 731 nm rapidly decreases as the transient band at 764 nm changes in profile. This is overlapping with the bleach band, and the combination of the GSB and ESA does not rise above Δ*A* = 0. In 100% D_2_O, there is a similar fast loss of the band at 717 nm and a more distinct transient band at 755 nm that overlaps the GSB band that is visible again at ∼780 nm. This transient band appears to be narrower than the broad band observed below 650 nm; however, this may be due to overlap with the strong bleach band. This band may involve charge transfer with the open-shell Cu(ii) centre^22^ or due to ESA from ^2^T_1_/^4^T_1_. Similar to that observed in DMSO, full recovery of ground state 2 then takes place over 250 ns. The kinetics of recovery of 2 in DMSO, 70 : 30 (v/v) DMSO:D_2_O, D_2_O, and H_2_O are shown in Table 4 and Figures S17–S19. The ISC time constant is reduced to 1.2 ps in the solvent mixture and is further reduced in D_2_O and H_2_O to 0.8 ps. The lifetimes of this recovery in D_2_O and H_2_O are shorter than in DMSO at 19.7 and 14.0 ns respectively with a notable shortening of the lifetime observed for quenching H_2_O. No significant difference was observed for the ISC lifetime indicating it is only the back ISC relaxation pathway that is impacted by this isotope exchange. The lifetime is slightly extended in the 70 : 30 DMSO:D_2_O solvent mixture at 28.1 ns. This indicates that the long-lived ^2^T_1_/^4^T_1_ states are being accessed in all these solvent systems.

**Table 4:** Lifetimes of grow in and recovery of transient features of 2 in different solvent environments assigned to intersystem crossing and recovery of the ground state

Solvent	*τ* (ISC) (ps)	*τ* (^4/2^T_1_ → ^2^S_0_) (ns)
DMSO	1.7 (±0.2)^[1]^	22.8 (±0.5)^[1]^
70 : 30 DMSO : D_2_O	1.2 (±0.2)^[2]^	28.1 (±0.6)^[1]^
D_2_O	0.8 (±0.2)^[3]^	19.7 (±0.4)^[1]^
H_2_O	0.8 (±0.2)^[4]^	14.0 (±0.2)^[1]^

**Fig. 9:**
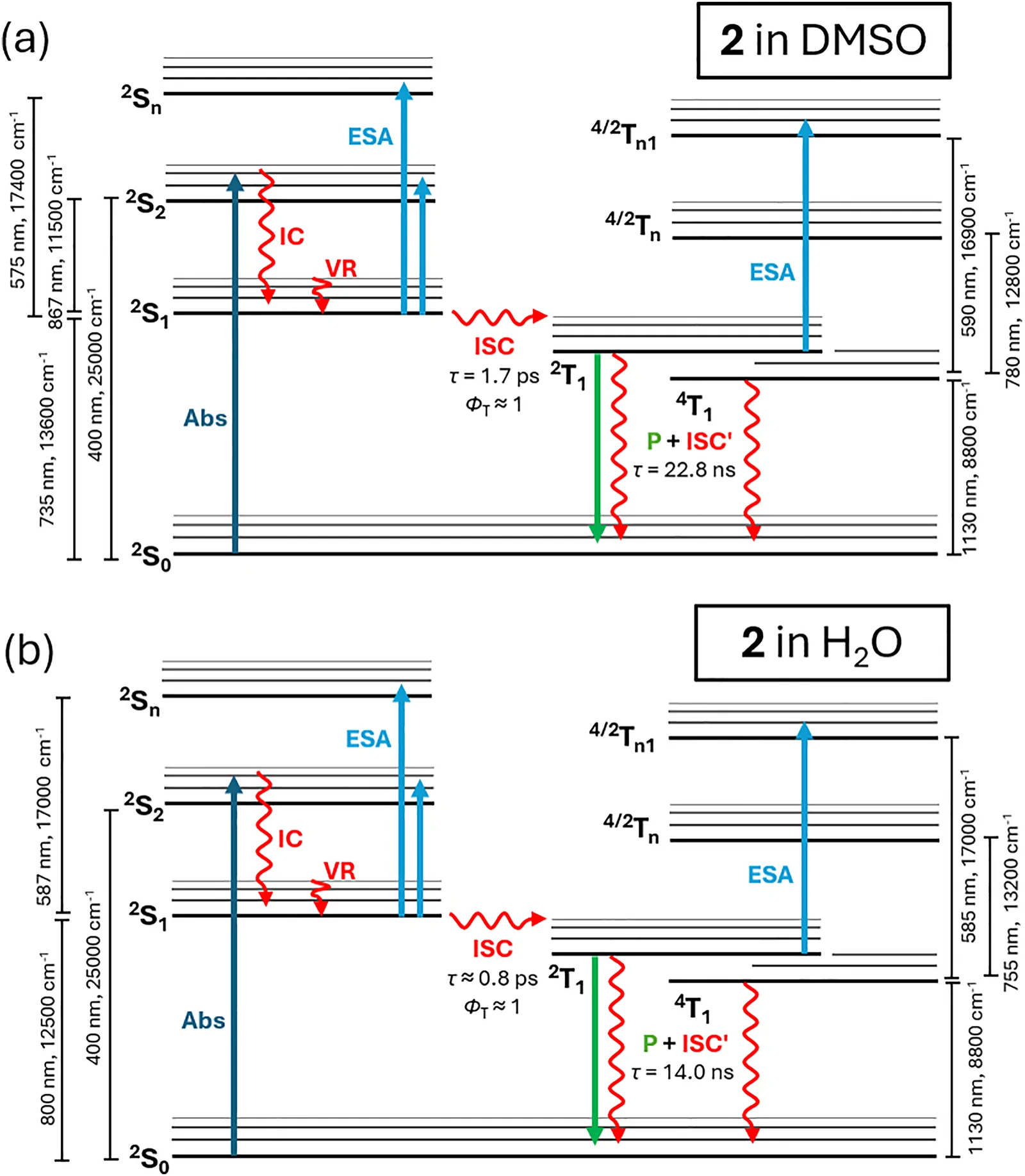
Simplified Jablonski Diagram of 2 in (a) DMSO and (b) H_2_O. Solid arrows show radiative processes. Dark blue arrow shows absorption at the pump wavelength. Light blue arrows show absorption of the excited states. Red wavy arrows show non-radiative processes. Green arrow shows radiative relaxation. Approximate energy differences between states, time constants, and quantum yields shown.

The lifetime of 2 in D_2_O is similar to the lifetime in DMSO (∼20 ns) indicating a similar state is being accessed in both cases. This is also supported by the similarity of the spectral changes in the transient bands (Fig. S20). This time constant is similar to the phosphorescence lifetime reported for Cu(ii)Pc derivatives.^49^ In comparison, the lifetime of 1 in DMSO is over six orders of magnitude greater than in D_2_O. This indicates that ^2^S_1_ → ^2^T_1_ ISC is favoured by incorporation of the paramagnetic Cu(ii) ion. Fast ISC rates mean the long-lived state is formed rapidly, competing favourably with the fast internal conversion from the singlet excited state when aggregated. The Cu(ii) also speeds up back ISC deactivation of the long-lived excited state explaining why this lifetime is significantly shorter than the Zn(ii) triplet lifetime. In comparison, the Zn(ii) centre can access the triplet excited when monomeric with slower ISC rates (on the order of 2 ns) but fast non-radiative relaxation when aggregated prevents triplet excited states being accessed in D_2_O. Fig. 9 summarises the TA data for 2 in the form of Jablonski diagrams.

**Fig. 10:**
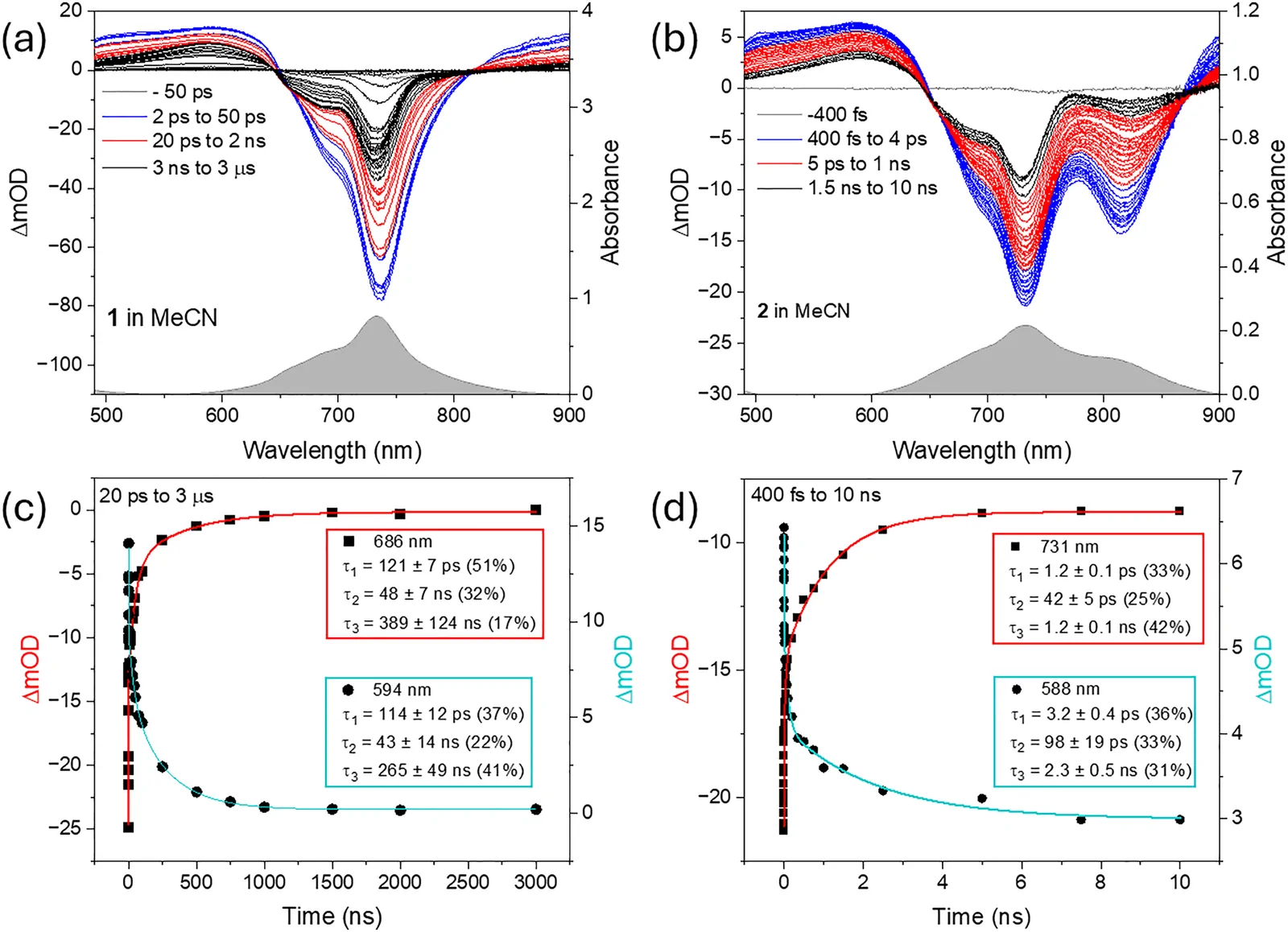
TA spectra and kinetics of 1 (60 µM) and 2 (24 µM) in MeCN (*λ*_ex_ = 400 nm, pump energy = 200 nJ) (a) TA spectra of 1 between 2 ps and 3 µs. (b) TA spectra of 2 in MeCN between 400 fs and 10 ns. Recovery of GSB and ESA bands for (c) 1 between 20 ps and 3 µs and (d) 2 between 400 fs and 10 ns. Solid lines show exponential fits. In a and b, the steady-state absorption spectrum at the same concentration as the respective TA spectrum (right axes).

The behaviour of 1 and 2 (X = I) in another organic solvent, acetonitrile (MeCN), was next examined. In this solvent the compounds are less soluble than in DMSO and require heating to dissolve at the concentrations required for TA even with the more MeCN soluble iodide salts. The UV-visible absorption profile of 1 in MeCN is most similar to, though still distinct from, the spectrum recorded in the 50 : 50 (v/v) DMSO/D_2_O mixture, indicating a mixture of aggregates and monomers are present in MeCN, as shown in Fig. 10a. The bleach band in the TA spectrum of 1 in air-equilibrated MeCN has a maximum absolute intensity at 738 nm and a shoulder at 686 nm. The evolution of the TA profile is quite different to that of 1 in the DMSO/D_2_O solvent mixture where the aggregate band was lost more rapidly, and a monomer bleach band was observed at 6 µs. In MeCN, a purely monomeric profile is not observed at any time prior to full recovery. Very different kinetics are observed with quite complex fittings, which may indicate a mixture of species is present, as shown in Fig. 10c and S21. The bleach band at 696 nm recovers with two initial fast processes occurring with lifetimes of 2.4 and 121 ps, followed by a longer process with lifetimes of 48 and 389 ns. 1 in aerated MeCN is fully recovered by 2 µs, which is quicker than in aerated DMSO (*τ* = 3 µs), reflecting the higher diffusion constant for O_2_ in MeCN than in DMSO, leading to more efficient quenching in MeCN, despite a lower O_2_ concentration in this solvent.

The ground-state absorbance of 2 in MeCN was dramatically different to the profile in DMSO or aqueous solution as shown in Fig. 10b. A Q band absorption at 733 nm with a small shoulder at 697 nm and a more distinct shoulder at 800 nm was recorded. The TA spectrum shows a similar spectrum with a sharper maximum observed at 817 nm. Fig. S22 shows the biexponential kinetic fits of the Q band GSB between 400 fs and 10 ns at 731 nm and the 588 nm transient of 2 in MeCN. Fig. S22b shows the recovery of the additional bleach band at 816 nm. These three bands recover with complex kinetics, probably reflecting the sample inhomogeneity in MeCN, with lifetimes for these faster processes between 1.2–3.2 ps, 42–98 ps and 1.2–2.3 ns. A transient beyond 900 nm (Fig. 10b), at the edge of our probe range, appears to decay faster than the transient at 588 nm, but interpretation of this feature is hampered by significant overlap with the bleach band.

Fig. S22b shows the recovery of 2 in MeCN over longer times between 11–250 ns. The transient at 588 nm and Q band bleach at 731 nm do not fully recover by 250 ns, unlike 2 in the solvent environments discussed previously. In contrast, the additional bleach band at 816 nm recovers fully by 100 ns, resulting in a large change in profile. The recovery of the bands at 816, 731 and 588 nm were fit with lifetimes of 34, 186 and 82 ns respectively (Fig. S22b and d). This apparent inconsistency may be explained by the poor solubility in acetonitrile leading to the formation of a range of different aggregate species with substantially different excited-state dynamics. It was particularly challenging to achieve high-quality fits to these data, especially at 731 nm. In any case, these triplet lifetimes are all substantially longer than those measured in any other solvent, indicating the environment and speciation can significantly impact the excited-state behaviour of 2. A summary table of the kinetics of 2 and a comparison of its vastly different excited-state dynamics to 1 can be found in Table S1.

## Conclusion

In this study, we have investigated the excited-state dynamics of two metallo-phthalocyanines, ZnPc 1 and CuPc 2, in a range of solvents that either promote their monomeric or aggregated forms over a continuous range of 100s fs to 100s µs using the time-resolved multiple-probe transient-absorption spectroscopy approach. In DMSO a monomeric phthalocyanine is formed in both cases, which in the case of 1 has an emissive singlet excited state with a lifetime of approximately 1.9 ns, as measured by both TCSPC and TAS. ISC leads to a long-lived (196 µs) triplet state that can sensitize singlet oxygen. On the other hand, 2 undergoes ultrafast ISC to a trip-doublet (^2^T_1_) state in 1.7 ps in DMSO and less than a picosecond in water. This ^2^T_1_ state then persists with a lifetime of *ca.* 20–23 ns and is found to be phosphorescent at RT. In solvents that predominantly promote aggregation, namely water, 50 : 50 mixtures of D_2_O/DMSO, or MeCN, very different dynamics are observed. The dynamics are found to depend on the deuteration of water but not of DMSO or the methyl groups of 1. The fluorescence of 1 is quenched in water due to aggregation but interestingly phosphorescence is seen from 2 even in the aggregated state, as evidenced by excitation spectra, with a lifetime similar to the monomeric form. In the aggregated form, 1 undergoes rapid internal conversion, and thus is expected to be capable of generating a local heating effect in this form. Understanding the behaviour of different phthalocyanine species in solution will be critical for predicting their behaviour in phototherapeutic applications and can evidence a switch from a photodynamic to a photothermal mechanism of action depending on aggregation state. In future studies we will investigate the potential of these phthalocyanines as photosensitisers for phototherapy.

## Conflicts of interest

There are no conflicts to declare.

## Supplementary Material

CP-028-D6CP02573K-s001

## Data Availability

The authors confirm that the data supporting the findings of this study are available within the article and its supplementary information (SI). Supplementary information includes details of materials, instrumentation and methods including synthetic details, additional plots of steady-state and transient-absorption spectra and a summary table of the excited-state dynamics of 1 and 2. See DOI: https://doi.org/10.1039/d6cp02573k.
