# Supplementary material for: Femto- to microsecond excited-state dynamics of aggregated and monomeric regiopure zinc and copper phthalocyanines
Source: Phys Chem Chem Phys. 2026 Aug 25;28(36):22361–73. doi: 10.1039/d6cp02573k (PMC13599766; doi:10.1039/d6cp02573k)
Supplement: Supplementary file 1 [file CP-028-D6CP02573K-s001.pdf]

Supplementary Information (SI) for Physical Chemistry Chemical Physics.
This journal is © the Owner Societies 2026

Supporting Information

Femto- to Microsecond Excited-State Dynamics of Aggregated and Monomeric Regiopure Zinc and Copper Phthalocyanines

Eleanor R. Windle,[1] Donald G. Mackay,[2] Daniel Graczyk,[1] Christopher C. Rennie,[2] Partha Malakar,[3] Susan J. Quinn,[1]* Robert M. Edkins[2]*

E-mail: susan.quinn@ucd.ie, robert.edkins@strath.ac.uk

[1] School of Chemistry, University College Dublin, Dublin 4, Ireland.

[2] Department of Pure and Applied Chemistry, University of Strathclyde, Glasgow, Scotland, UK.

[3] Science and Technology Facilities Council, Rutherford Appleton Laboratory, Didcot, Oxfordshire, OX11 0QX, United Kingdom

---

Contents

Experimental	S2–S6
Materials	S2
Instrumentation and Methods	S2–S3
Transient Absorption Spectroscopy (TAS)	S3–S5
Synthesis	S5–S7
Spectroscopic data	S8–S27
References	S27

Experimental

Materials

Deionized water (18.2 MΩ cm) was obtained from an ultra-pure Millipore filtration system. Solvents and reagents were purchased from Fisher Scientific, Merck, or VWR. Compounds **TEGPyrPc**[1] and **[ZnTEG^{Q}PyrPc]I$_4$**[2] were made according to our previous reports. Solvents were removed under reduced pressure using a rotary evaporator at a maximum temperature of 40 °C.

Instrumentation and Methods

Liquid chromatography-mass spectrometry (LC-MS) with electrospray ionization (ESI) was recorded using an Agilent 6130 spectrometer with 1200 series LC with UV detection at 254 nm with an Agilent Poroshell 120 LC column (EC C18 2.7 μm, 4.6 mm x 75 mm). Accurate MS data was recorded using a Thermo Scientific Exactive Plus OrbiTrap with ESI ionization.

Solution NMR spectroscopic data were obtained at room temperature (r.t.) unless otherwise specified in aerated, deuterated solvents using an AVIII 400 or AVIII HD 500 Bruker spectrometer. ^{1}H and ^{2}H NMR chemical shifts (*δ*) were referenced to residual protiated or deuterated solvent. Standard multiplet abbreviations apply. FTIR spectra were recorded using a Perkin Elmer Spectrum One FTIR spectrometer and signals reported in cm^{-1}. UV-visible absorption spectra were recorded at r.t. using a Cary 50 UV-Vis spectrophotometer or Agilent Cary 60 spectrophotometer (Cary WinUV software) using background correction. Temperature controlled UV-vis spectra were recorded using a Cary 3500 Multicell UV-Vis spectrophotometer using block heating. The molar extinction coefficient was determined from the Beer-Lambert law: $A = \varepsilon c l$, with solution concentrations in DMSO of 10 μM, 5 μM, 2.5 μM, 1.25 μM. Solutions were made from three independently weighed samples on a six decimal place balance. The extinction coefficient was calculated from these data by linear regression of absorbance vs. concentration in Microsoft Excel. The complex **1** was found to obey the Beer–Lambert Law in this concentration range in DMSO. Emission spectra were recorded using a Varian Cary Eclipse spectrofluorometer (Cary Scan software, λ_{ex} = 650 nm, slit widths = 10 nm unless otherwise stated) operating in right-angle geometry mode or an Edinburgh Instruments FLS1000 spectrometer operating in front-facing or right-angle geometry mode, equipped with a double monochromator for both excitation and emission and an NIR-sensitive InGaAs photomultiplier tube. For emission measurements, all solutions were made to an absorbance lower than or equal to 0.2 to minimise the inner filter effect, unless otherwise

stated. All spectra were fully corrected for the spectral response of the instrument. Low-temperature fluorescence measurements were achieved using an Oxford Instruments OptistatDN-X sample in exchange gas (He) optical cryostat cooled with liquid nitrogen cooling. Temperature control was achieved using an Oxford Instruments MercuryiTC temperature controller. Circular dichroism spectra were collected on a Jasco J-810 spectropolarimeter between 200-400 nm at a scanning speed of 100 nm min^{-1} averaging over 8 scans. Dynamic light scattering measurements were carried out on a Malvern Zetasizer Nano-ZS with a 4 mW He-Ne laser operating at 632.8 nm and measurements were recorded at 173°. The fluorescence quantum yield of **1** in DMSO was measured by the relative method using zinc phthalocyanine (Φ_f = 0.17 in acetone)[2] as a suitable standard and calculated using the Eq. S1, which takes into account the change of solvent.

$$\Phi_S = \Phi_R \cdot (Grad_S / Grad_R) \cdot (\eta_S / \eta_R)^2 \qquad \text{Eq. S1}$$

where S and R subscripts are for the sample and reference, respectively, and Φ is the fluorescence quantum yield, *Grad* the gradient of a plot of integrated emission intensity against absorbance measured at various concentrations, and η is the solvent refractive index.

The absolute fluorescence quantum yield of **1** in H_2O was measured on an Edinburgh Instruments FS5 Spectrofluorometer equipped with an SC-30 integrating sphere module. Solution samples and the corresponding solvent blanks were measured in a 1 cm quartz cuvette using direct excitation (λ_{ex} = 400 nm). Due to the low intensity of the emission, the scatter peak was recorded separately using an NDF and adjusted using the % transmission at 400 nm. The absolute quantum yield was calculated in Fluoracle using the Quantum Yield Wizard.

Transient Absorption Spectroscopy (TAS)

Transient absorption spectra were recorded using the LIFEtime laser system at the STFC Rutherford Appleton Laboratory with full details given in reference 3. Briefly, an Yb:KGW ultrafast oscillator (80 MHz) seeded two Pharos Yb:KGW amplifiers (Light Conversion Ltd., 100 kHz, (**1**) 15 W, 260 fs, (**2**) 6 W, 180 fs output pulses). The output from amplifier 1 pumped an Orpheus HP OPA (Light Conversion Ltd.) tuned to 400 or 680 nm as the pump beam (200 nJ/pulse, 1 kHz with pulse picking), which was passed along a 0–12 ns multi-pass optical delay stage before focusing (120–150 μm diameter) at the sample. Part of the output of the second amplifier was used to create the white-light continuum probe (490–920 nm) pulses at 100 kHz by focussing 1030 nm fundamental into a sapphire crystal. The WLC was detected

using a linear silicon array (Teledyne Vision Solutions). The probe was spatially overlapped with the pump at the sample. Using the time-resolved multiple probe spectroscopy (TRMPS) method[3] pump-probe delays up to 0.5 ms were achieved using a combination of optical and electronic delays and later probe pulses in the train. Typically, three-second averaging with three repeat cycles was performed for each time delay. Samples with an absorption of ca. 0.2–0.3 at the Q band in 1 mm quartz cuvettes were raster scanned to minimise photodamage. UV-visible absorption spectra were recorded before and after measurements using a Cary 60 spectrophotometer, which confirmed that there was no to only minor sample photodegradation during TAS measurements. For early delays, a correction was carried out to account form for the temporal chirp. The chirp present in the probe spectra was corrected by taking the half-rise point of each wavelength as time zero. Then, each kinetic trace was interpolated using a linear method for the measured time points. All spectra were processed using the in-house Ultraview software provided by the Central Laser Facility (CLF). The samples were calibrated by absorption of WCT2065 and converted from pixels into wavelength using the Ultracal software. Data were analysed spectrally at the different time delays and kinetically by fitting one or more exponentials to the data at a specific wavelength associated with the ground-state bleach (GSB) or excited-state absorption (ESA) feature of interest in Origin 2024 using equations 1 – 3.

$$y = y_0 + A_1 e^{-\frac{x}{\tau_1}} \tag{1}$$

$$y = y_0 + A_1 e^{-\frac{x}{\tau_1}} + A_2 e^{-\frac{x}{\tau_2}} \tag{2}$$

$$y = y_0 + A_1 e^{-\frac{x}{\tau_1}} + A_2 e^{-\frac{x}{\tau_2}} + A_3 e^{-\frac{x}{\tau_3}} \tag{3}$$

where y is ΔOD recorded at time delay x, y_0 is ΔOD at time zero (x = 0), τ_1, τ_2, and τ_3 are fitted time constants, A_1, A_2, and A_3 are weighting functions for each exponential process.

To determine the instrument response function (IRF) in the different solvent systems investigated, re-convolution analysis of the earliest-time transient absorption kinetics measured with 400 nm excitation in a 1 mm cuvette was performed using a Python script.[4] Representative datasets collected in each solvent were fitted over the vicinity of time zero at probe wavelengths of 734 nm or 706 nm using a Gaussian instrument response function with a free fit on the half width convoluted with the relevant kinetic model. The time constants discussed throughout the manuscript are substantially longer than the measured IRF FWHM of 292 fs in DMSO, 254 fs in H_2O and 253 fs in MeCN.

Synthesis

1,8,15,22-Tetra(1,4,7,10-tetraoxaundecyl)-3,10,17,24-(4′-pyridyl) copper(II) phthalocyanine, CuTEGPyrPc

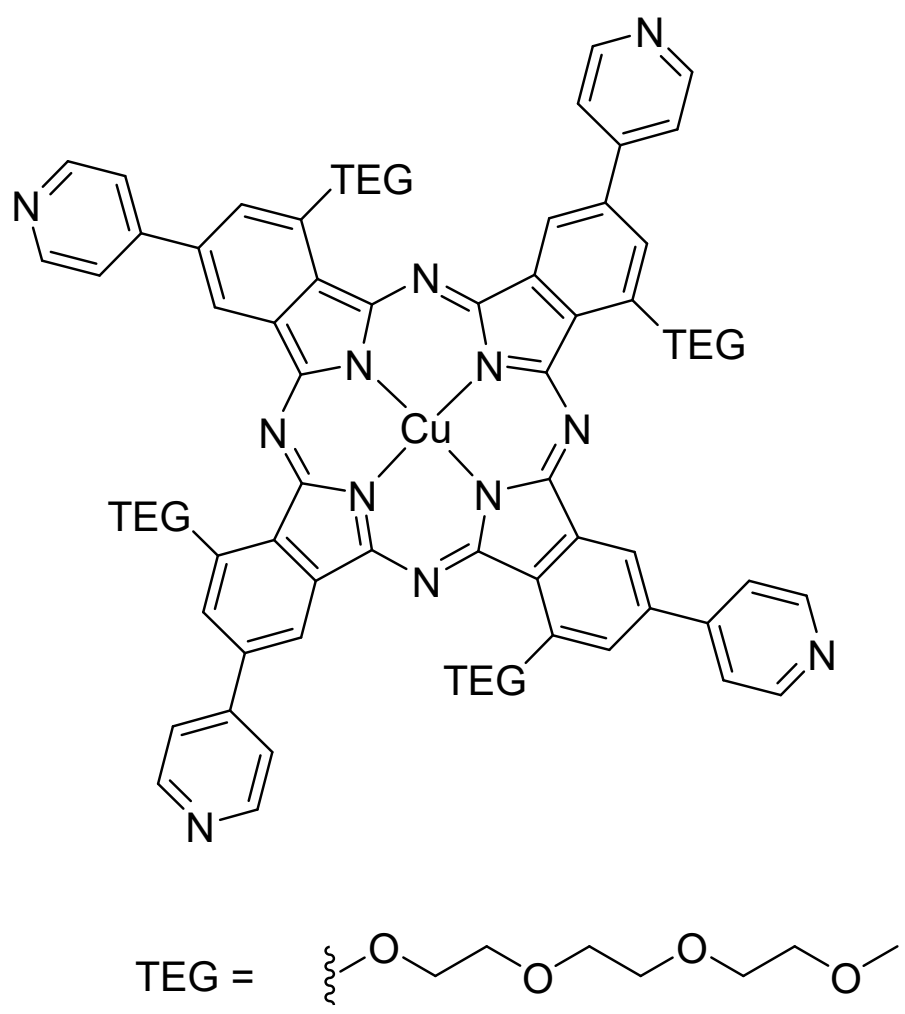

A solution of **TEGPyrPc** (85 mg, 58 µmol) in DMF (30 mL) was degassed by three vacuum/N_2-backfill cycles in a Schlenk flask followed by the addition of $Cu(OAc)_2{\cdot}H_2O$ (116 mg, 0.58 mmol, 10 eq.). The reaction was heated to 150 °C for 24 h. After this time, the reaction mixture was allowed to cool to room temperature. The product was precipitated from solution using excess diethyl ether. The precipitate was then vacuum filtered, and the residue was washed with further portions of diethyl ether, acetone, $CHCl_3$ and finally MeOH to yield the title compound as a green solid (65 mg, 73% yield). **FTIR (cm^{-1}):** 2874, 2857, 1610, 1547, 1502, 1389, 1338, 1298, 1213, 1157, 1094, 1062, 1026, 917, 873, 818, 700. **UV-visible absorption:** λ_{max} (Q-band, DMSO) 718 nm. The compound does not follow Beer-Lambert law in any solvent system tested**.** *N.B.* the paramagnetic nature of the compound precludes meaningful NMR analysis.

1,8,15,22-Tetra(1,4,7,10-tetraoxaundecyl)-3,10,17,24-(*N*-methyl-4′-pyridinium) copper(II) phthalocyanine tetraiodide, [CuTEG^{Q}PyrPc]I$_4$

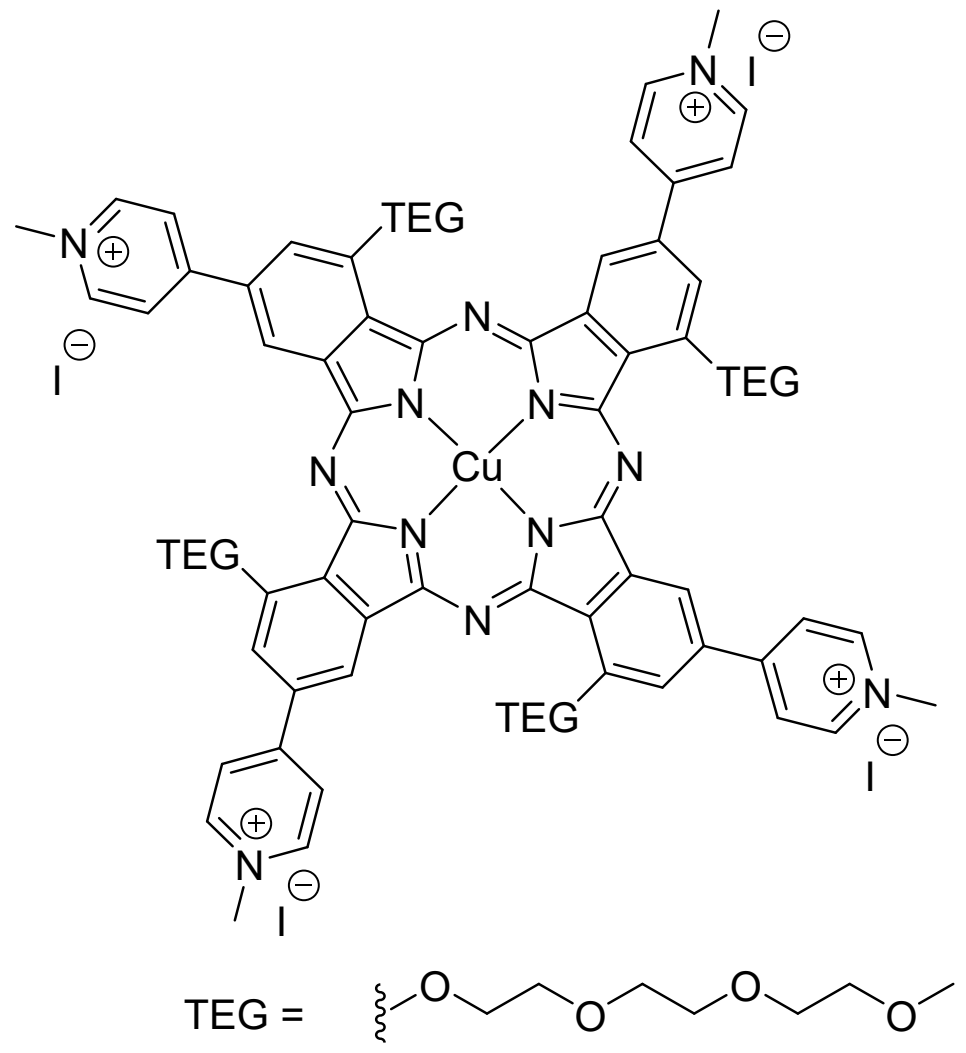

A solution of **CuTEGPyrPc** (60 mg, 0.025 mmol) in DMF (10 mL) was sparged with argon for 15 minutes in an oven-dried reaction vial sealed with a crimp cap containing a Teflon septum (Biotage, 10–20 mL microwave vial) before the addition of iodomethane (2.28 g, 16 mmol, 1.0 mL). The vessel was heated to 80 ºC for 24 h. The reaction was cooled to room temperature, and the product was precipitated by addition of excess diethyl ether and filtered through a PTFE syringe filter (0.45-μm pore size, 25-mm diameter). The crude residue was washed with diethyl ether, acetone, $CHCl_3$ and recovered from the syringe filter with water. The aqueous solution was lyophilized, resulting in isolation of the title compound as a green powder (59 mg, 72% yield).

ESI$^+$ MS (water): Found *m/z* $[M]^{4+}$ = 398.3, theoretical *m/z* $[M]^{4+}$ = 397.9. **ESI$^+$ HRMS (H_2O/0.1% formic acid):** found *m*/*z* = 397.9085 and 398.4087. Theoretical *m/z* $[C_{84}H_{96}N_{12}O_{16}{}^{63}Cu]^{4+}$ = 397.9085 (Δ = 0.0 ppm) and *m/z* $[C_{84}H_{96}N_{12}O_{16}{}^{65}Cu]^{4+}$ = 398.4081 (Δ = +1.5 ppm). **FTIR (cm^{-1}):** 3013, 2864, 1638, 1589, 1562, 1557, 1526, 1503, 1483, 1393, 1342, 1303, 1258, 1225, 1188, 1092, 945, 918, 831, 750**. UV-visible absorption:** λ_{max} (Q-band, DMSO) 734 nm (log(ε/M^{-1} cm^{-1}) = 5.18). *N.B.* the paramagnetic nature of the compound precludes meaningful NMR analysis.

[ZnTEG^{Q}PyrPc-d$_{12}$]I$_4$

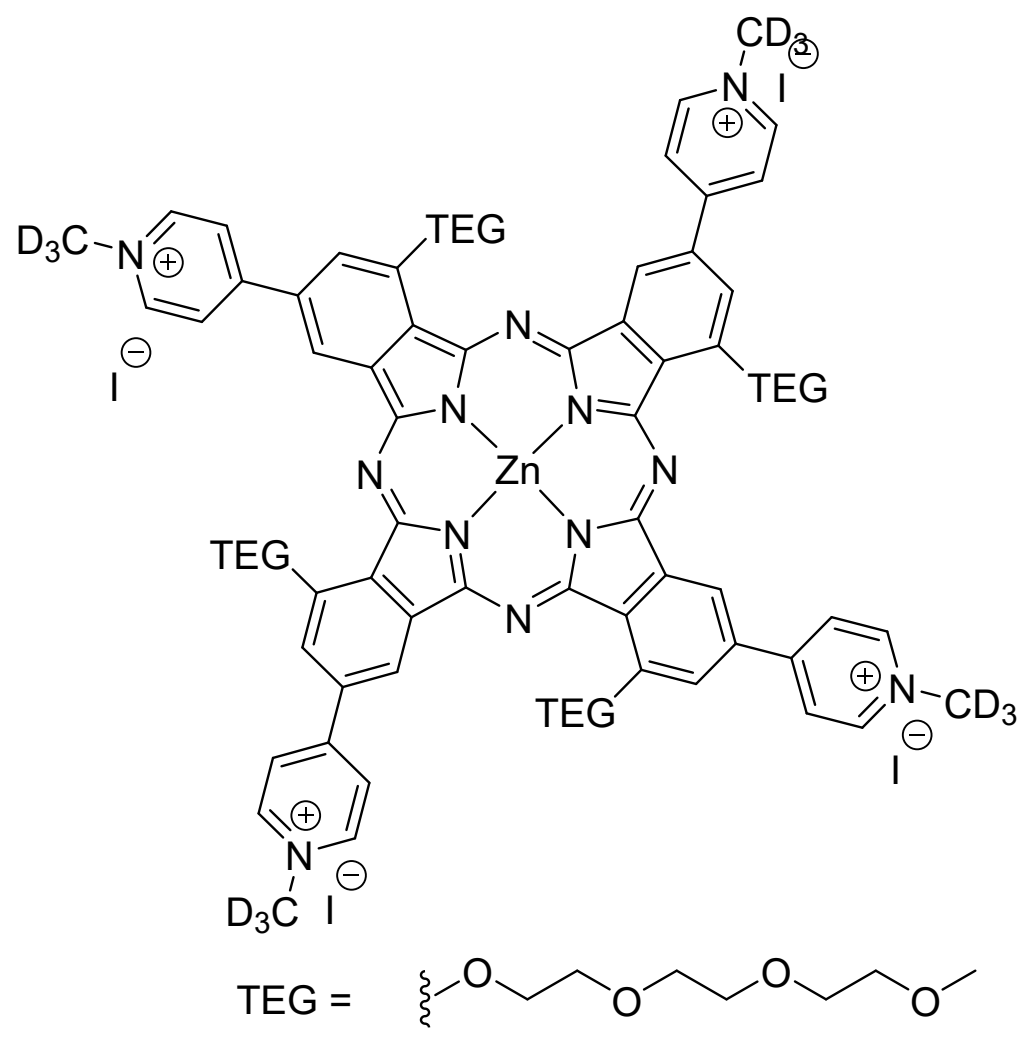

Methylation of the four 4-pyridyl groups of ZnTEGPyrPc following our previously reported method[2] but using iodomethane-d$_3$ instead of iodomethane. **^{1}H NMR** (500 MHz, DMSO-*d*$_6$): δ_H/ppm 9.59 (s, 4 H), 9.26 (d, $^3J_{HH}$ = 6.4 Hz, 8 H), 9.02 (d, $^3J_{HH}$ = 6.4 Hz, 8 H), 8.41 (s, 4 H), 5.18 (t, $^3J_{HH}$ = 4.6 Hz, 8 H), 4.57 (t, $^3J_{HH}$ = 4.6 Hz, 8 H), 3.99 (t, $^3J_{HH}$ = 5.1 Hz, 8 H), 3.64 (t, $^3J_{HH}$ = 5.1 Hz, 8 H), 3.44 (~t, $^3J_{HH}$ = 4.9 Hz, 8 H), 3.24 (~t, $^3J_{HH}$ = 4.9 Hz, 8 H), 3.02 (s, 12 H). **^{2}H NMR** (76.8 MHz, DMSO-*d*$_6$): δ_D/ppm 3.08 (s, 12 D). **ESI$^+$ MS (water):** Found *m/z* $[M]^{4+}$ = 401.5, theoretical *m/z* $[M]^{4+}$ = 401.8. **ESI$^+$ HRMS (H_2O/0.1% formic acid):** found *m*/*z* = 401.1768, 401.6770 and 402.1764. Theoretical *m/z* $[C_{84}H_{84}D_{12}N_{12}O_{16}{}^{64}Zn]^{4+}$ = 401.17725 (Δ = -1.1 ppm), $[C_{84}H_{84}D_{12}N_{12}O_{16}{}^{66}Zn]^{4+}$ = 401.67647 (Δ = +1.3 ppm), and $[C_{84}H_{84}D_{12}N_{12}O_{16}{}^{68}Zn]^{4+}$ = 402.17617 (Δ = -0.57 ppm).

Iodide to chloride anion exchange

Ca. 10 mg of the respective **[MTEG^{Q}PyrPc]I$_4$** (M = Cu or Zn) was dissolved in MeOH and stirred gently with 1 g of Amberlite IRA-402 resin (chloride form, basic, prewashed twice with MeOH) for two hours. The solution was filtered and the Amberlite resin replaced by a further 1 g of resin before stirring for a further two hours. This procedure was repeated a third time to ensure full conversion to **1**, **1-d$_{12}$** (M = Zn) or **2** (M = Cu) before the MeOH was removed under vacuum. UV-visible absorption spectroscopy was used to show that there was no degradation during this procedure.

Spectroscopic data

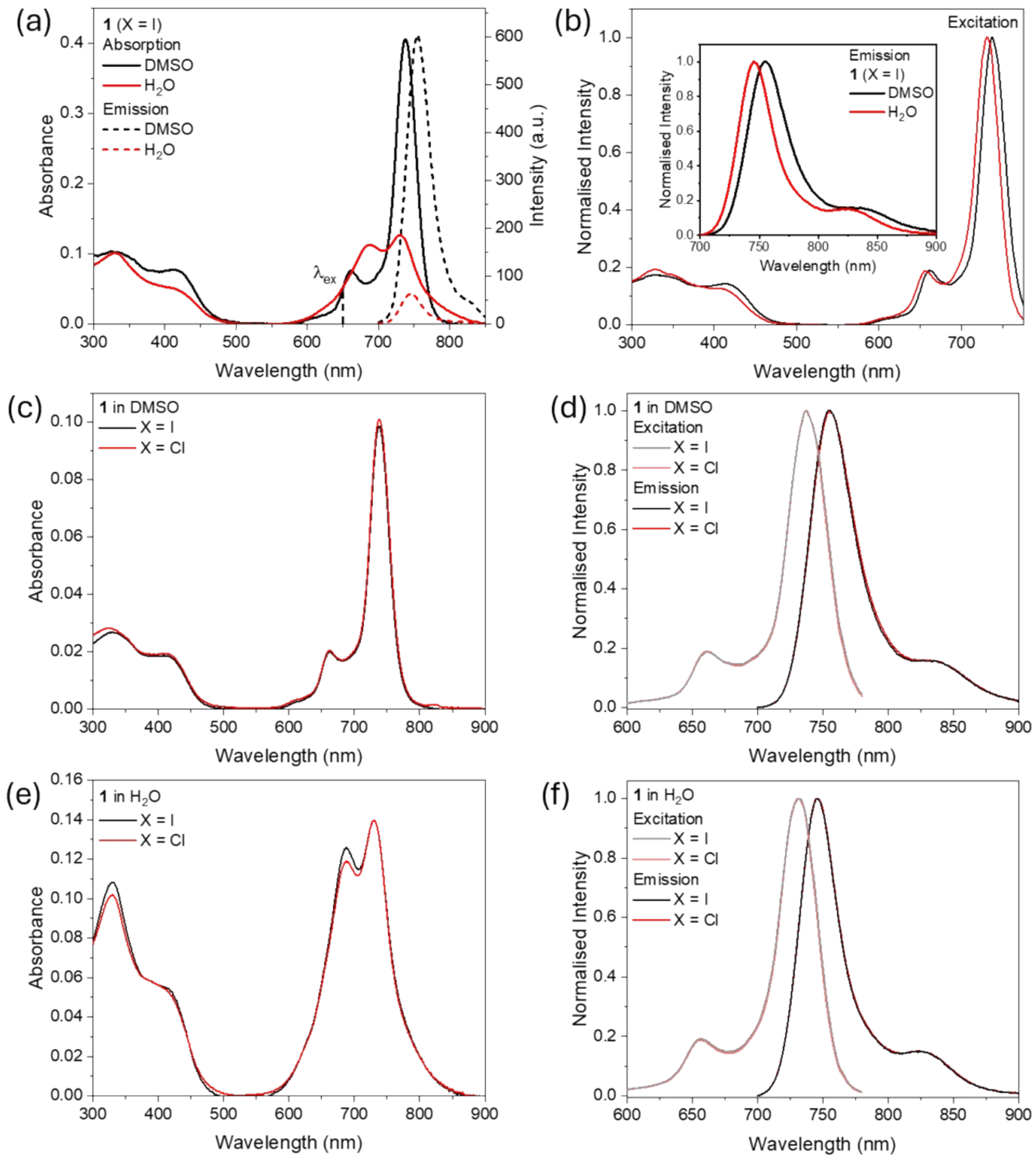

Figure S1. (a) UV-vis absorption (solid) and emission (dash) spectra and (b) normalised excitation and emission spectra (inset) of **1** (2.3 μM) in DMSO (black) and H_2O (red). λ_{ex} = 650 nm, λ_{em} = 825 nm. Inset of (c) shows emission intensity relative to intensity in DMSO against Abs_Q/Abs_A ratio.

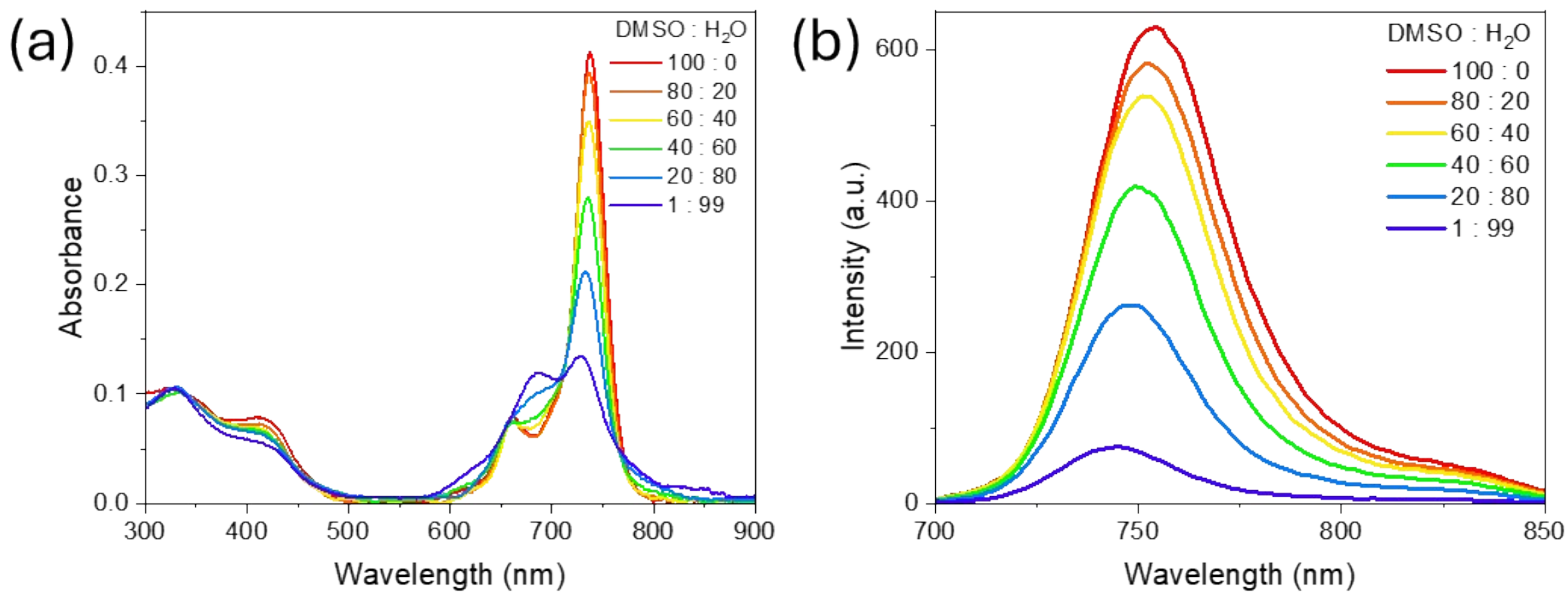

Figure S2. (a) UV-visible absorption and (b) emission (λ_{ex} = 650 nm) spectra of **1** (2.3 μM) in varying DMSO:H_2O ratios.

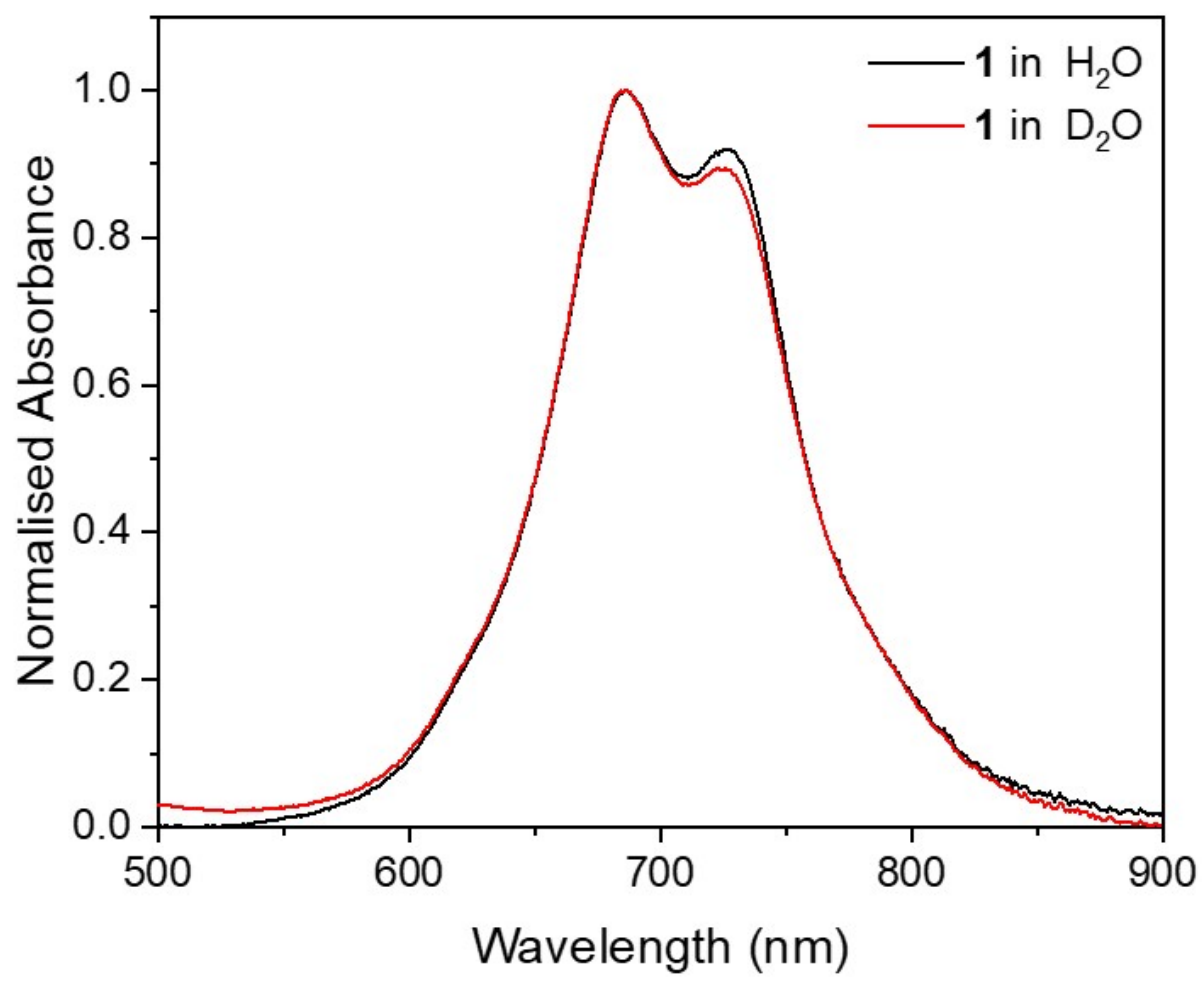

Figure S3. Normalised UV-visible absorption spectra of **1** in H_2O (35 μM) and D_2O (46 μM).

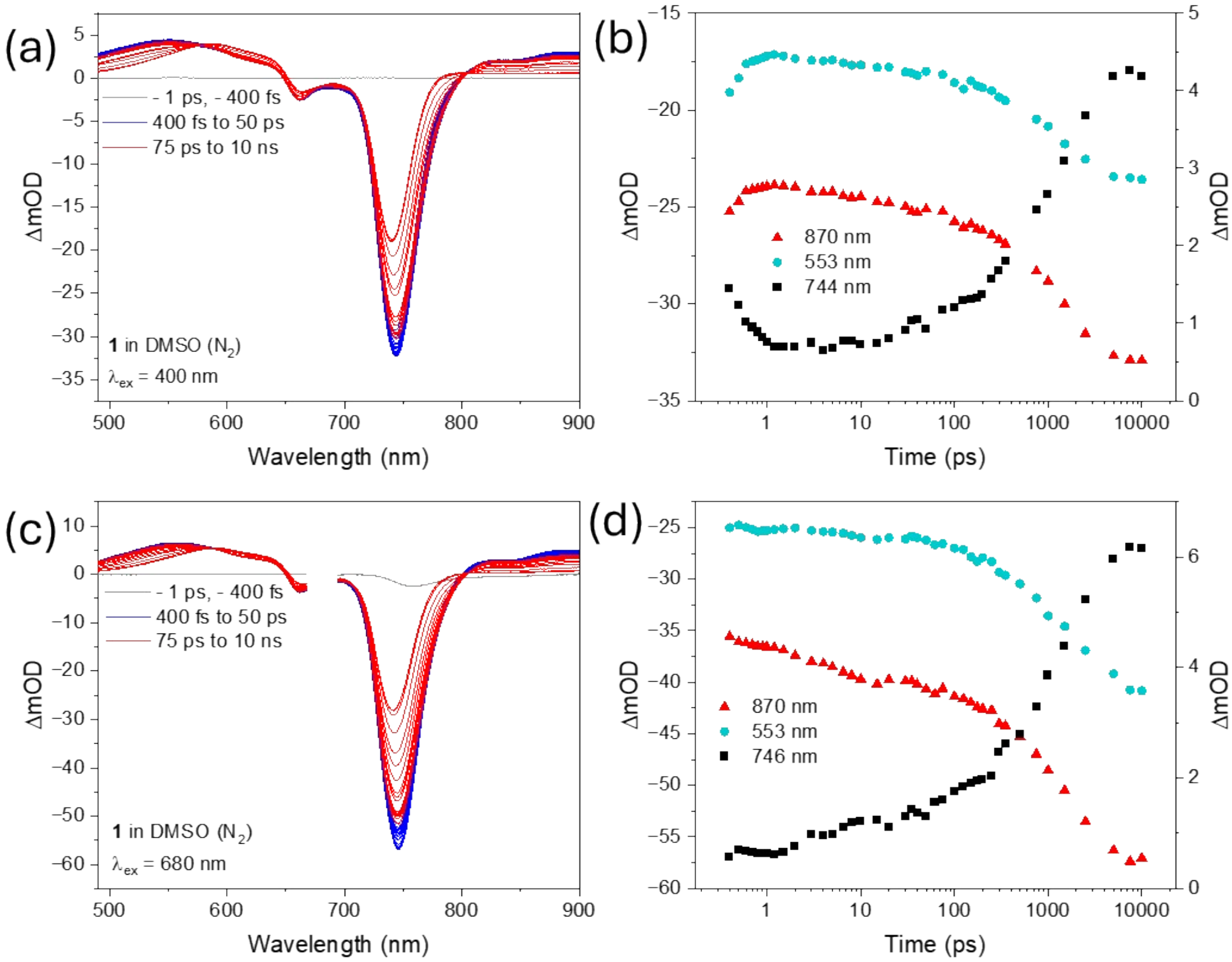

Figure S4. TA spectra of **1** (12 μM) in degassed DMSO at RT with (a) λ_{ex} = 400 nm and (c) λ_{ex} = 680 nm. ΔOD of bleach and transient bands between 400 fs and 10 ns on logarithmic time plot with (b) λ_{ex} = 400 nm and (d) λ_{ex} = 680 nm. Pump energy = 200 nJ

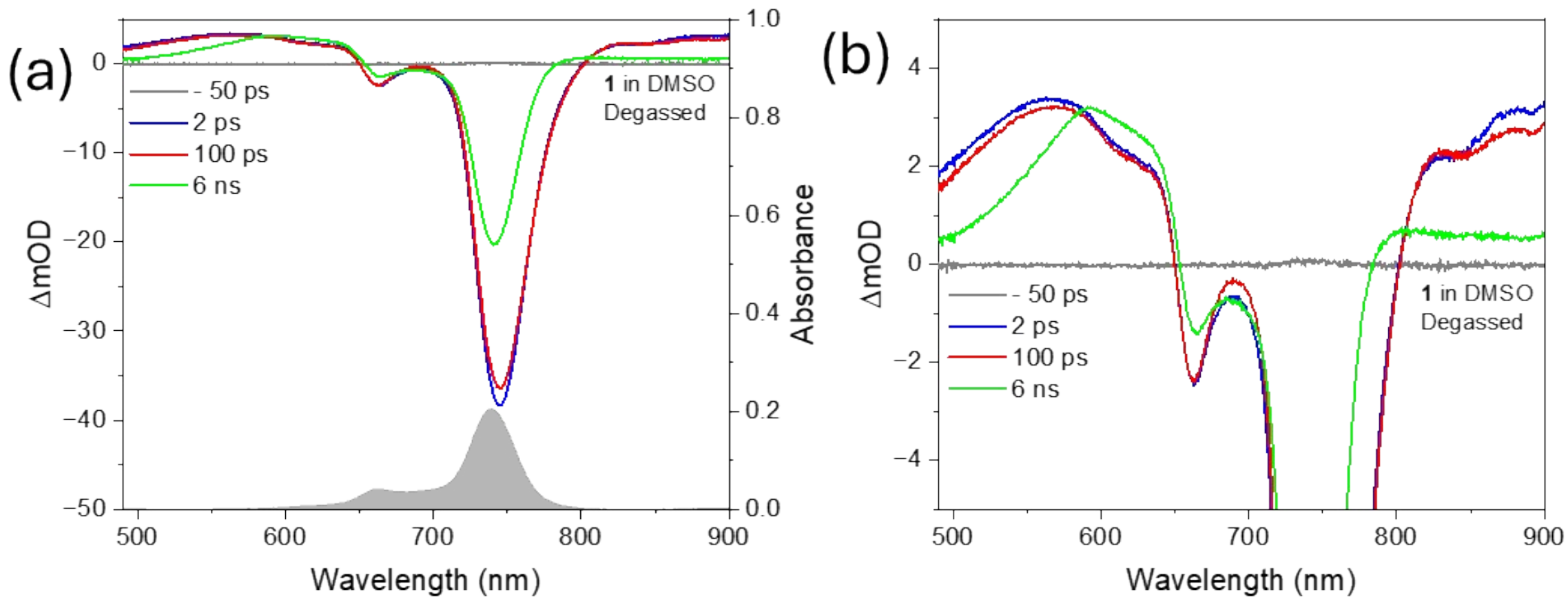

Figure S5. (a) TA and ground state absorption spectra of **1** (12 μM) in degassed DMSO. (b) Transient bands in TA spectra at 2 ps, 100 ps, and 6 ns. λ_{ex} = 400 nm, pump energy = 200 nJ.

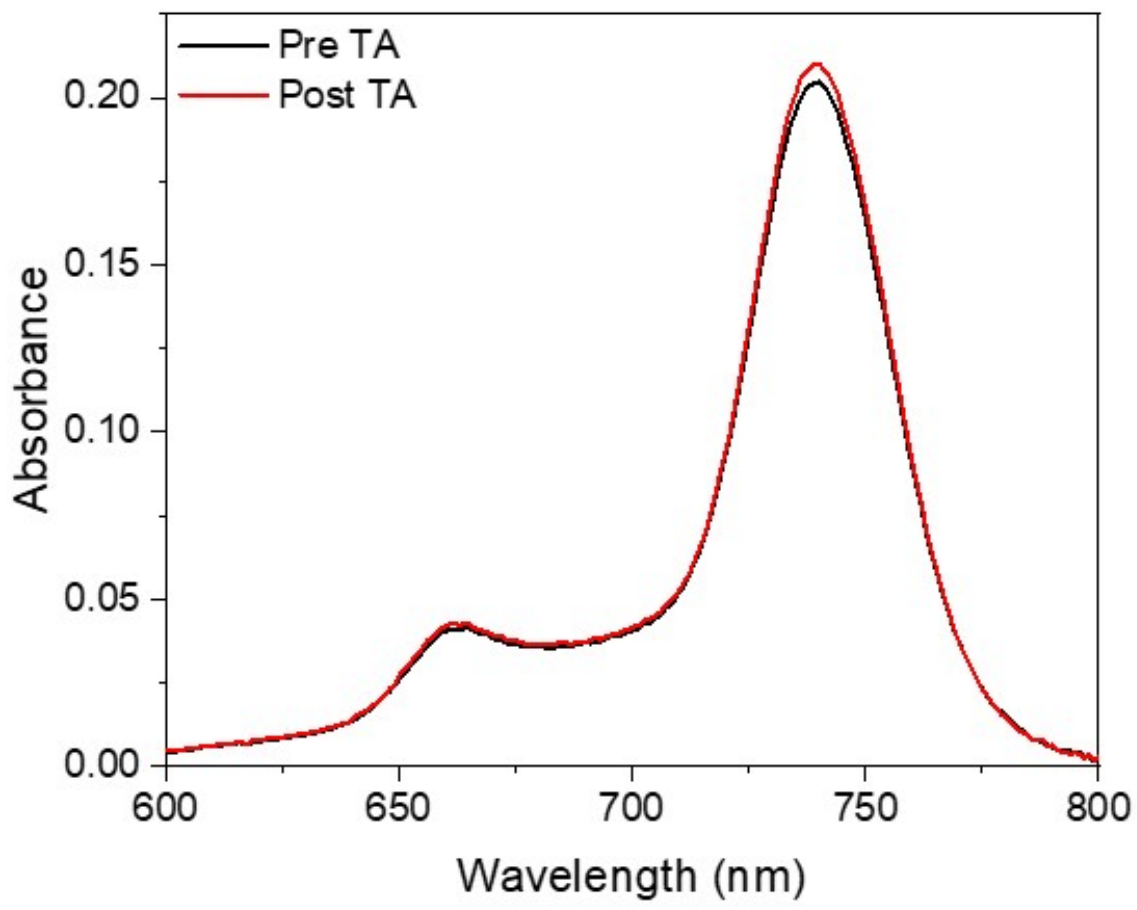

Figure S6. UV-visible absorption spectra of **1** (12 μM) in DMSO pre and post TA experiment (λ_{ex} = 400 nm, pump power = 200 nJ).

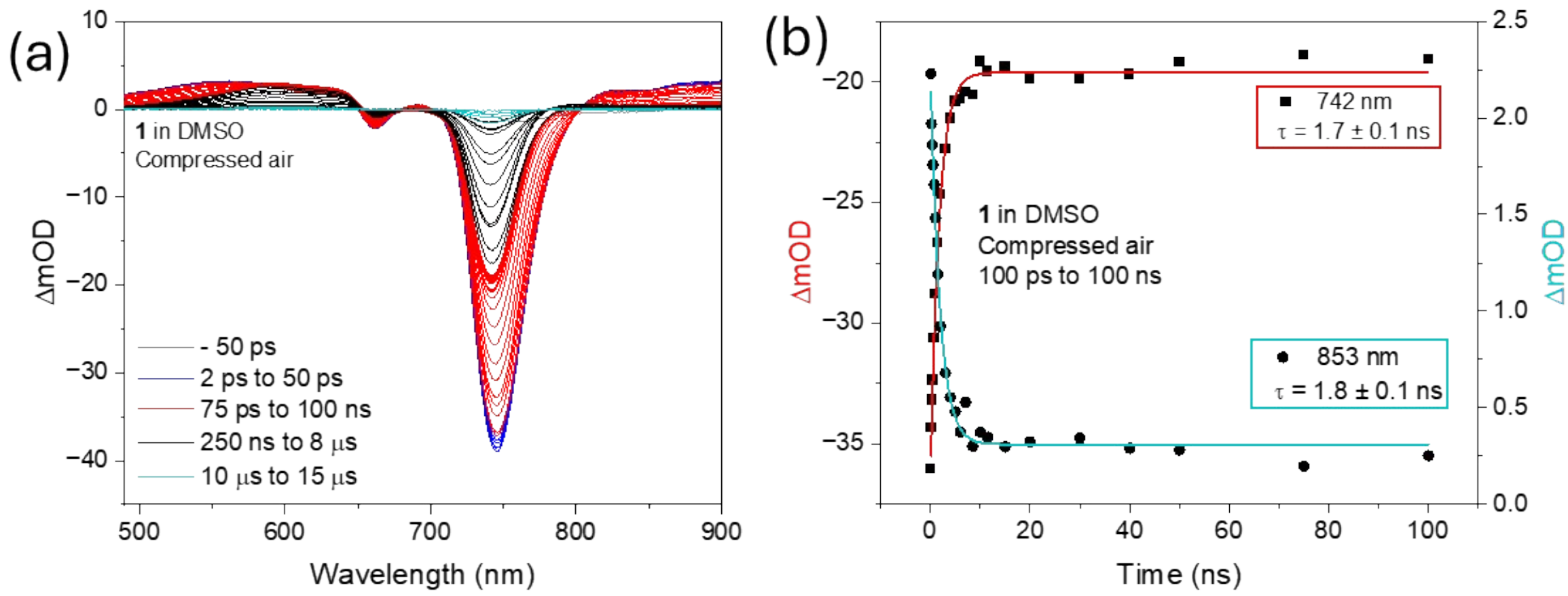

Figure S7. TA kinetics of **1** (12 μM) in DMSO at RT (λ_{ex} = 400 nm, pump energy = 200 nJ). Recovery of GSB at 742 nm and ESA at 608 nm following bubbling with (a) compressed air and (b) N_2 for 5 min between 100 ps and 20 ns. Solid lines show monoexponential fits.

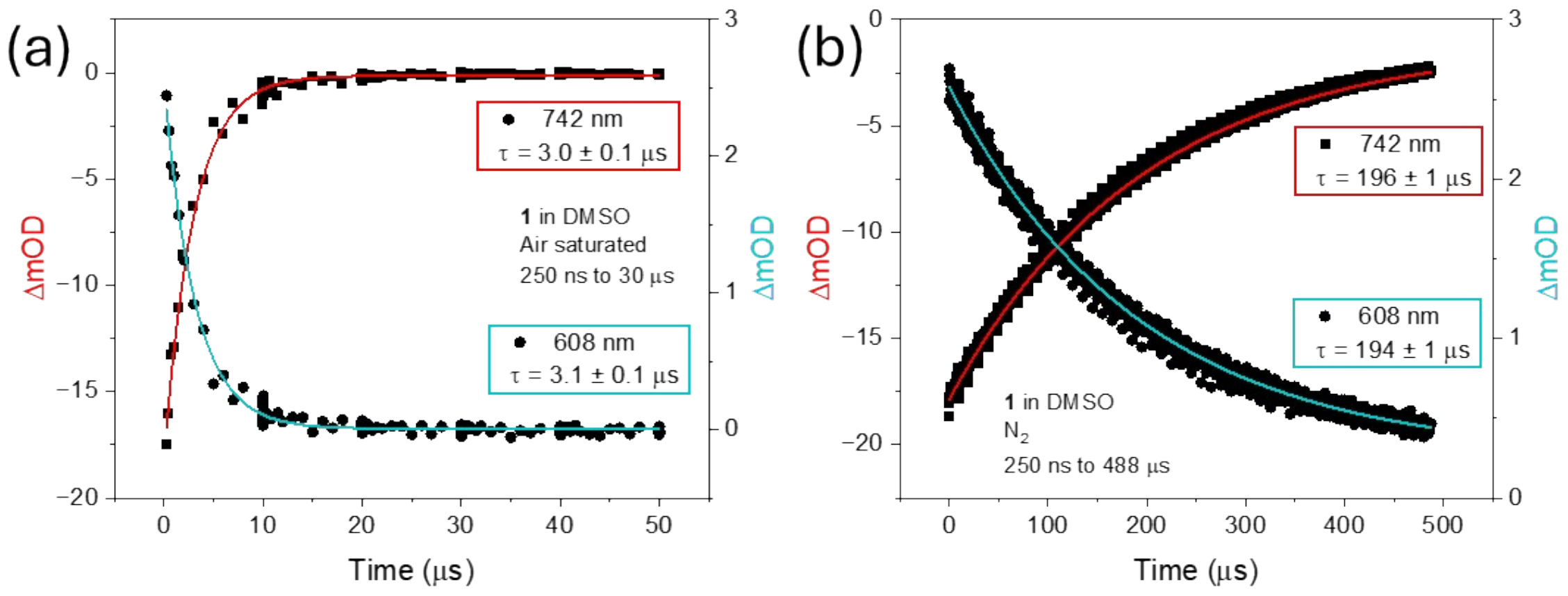

Figure S8. TA kinetics of **1** (12 μM) in DMSO at RT (λ_{ex} = 400 nm, pump energy = 200 nJ). Recovery of GSB at 742 nm and ESA at 608 nm following bubbling with (a) compressed air and (b) N_2 for 5 min between 250 ns and 30 μs. Solid lines show monoexponential fits.

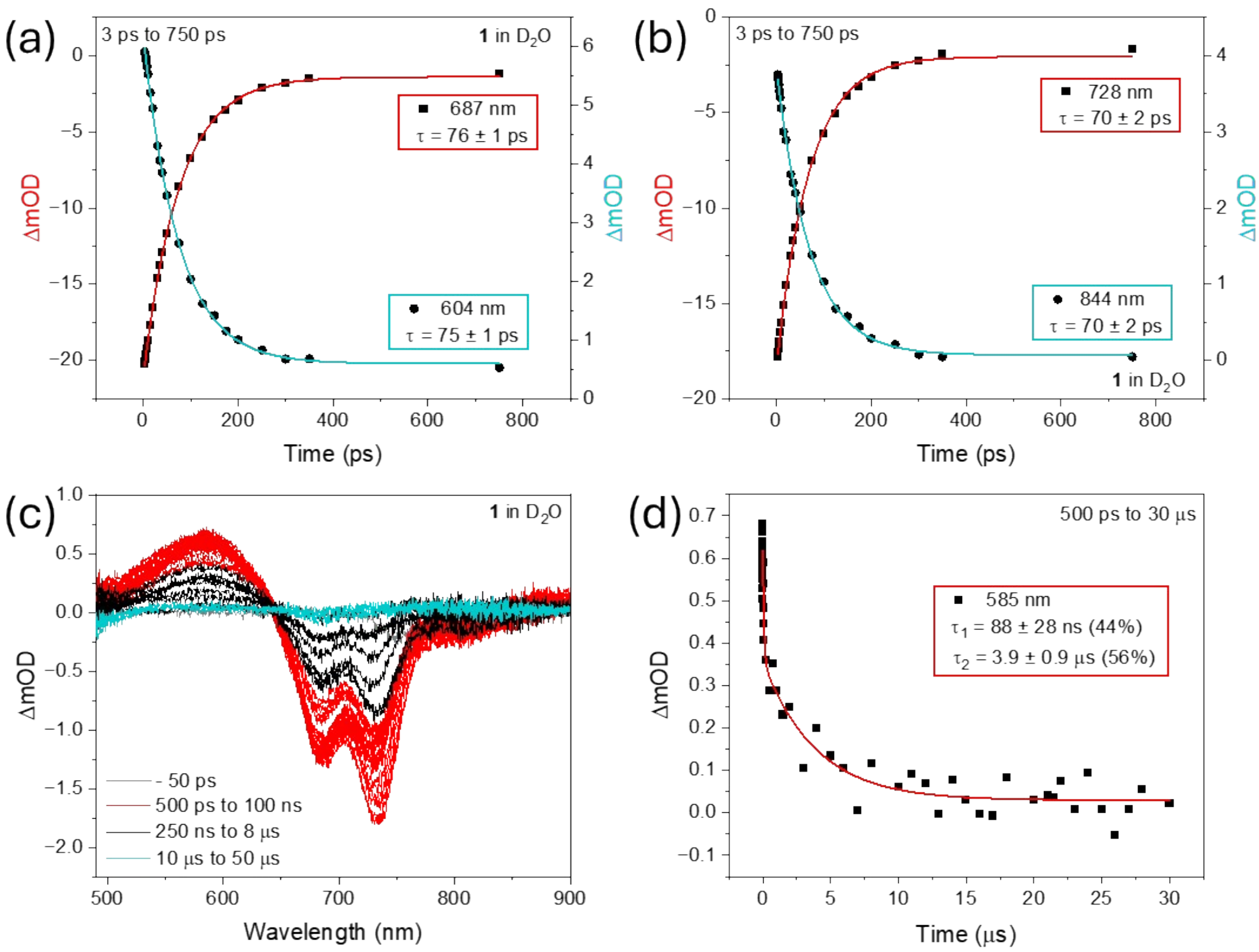

Figure S9. TA spectra and kinetics of **1** (23 μM) in D_2O (λ_{ex} = 400 nm, pump energy = 200 nJ). Recovery of (a) GSB at 687 nm and ESA at 604 nm and (b) GSB at 728 nm and ESA at 844 nm between 3 ps and 750 ns. (c) TA spectra between 500 ps and 50 μs. (d) Recovery of ESA at 585 nm between 500 ps and 30 μs. Solid lines show exponential fits.

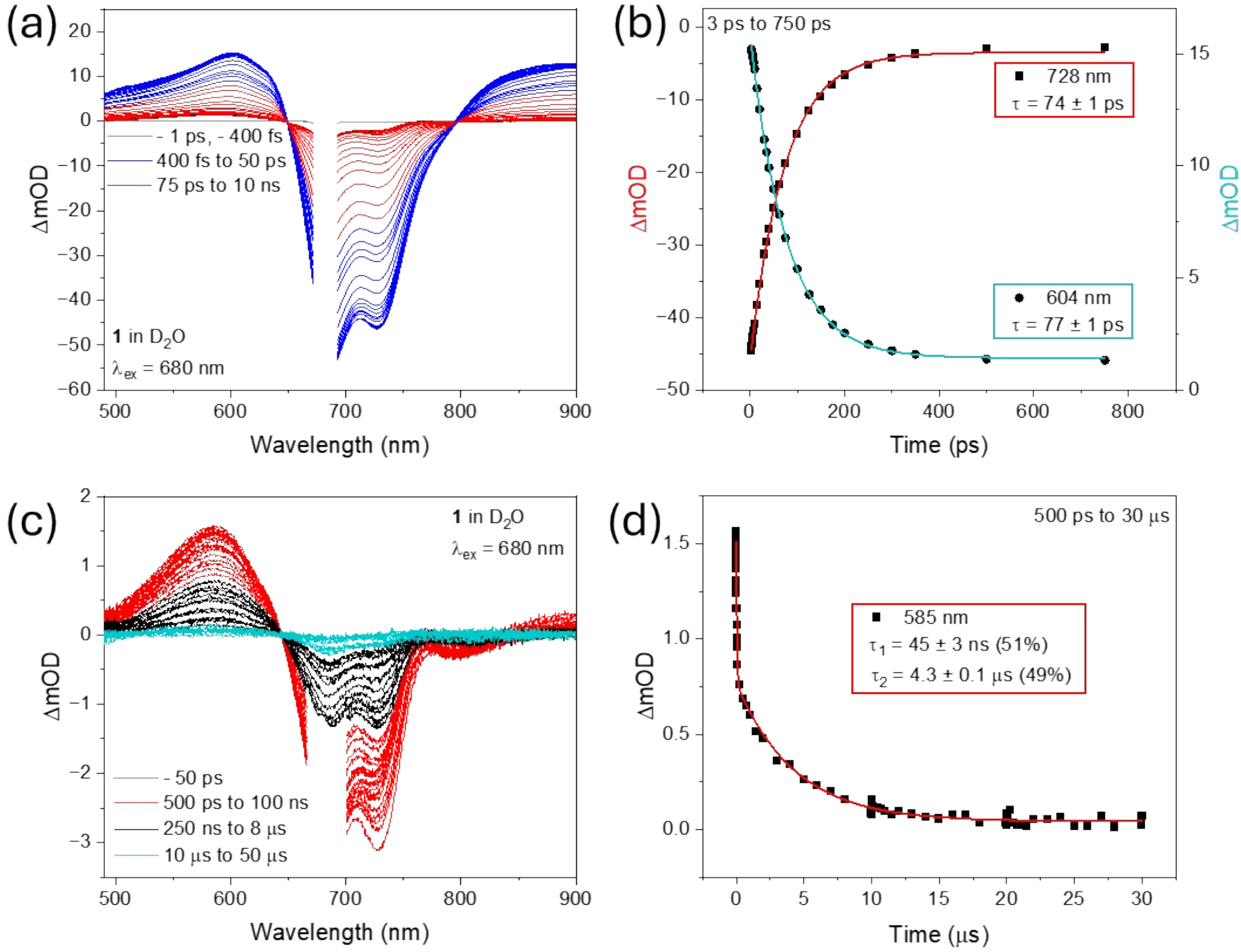

Figure S10. TA spectra and kinetics of recovery of **1** (23 µM) in D_2O (λ_{ex} = 680 nm, pump energy = 200 nJ). (a) TA spectra between 400 fs to 10 ns. (b) Recovery of GSB at 728 nm and ESA at 604 nm between 3 ps and 750 ps. (c) TA spectra between 500 ps and 50 µs. (d) Recovery of ESA at 585 nm between 500 ps to 50 µs. Solid lines show exponential fits.

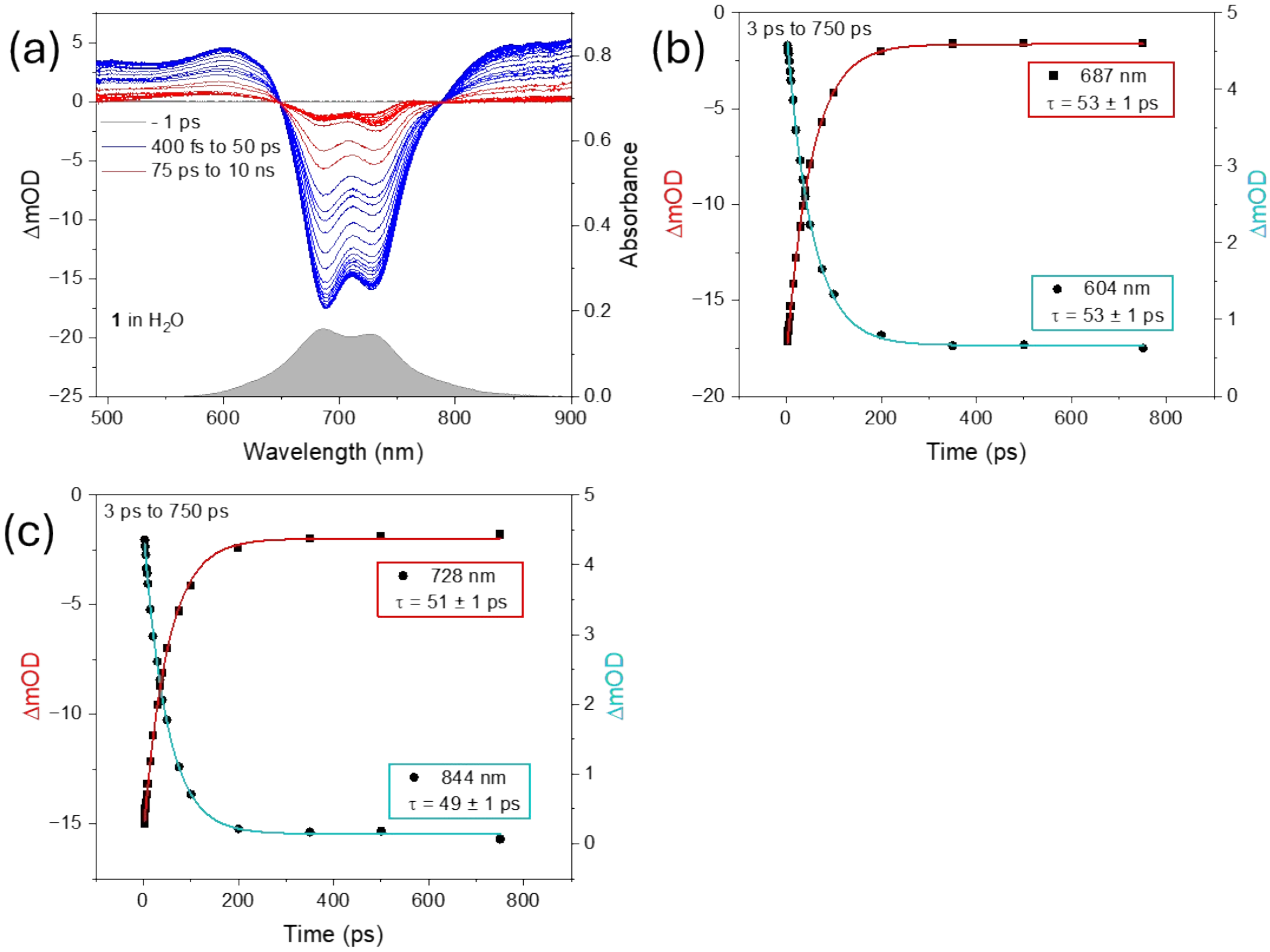

Figure S11. TA spectra and kinetics of **1** (35 μM) in H_2O at RT (λ_{ex} = 400 nm, pump power = 120 nJ). (a) TA spectra up to 10 ns (left axis) and ground state absorption spectrum (right axis). Recovery of (b) GSB at 687 nm ESA at 604 nm and (c) GSB at 728 nm and ESA at 804 nm between 3 ps and 750 ps. Solid lines show monoexponential fits.

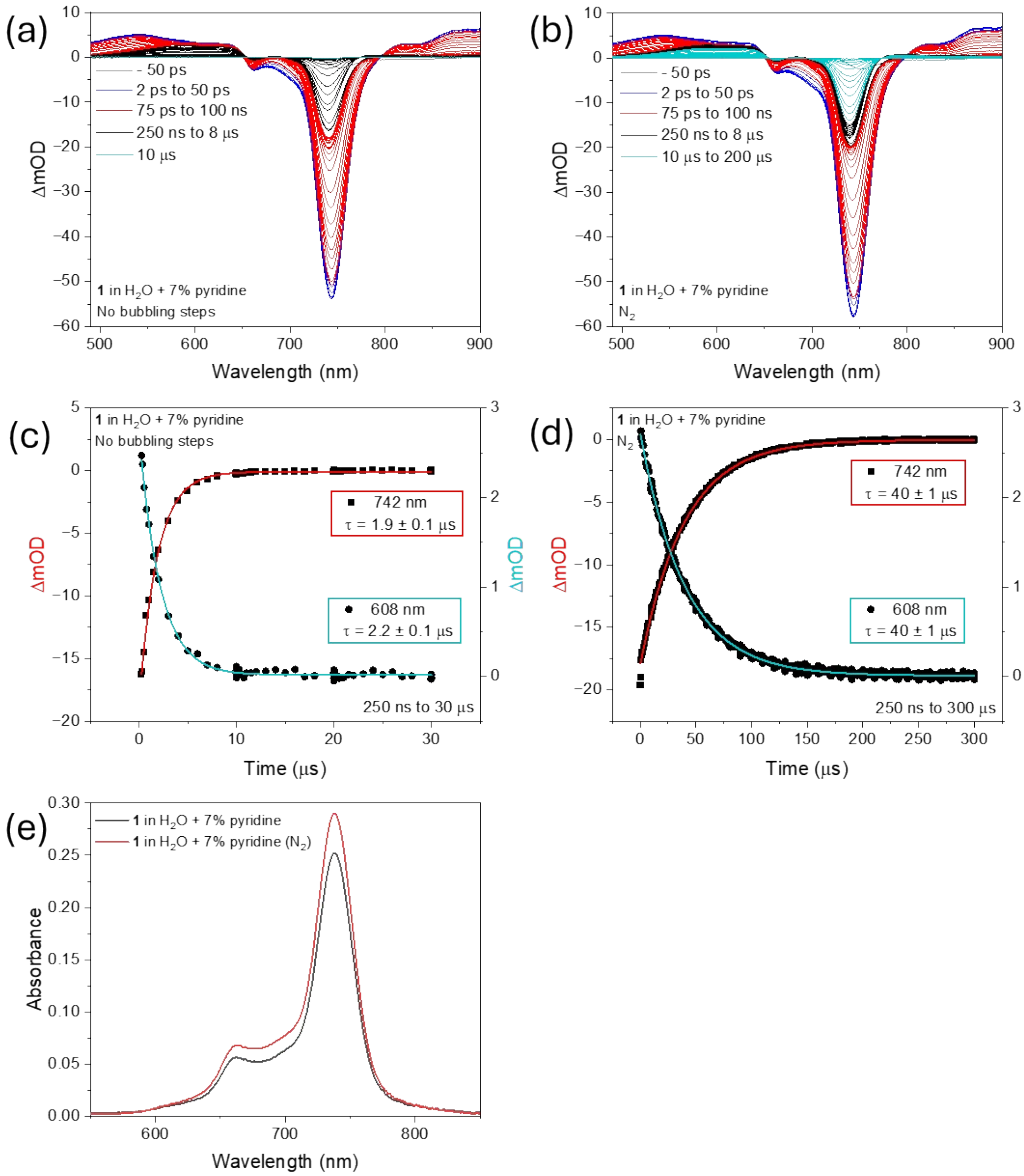

Figure S12. TA spectra and kinetics of **1** (30-35 μM) in H_2O with 7% pyridine (λ_{ex} = 400 nm, pump energy = 200 nJ. (a) TA spectra between 2 ps and 10 μs in air-saturated solution. (b) TA spectra between 2 ps and 200 μs following degassing with N_2 (N.B. degassing led to slight solvent loss, increasing concentration). Recovery of GSB at 742 nm and ESA at 608 nm for (c) air-saturated solution and (d) N_2 degassed solution. (e) Ground state absorption spectra of **1** in H_2O with 7% pyridine.

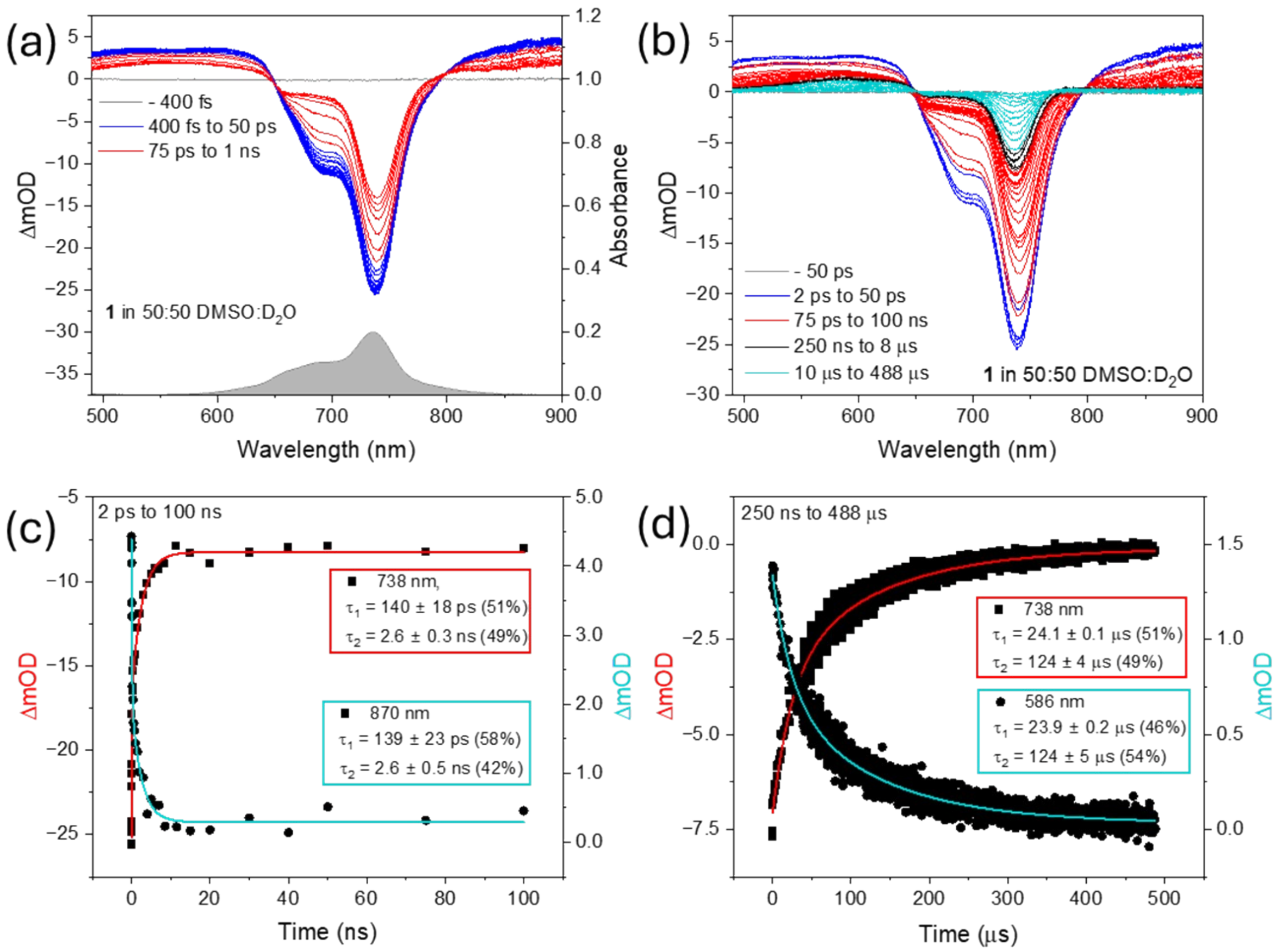

Figure S13. TA spectra and kinetics of **1** (23 μM) in 50:50 DMSO:D_2O (by volume) at RT (λ_{ex} = 400 nm, pump power = 200 nJ). (a) TA spectra up to 1 ns (left axis) and ground state absorption spectrum (right) axis. (b) TA spectra between 2 ps and 488 μs. (c) Recovery of GSB at 738 nm and ESA at 870 nm between 2 ps and 100 ns. (d) Recovery of GSB at 738 nm and ESA at 586 nm between 250 ns and 488 μs. Solid lines show exponential fits.

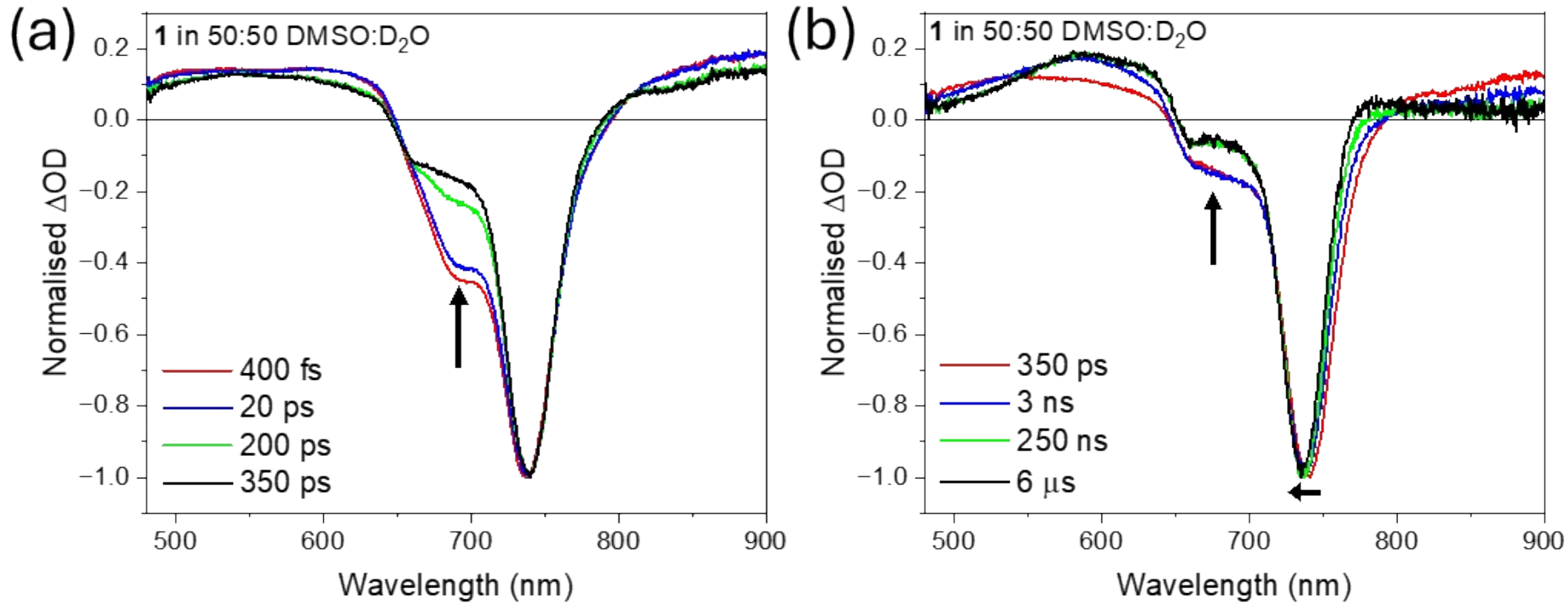

Figure S14. Normalised (at maximum GSB) TA spectra of **1** (23 μM) in 50:50 DMSO:D_2O (by volume) at RT (λ_{ex} = 400 nm, pump power = 200 nJ). between (a) 400 fs and 350 ps and (b) 350 ps and 6 μs.

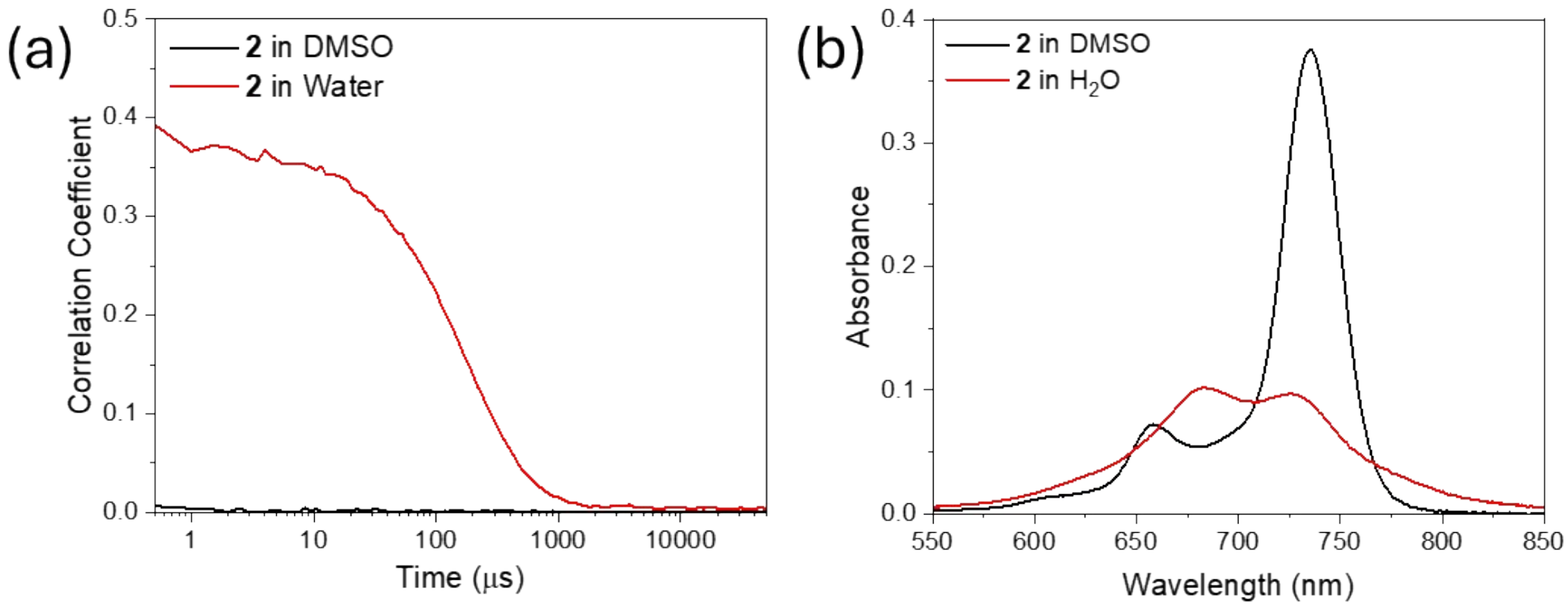

Figure S15. (a) Dynamic light scattering correlation coefficient against time for study of **2** (2.6 μM) aggregate formation in DMSO and H_2O at 25 °C. (b) Absorption spectra of **2** under same conditions.

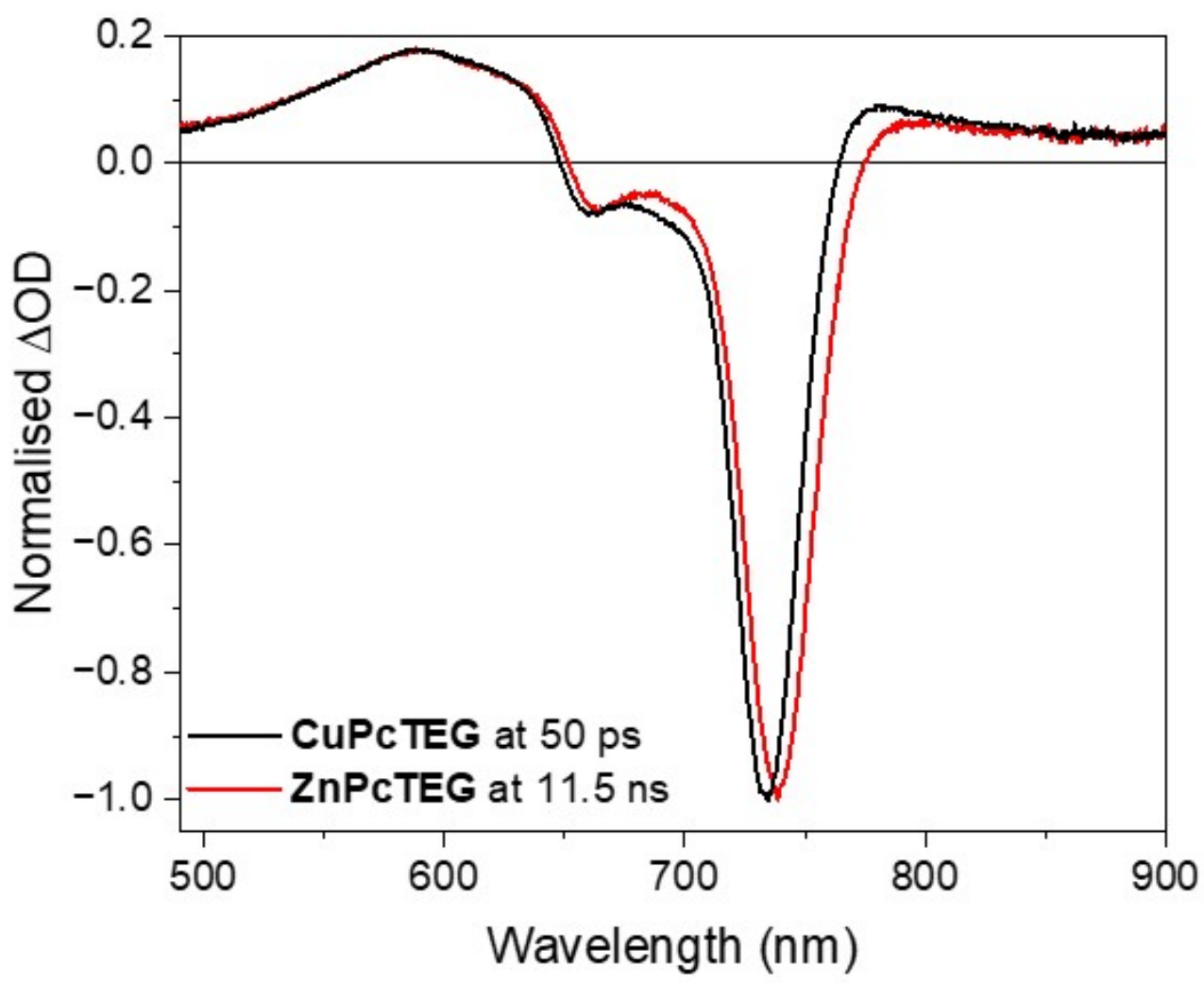

Figure S16. TA spectra normalised at GSB of **1** at 11.5 ns and **2** at 50 ps in DMSO.

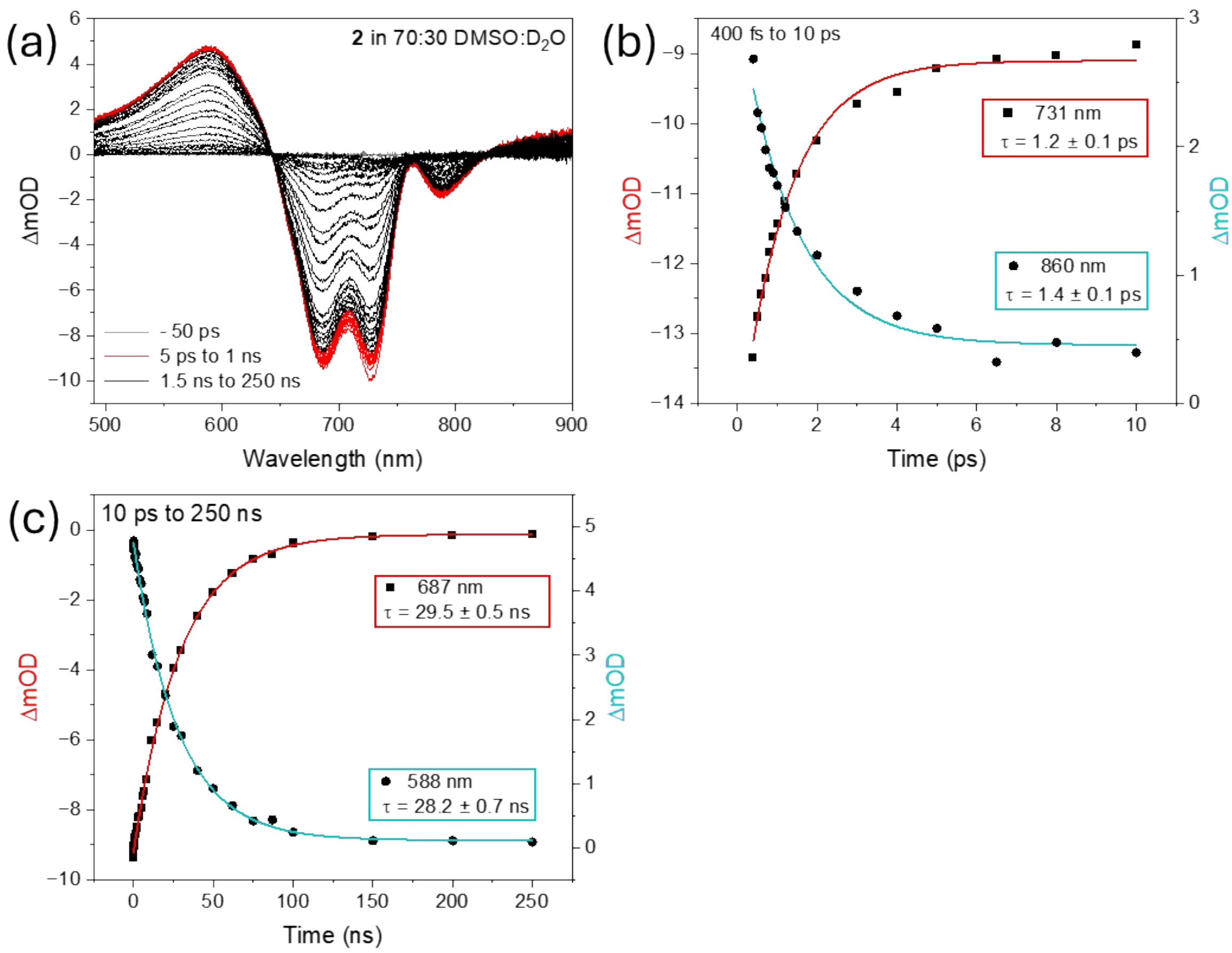

Figure S17. TA spectra and kinetics of **2** (24 μM) in 70:30 DMSO:D_2O by volume at RT (λ_{ex} = 400 nm, pump power = 200 nJ). (a) TA spectra between 5 ps and 250 ns. (b) Recovery of GSB at 731 nm and ESA at 860 nm between 400 fs and 10 ps. (c) Recovery of GSB at 687 nm and ESA at 588 nm between 10 ps and 250 ns. Solid lines show monoexponential fits. In

b the error on the mathematical fit is reported; for this short lifetime process, the uncertainty including the instrument response is approximately 0.2 ps, which is reflected in the reported values in Table 4.

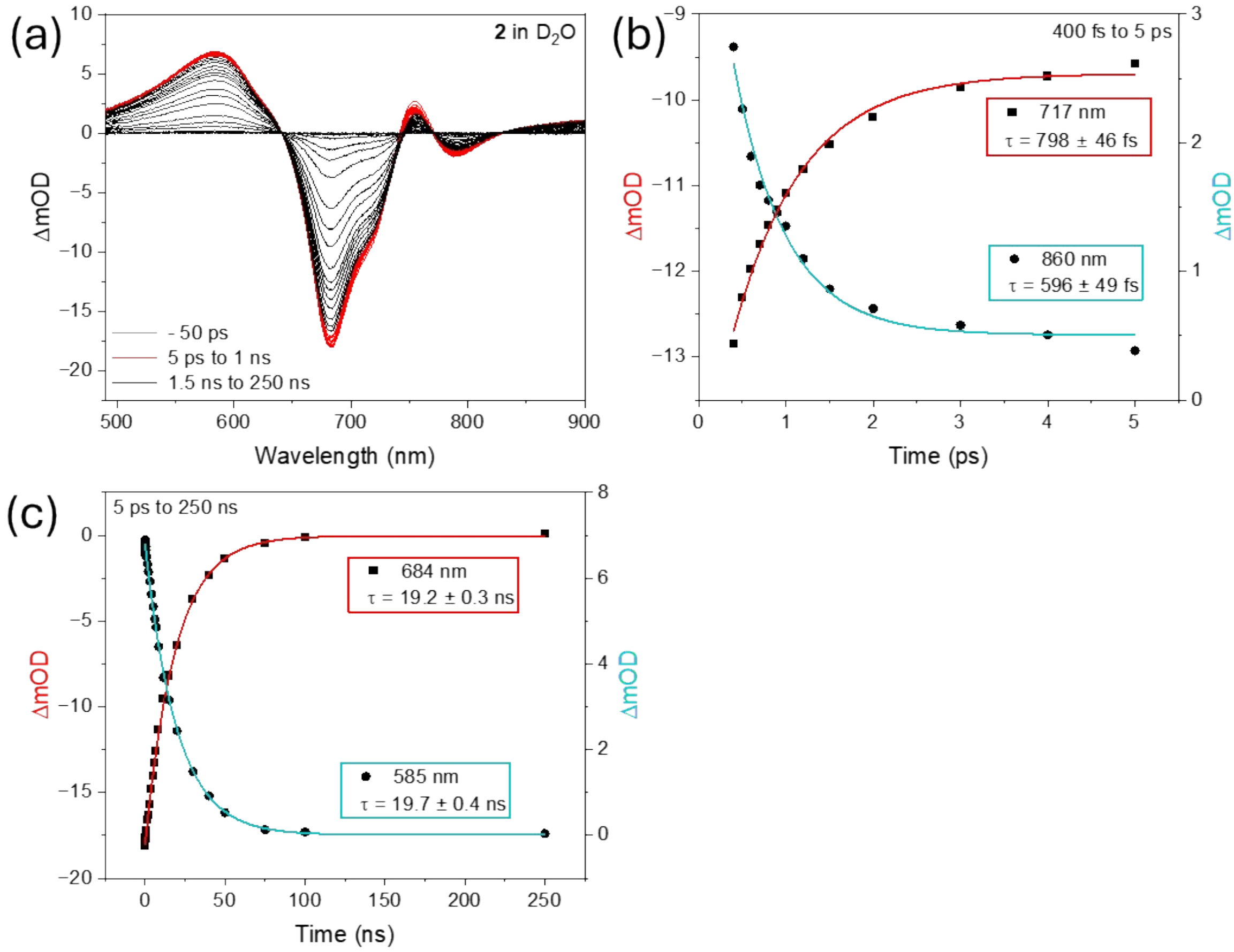

Figure S18. TA spectra and kinetics of **2** (48 μM) in D_2O at RT (λ_{ex} = 400 nm, pump power = 120 nJ). (a) TA spectra between 5 ps and 250 ns. (b) Recovery of GSB at 717 nm and ESA at 860 nm and (c) Recovery of GSB at 684 nm and ESA at 585 nm. Solid lines show monoexponential fits.

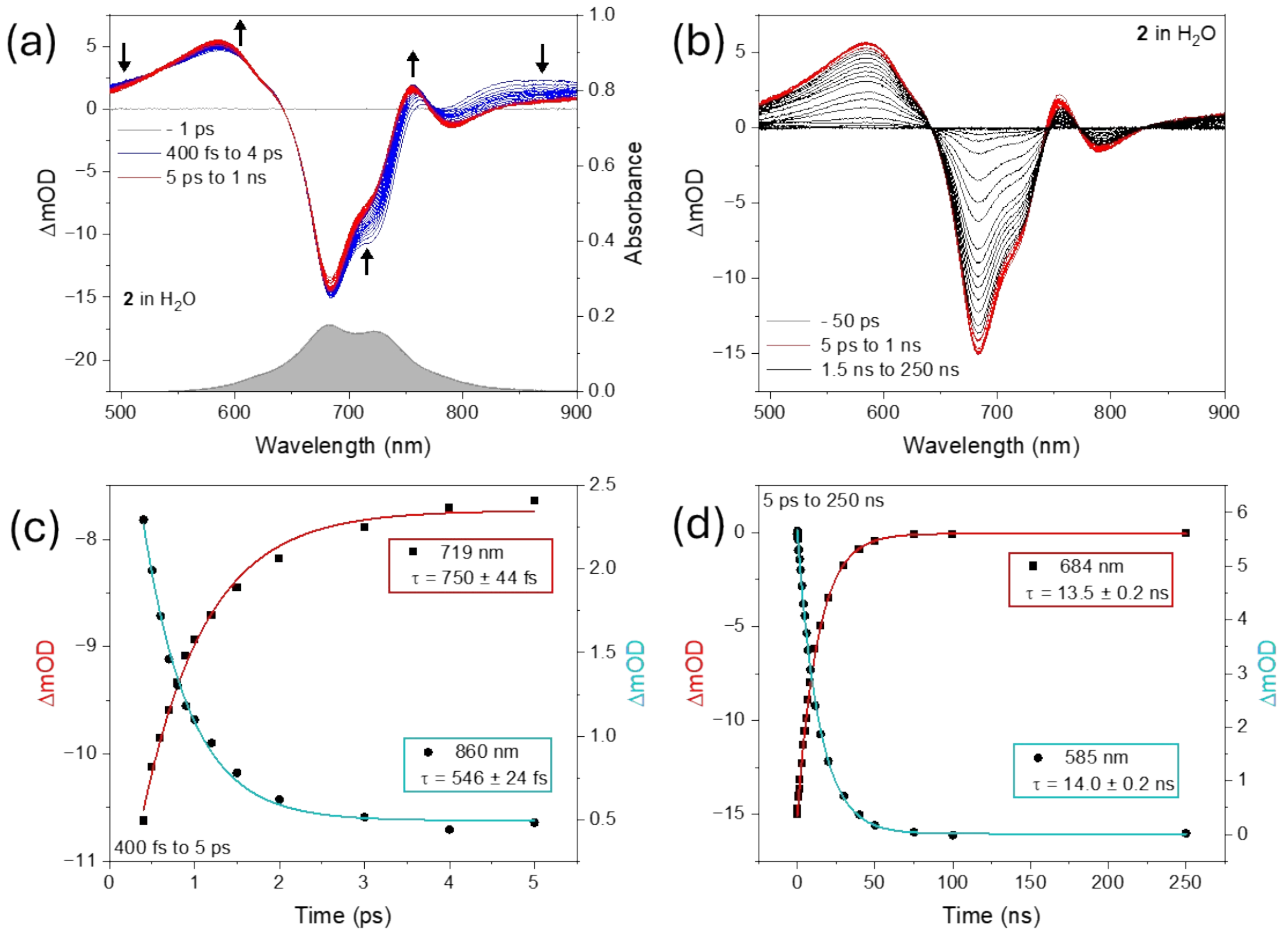

Figure S19. TA spectra and kinetics of **2** (30 μM) in H_2O from 400 fs at RT (λ_{ex} = 400 nm, pump power = 150 nJ). (a) TA spectra up to 1 ns (left axis) and ground state absorption spectrum (right axis). (b) TA spectra between 5 ps and 250 ns. (c) Recovery of GSB at 719 nm and ESA at 860 nm and (d) Recovery of GSB at 684 nm and recovery of ESA at 585 nm between 5 ps and 250 ns. Solid lines show monoexponential fits. In c the error on the mathematical fit is reported; for this short lifetime process, the uncertainty including the instrument response is approximately 0.2 ps, which is reflected in the reported values in Table 4.

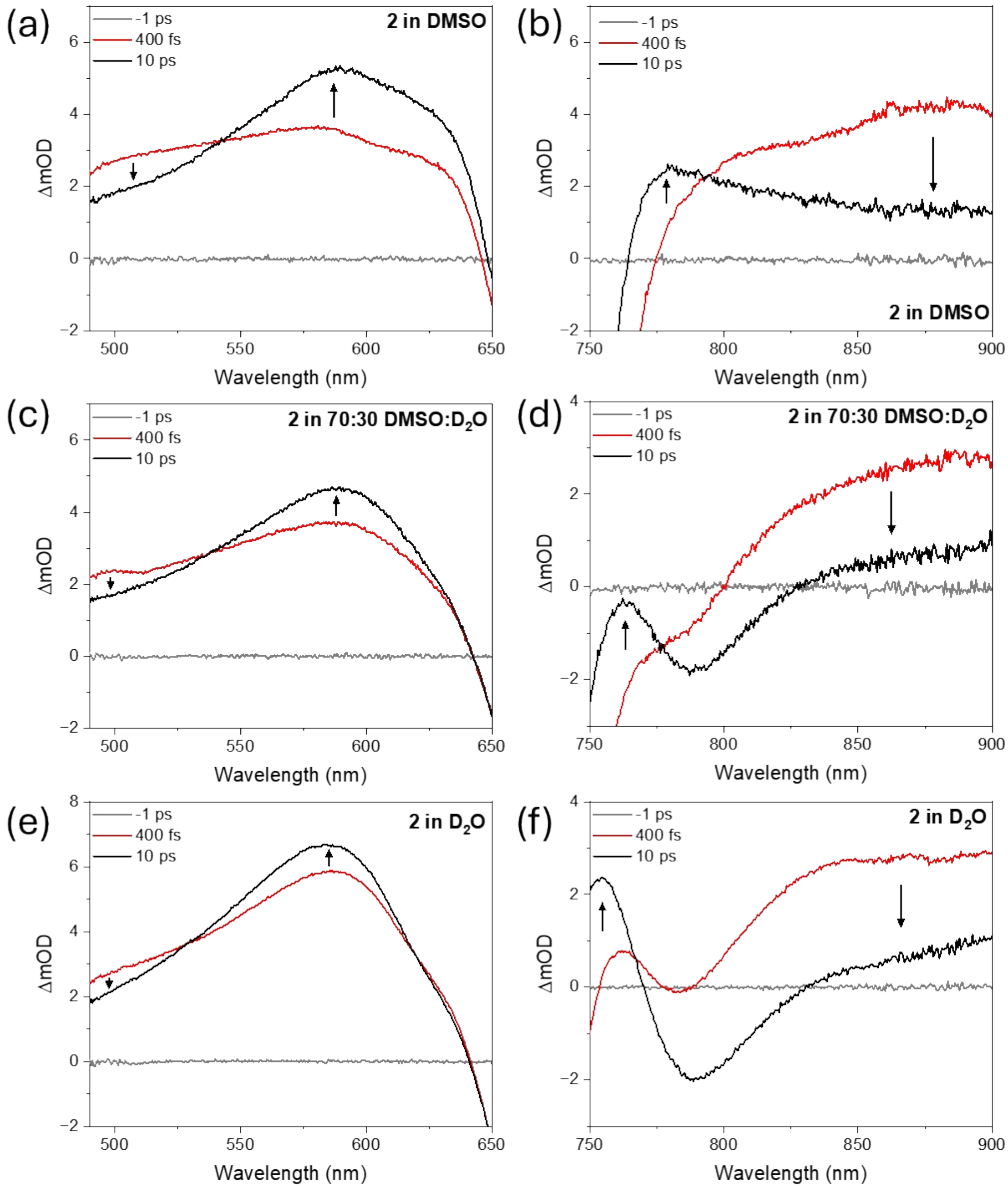

Figure S20. TA spectra of transient bands of **2** in (a, b) DMSO, (c, d) 70:30 DMSO:D_2O by volume, and (e, f) D_2O at 400 fs and 10 ps.

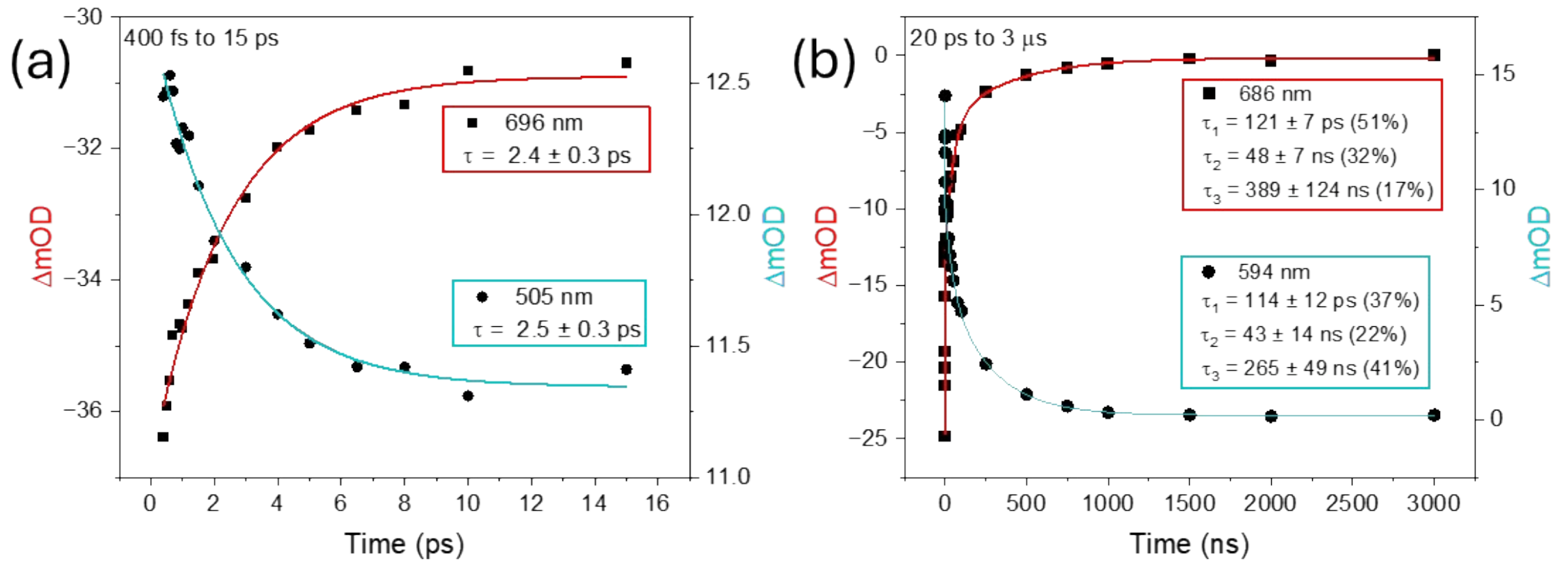

Figure S21. TA kinetics of **1** (iodide salt, 60 μM) in MeCN (λ_{ex} = 400 nm, pump power = 200 nJ). (a) Recovery of GSB at 696 nm (left axis) and ESA at 505 nm (right axis) between 400 fs and 15 ps. (b) Recovery of GSB at 686 nm (left axis) and ESA at 594 nm (right axis) between 20 ps and 3 μs. Solid lines show exponential fits.

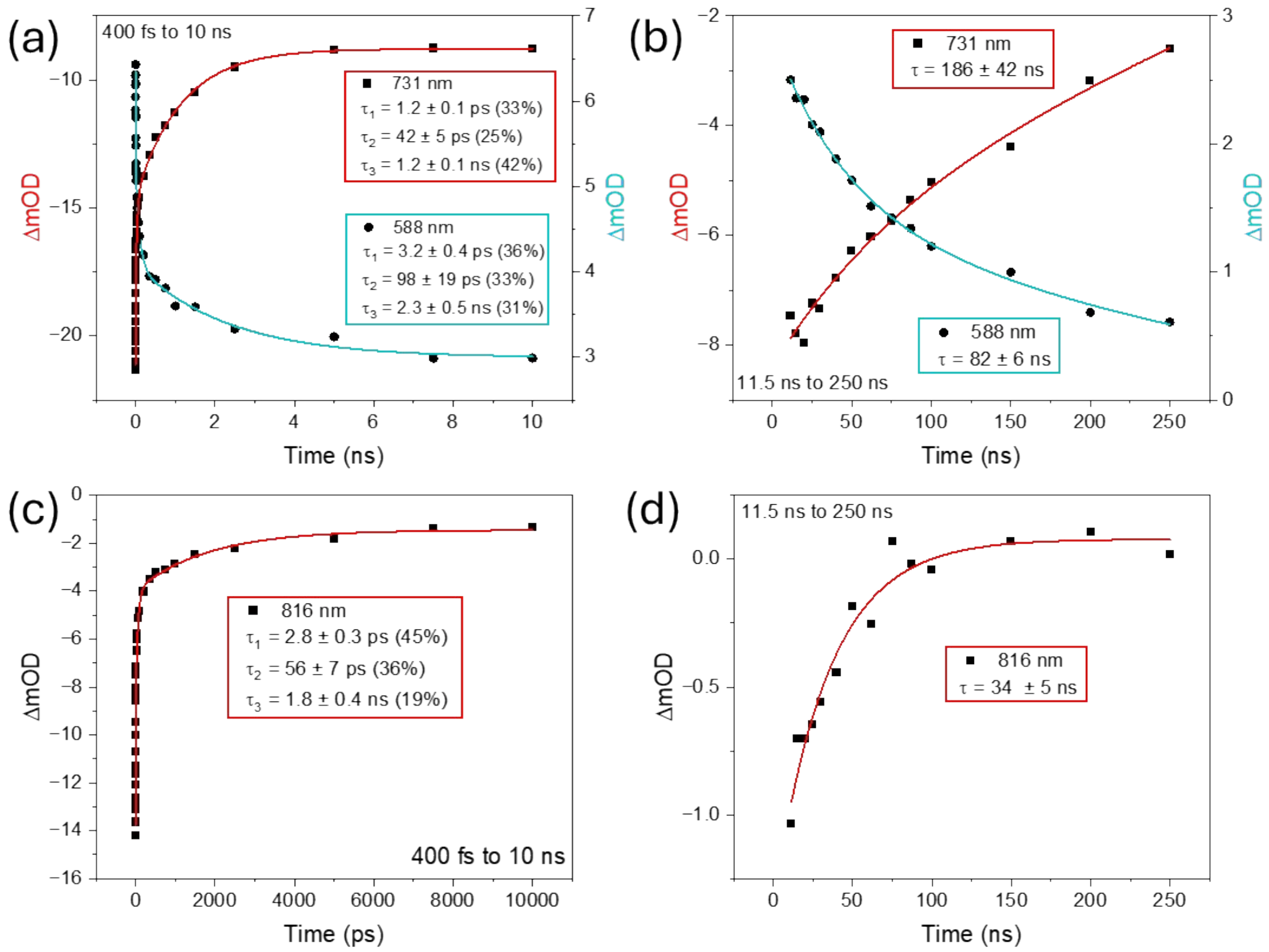

Figure S22. TA kinetics of **2** (iodide salt, 24 μM) in MeCN at RT (λ_{ex} = 400 nm, pump power = 200 nJ). Recovery of (a) GSB at 731 nm and ESA at 588 nm and (c) GSB at 816 nm between 400 fs and 10 ns. Recovery of (b) GSB at 731 nm and ESA at 588 nm and (d) GSB at 816 nm between 11.5 ns and 250 ns. Solid lines show exponential fits. In a, the error on the

mathematical fit is reported; for this short lifetime process, the uncertainty including the instrument response is approximately 0.2 ps, which is reflected in the reported values in Table 4.

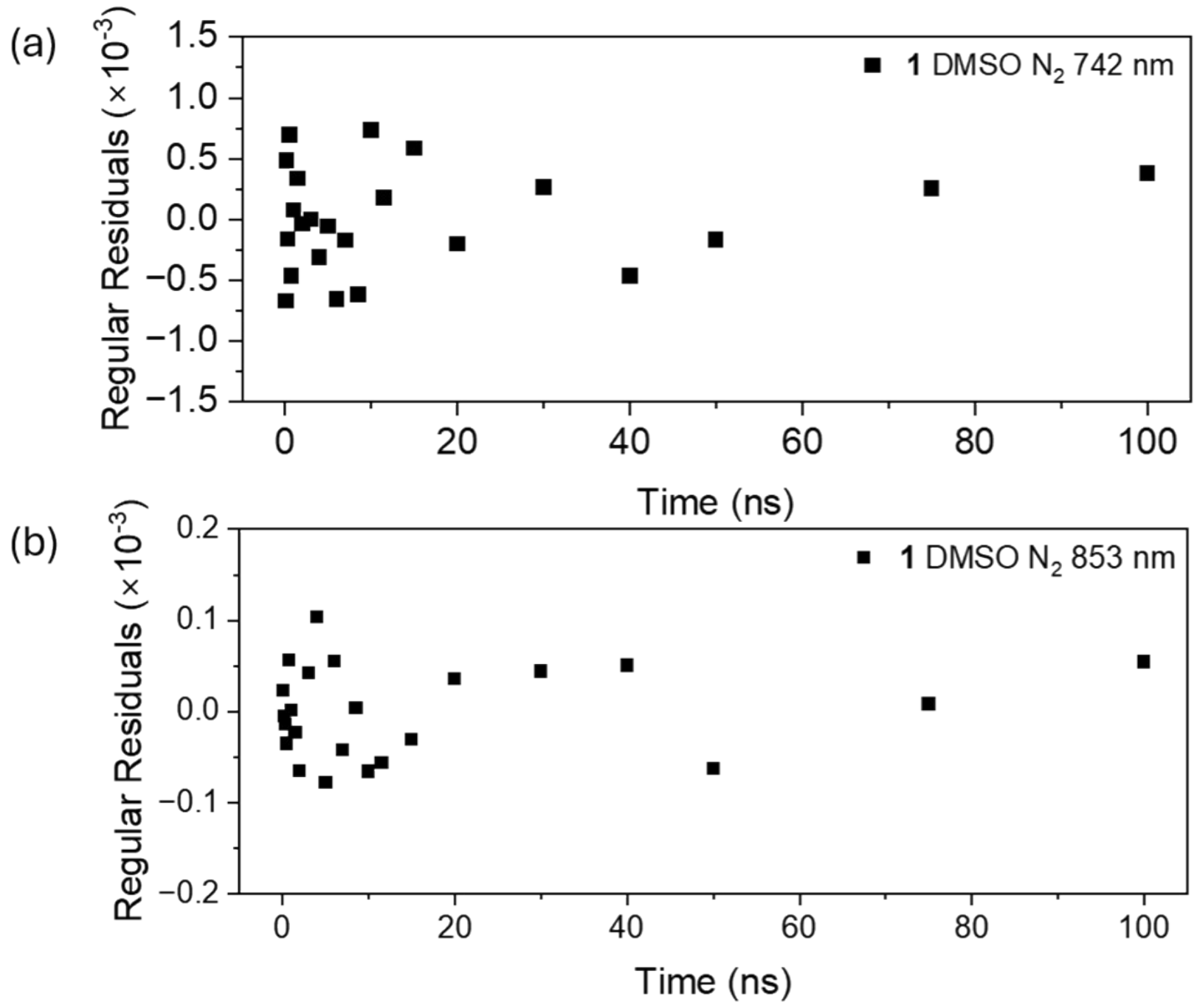

(a)

Equation	y = A1*exp(-x/t1) + y0
y0	-0.01916 ± 1.29209E-4
A1	-0.01682 ± 3.25151E-4
t1	1.59812 ± 0.07787
Reduced Chi-Sqr	1.9715E-7
Adj. R-Square	0.99381

(b)

Equation	y = A1*exp(-x/t1) + y0
y0	5.46702E-4 ± 1.54167E-5
A1	0.00188 ± 3.73678E-5
t1	1.69958 ± 0.08664
Reduced Chi-Sqr	2.73163E-9
Adj. R-Square	0.99328

Figure S23. Residuals and fit details of Fig 3c.

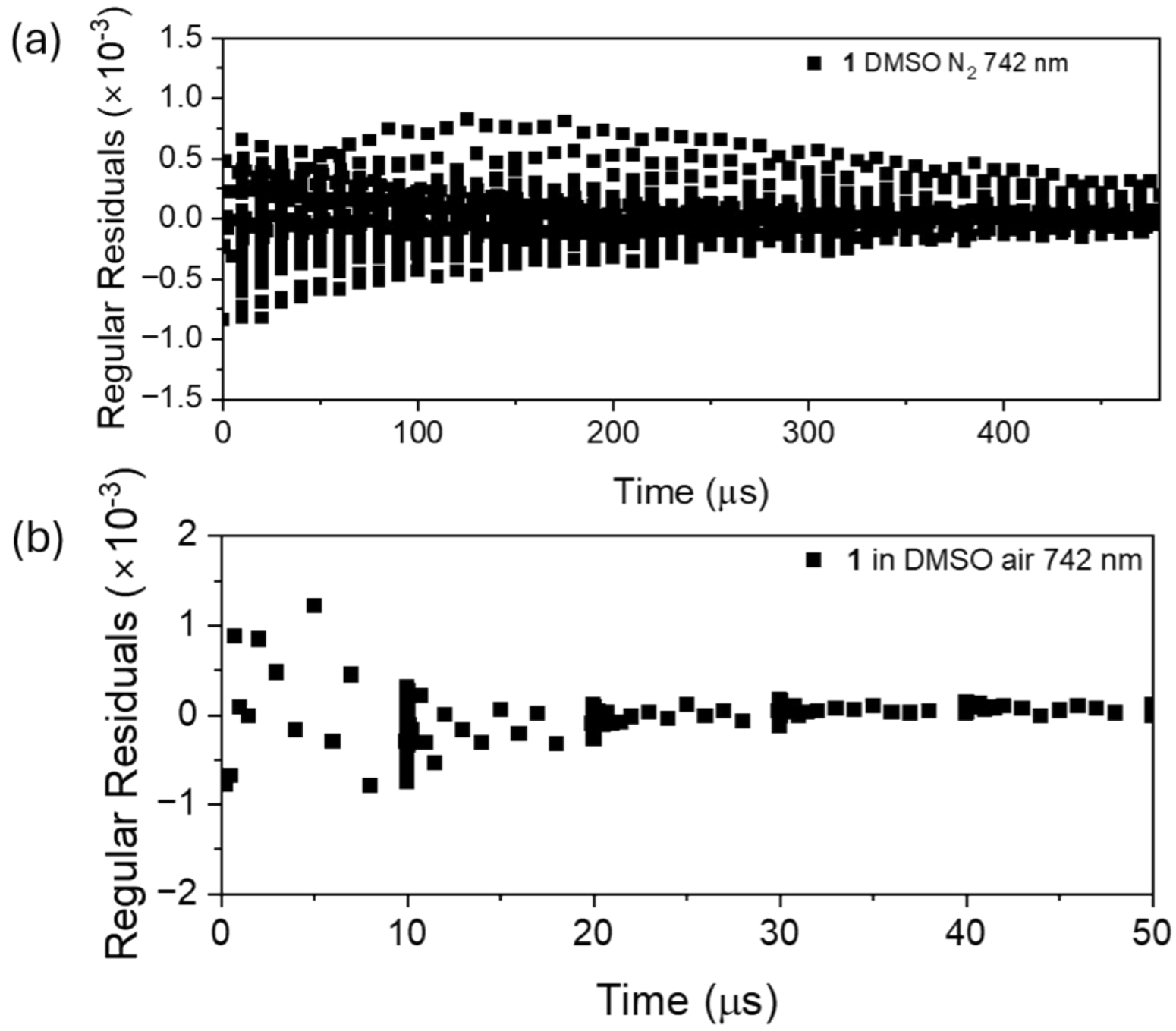

(a)

Equation	y = A1*exp(-x/t1) + y0
y0	-0.00108 ± 1.87595E-5
A1	-0.0168 ± 1.61233E-5
t1	196402.01907 ± 614.663
Reduced Chi-Sq	3.04399E-8
Adj. R-Square	0.99825

(b)

Equation	y = A1*exp(-x/t1) + y0
y0	-1.38301E-4 ± 1.85453E-5
A1	-0.018 ± 1.56109E-4
t1	3002.24313 ± 46.17991
Reduced Chi-Sqr	5.47626E-8
Adj. R-Square	0.98973

Figure S24. Residuals and fit details of Fig 3d.

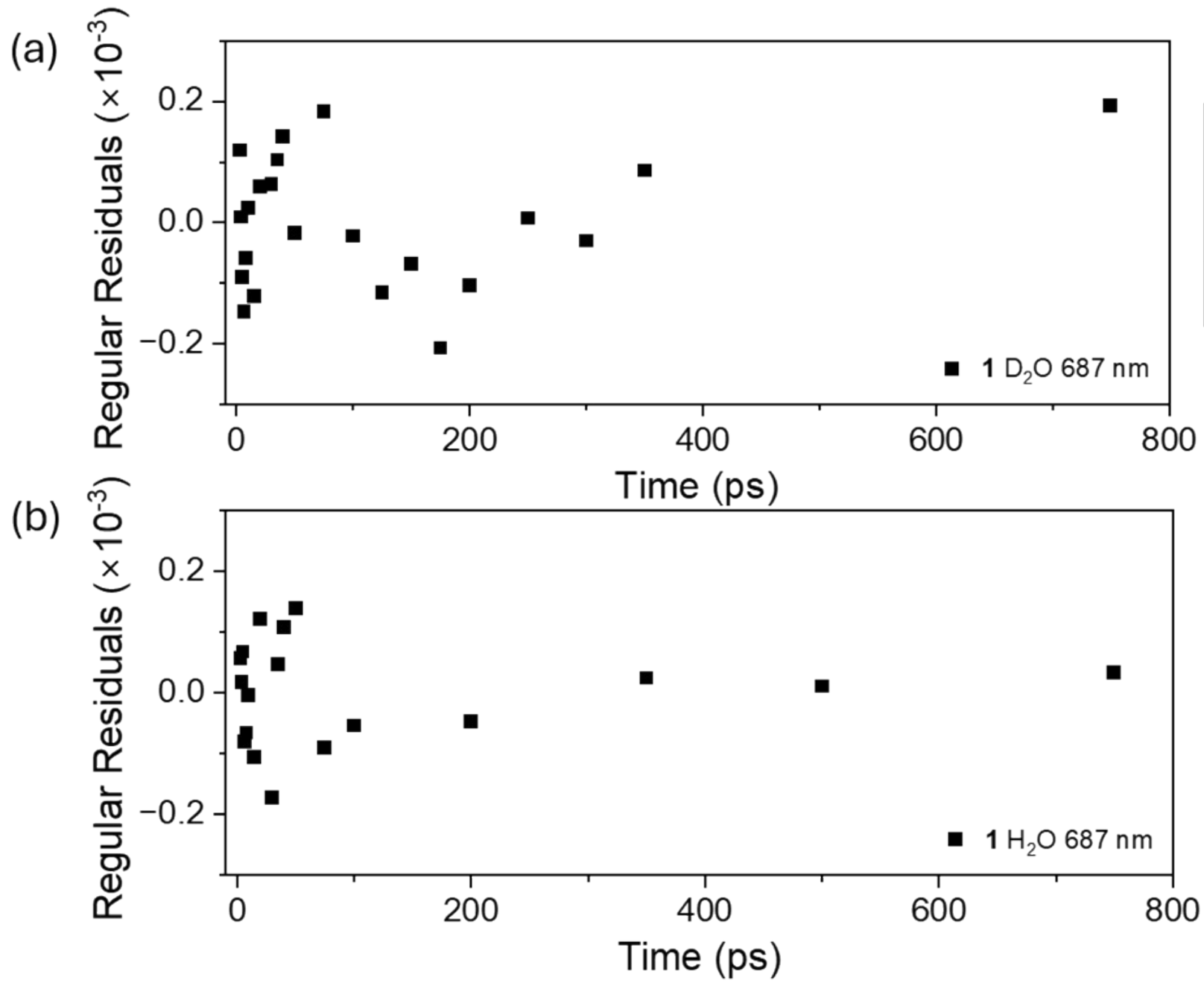

Equation	y = A1*exp(-x/t1) + y0
y0	-0.0014 ± 6.12715E-5
A1	-0.01972 ± 6.90799E-
t1	76.27402 ± 0.83365
Reduced Chi-Sqr	1.33004E-8
Adj. R-Square	0.99975

Equation	y = A1*exp(-x/t1) + y0
y0	-0.00163 ± 4.64267E-5
A1	-0.01651 ± 6.08705E-5
t1	52.85468 ± 0.52518
Reduced Chi-Sqr	8.17244E-9
Adj. R-Square	0.99977

Figure S25. Residuals and fit details of Fig. 4b.

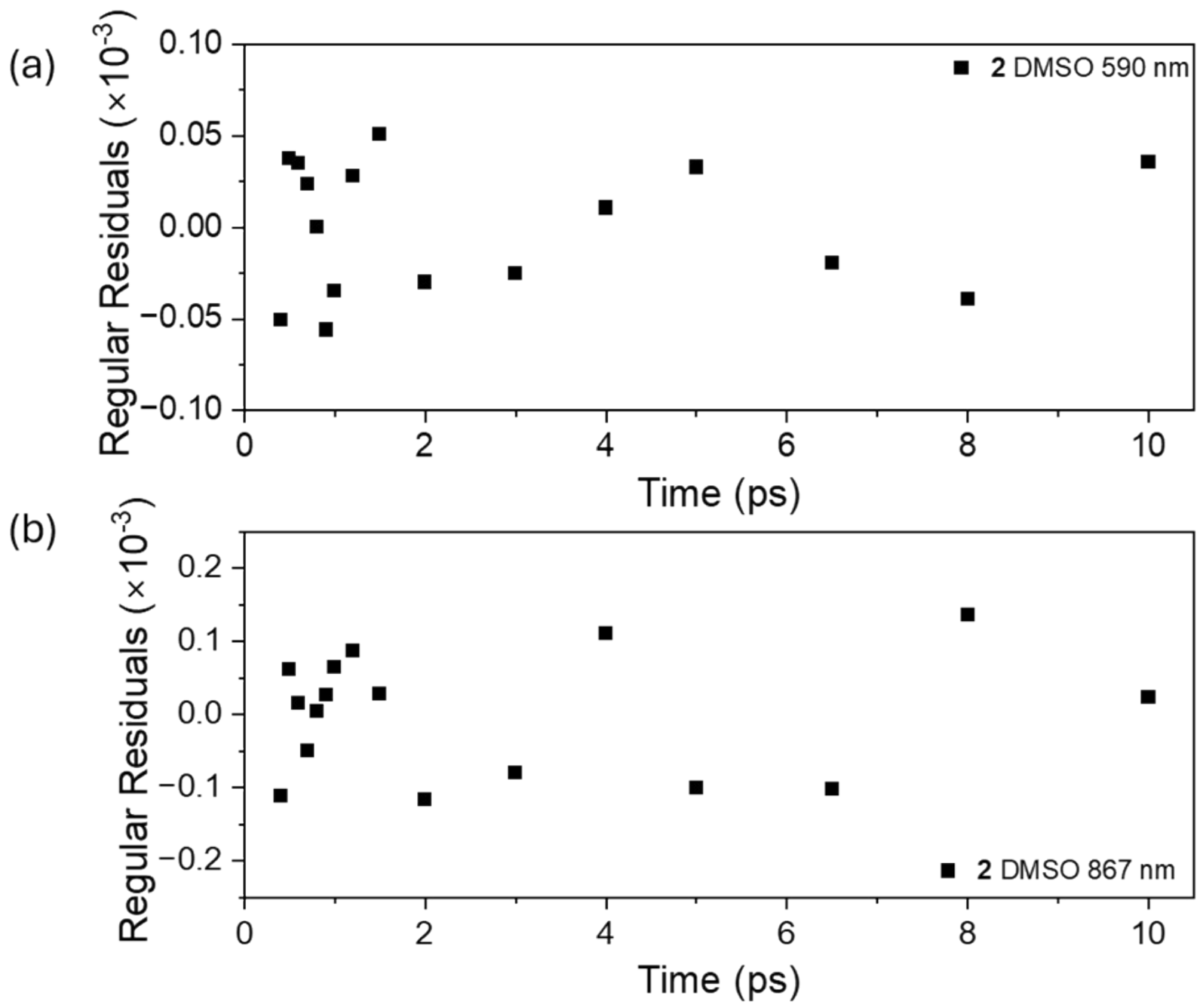

Equation	y = A1*exp(-x/t1) + y0
y0	0.0053 ± 2.36138E-5
A1	-0.00216 ± 4.00304E-5
t1	1.74053 ± 0.08625
Reduced Chi-Sqr	1.49418E-9
Adj. R-Square	0.99628

Equation	y = A1*exp(-x/t1) + y0
y0	0.00137 ± 4.94027E-5
A1	0.00388 ± 1.06914E-4
t1	1.4845 ± 0.09408
Reduced Chi-Sqr	8.03569E-9
Adj. R-Square	0.99336

Figure S26. Residuals and fit details of Fig. 6c.

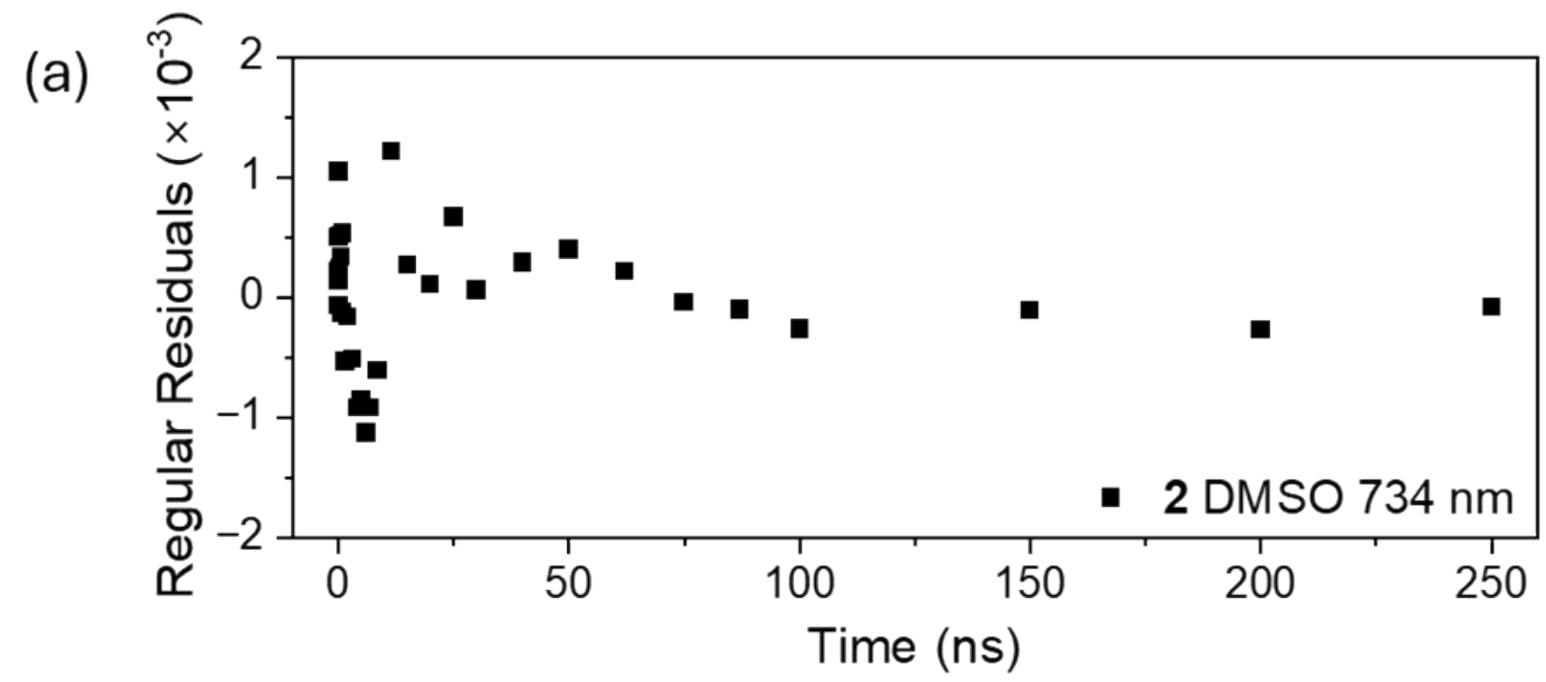

Equation	y = A1*exp(-x/t1) + y0
y0	-1.39004E-4 ± 2.37117E-4
A1	-0.03048 ± 2.66319E-4
t1	22.37581 ± 0.60585
Reduced Chi-Sqr	3.19183E-7
Adj. R-Square	0.99786

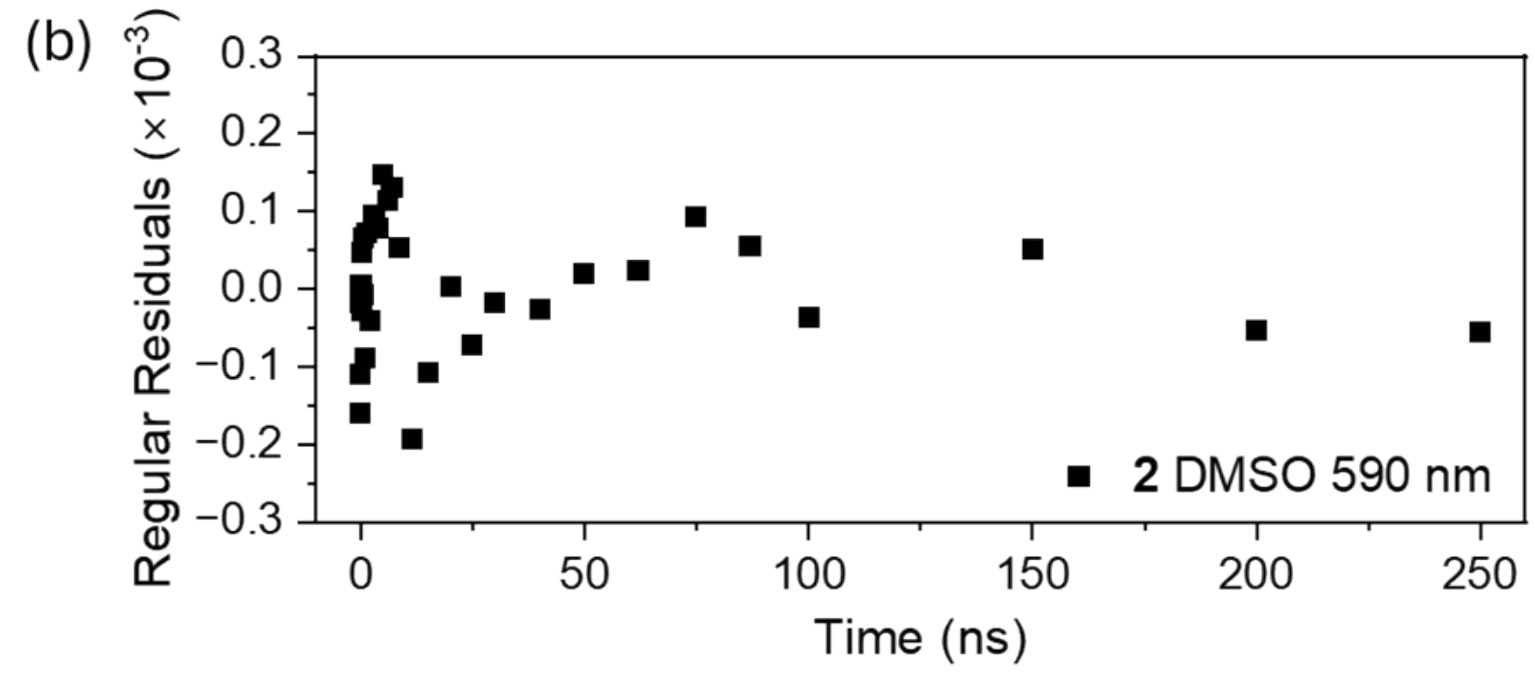

Equation	y = A1*exp(-x/t1) + y0
y0	6.7714E-5 ± 3.5861E-5
A1	0.00536 ± 4.01159E-5
t1	22.84409 ± 0.52912
Reduced Chi-Sqr	7.1712E-9
Adj. R-Square	0.99843

Figure S27. Residuals and fit details of Fig. 6d.

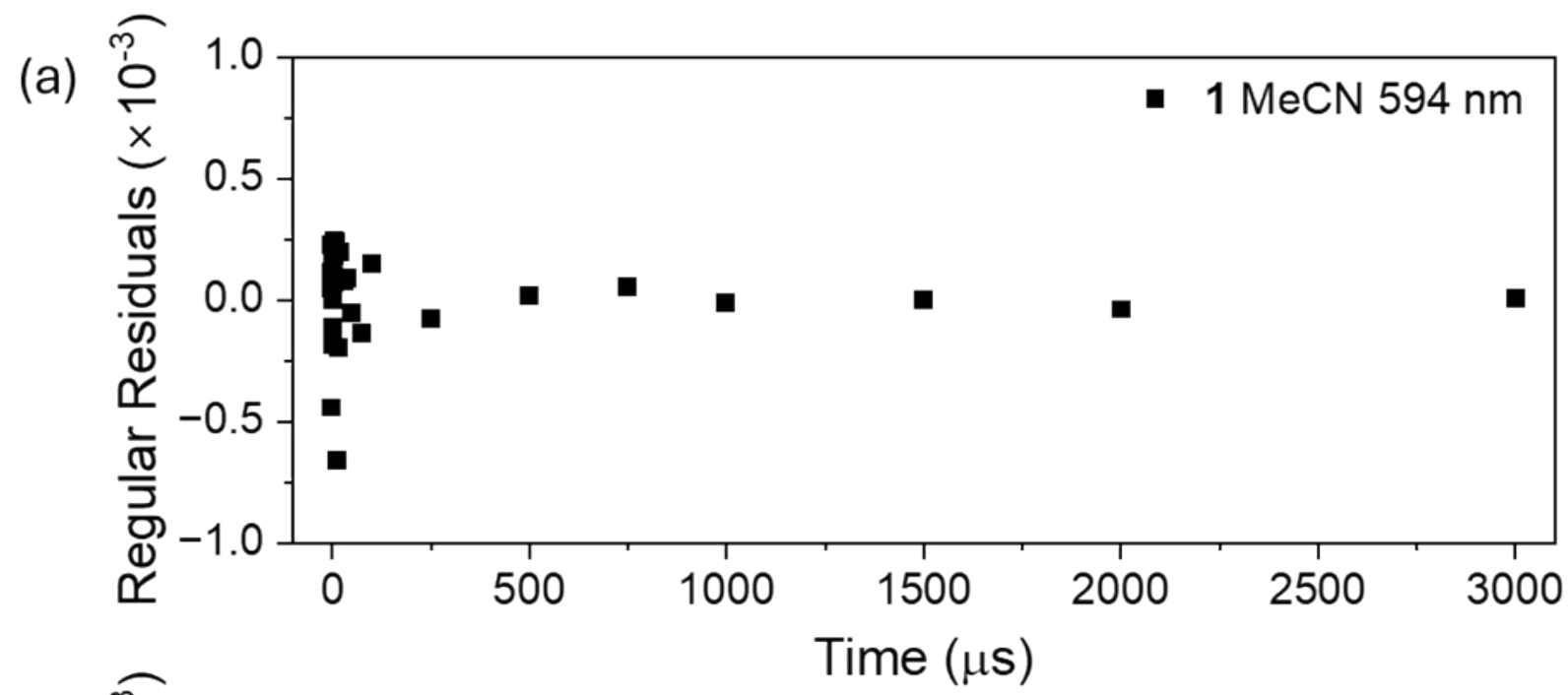

Equation	y = A1*exp(-x/t1) + A2*exp(-x/t2) + A3*exp(-x/t3) + y0
y0	1.91853E-4 ± 1.15494E-4
A1	0.00539 ± 2.83594E-4
t1	0.11371 ± 0.01207
A2	0.00322 ± 9.46054E-4
t2	43.18706 ± 13.88118
A3	0.00593 ± 9.34696E-4
t3	264.90096 ± 49.13763
Reduced Chi-S	4.44085E-8
Adj. R-Square	0.99716

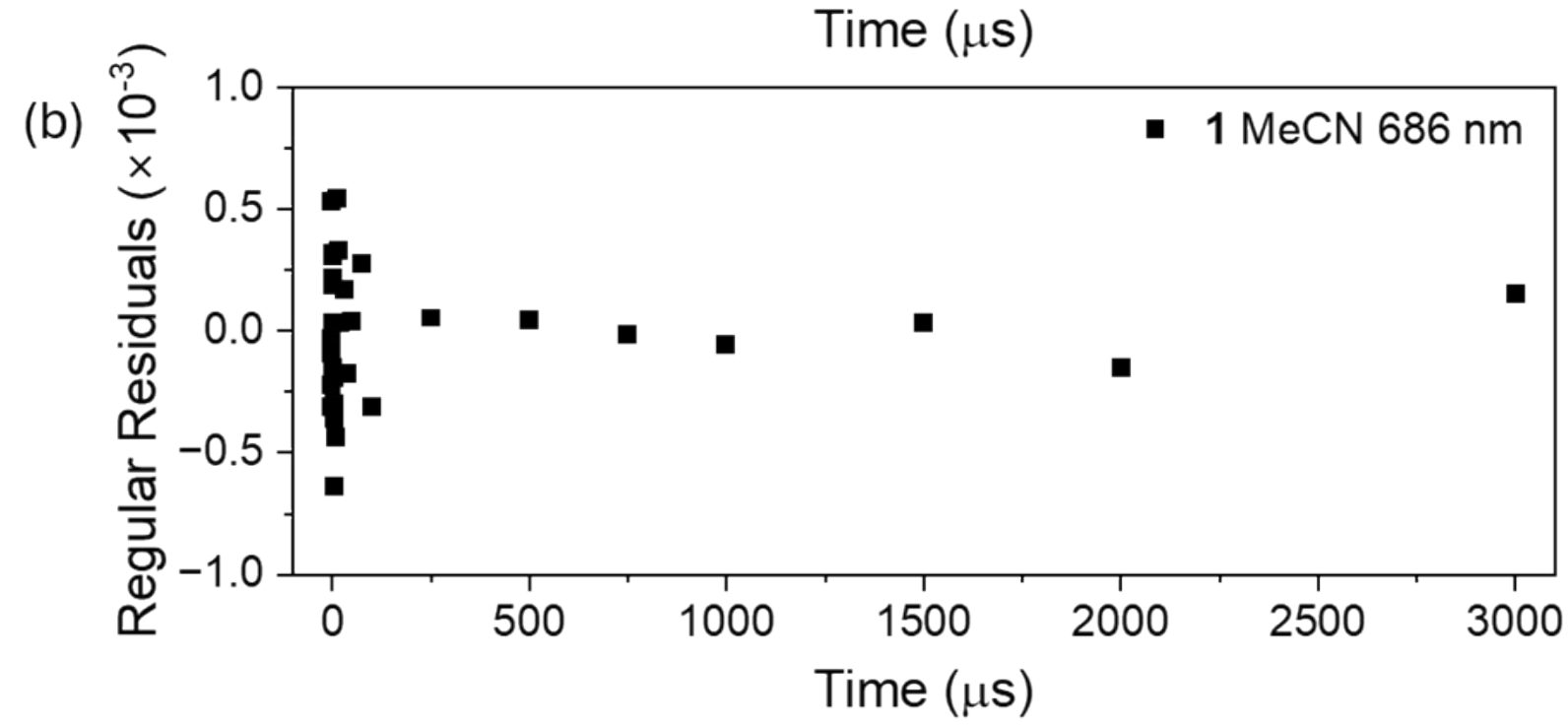

Equation	y = A1*exp(-x/t1) + A2*exp(-x/t2) + A3*exp(-x/t3) + y0
y0	-1.58592E-4 ± 1.98627E-4
A1	-0.01367 ± 3.96421E-4
t1	0.12066 ± 0.00722
A2	-0.00864 ± 9.86691E-4
t2	48.2977 ± 7.02944
A3	-0.00425 ± 9.55451E-4
t3	389.07926 ± 124.42646
Reduced Chi-S	9.33121E-8
Adj. R-Square	0.99782

Figure S28. Residuals and fit details of Fig. 10c.

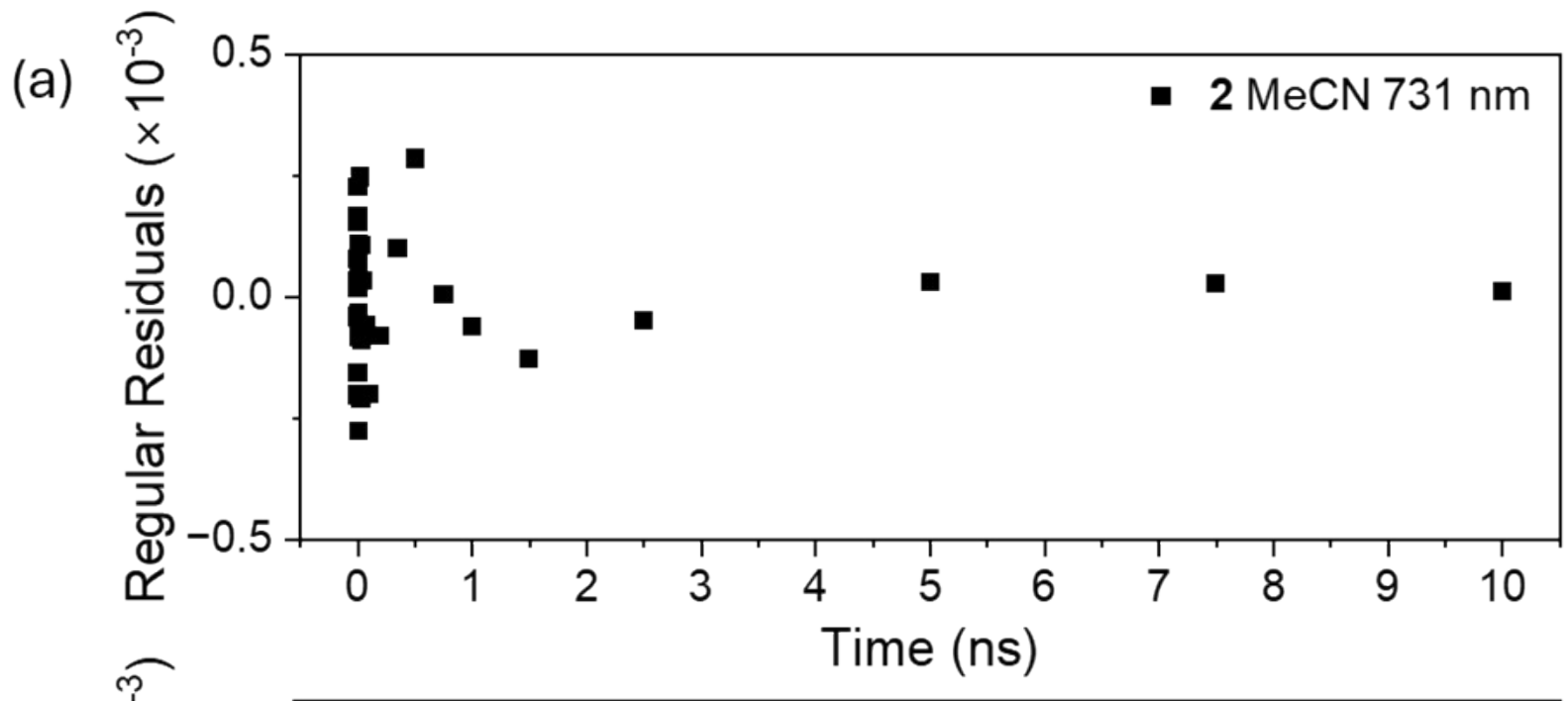

Equation	y = A1*exp(-x/t1) + A2*exp(-x/t2) + A3*exp(-x/t3) + y0
y0	-0.00879 ± 9.33015E-5
A1	-0.00446 ± 2.31801E-4
t1	1.20108 ± 0.12775
A2	-0.00339 ± 1.79761E-4
t2	41.96366 ± 5.18041
A3	-0.00577 ± 1.80347E-4
t3	1157.78703 ± 84.09316
Reduced Chi-S	2.4726E-8
Adj. R-Square	0.99831

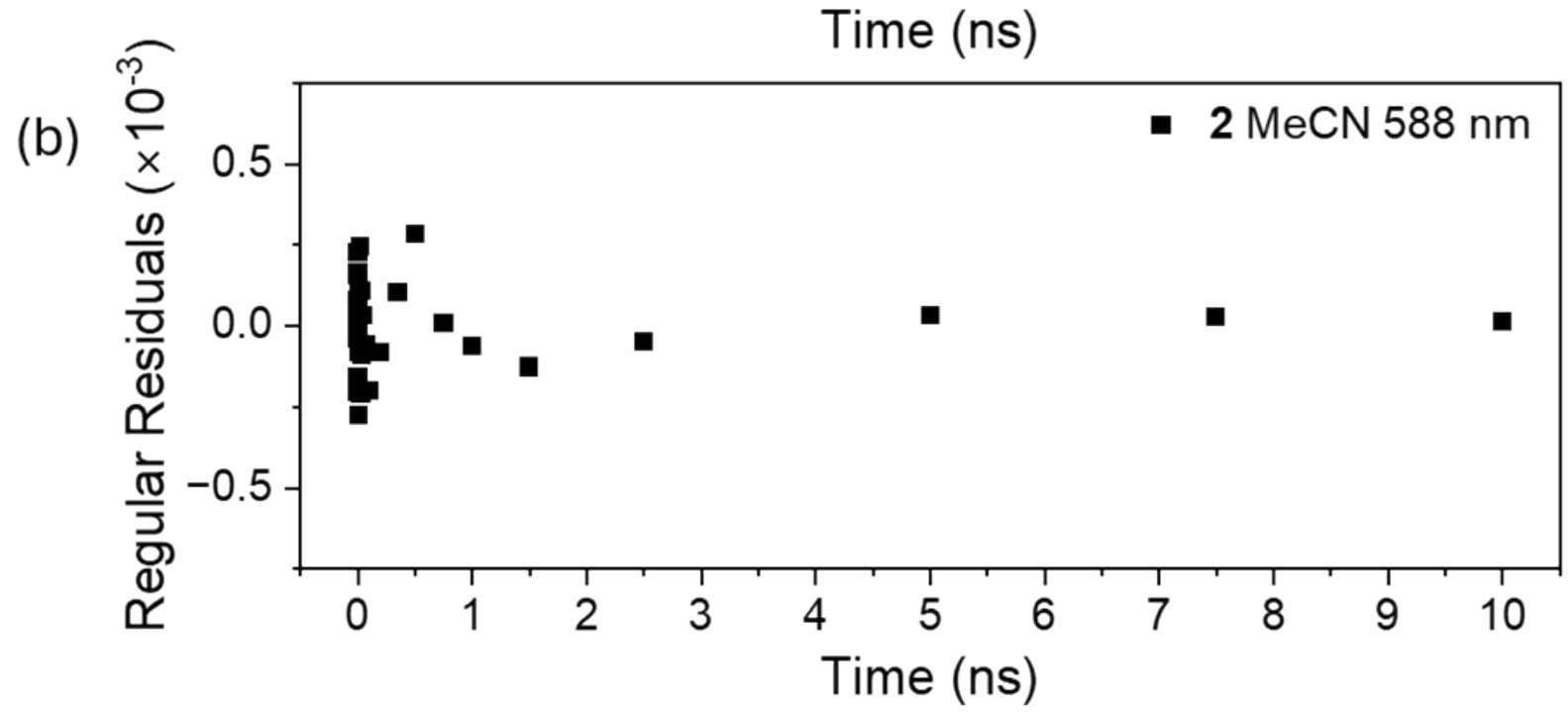

Equation	y = A1*exp(-x/t1) + A2*exp(-x/t2) + A3*exp(-x/t3) + y0
y0	0.003 ± 6.15414E-5
A1	0.00128 ± 6.30248E-5
t1	3.2494 ± 0.44223
A2	0.00114 ± 8.79051E-5
t2	97.68701 ± 18.51423
A3	0.00109 ± 7.95532E-5
t3	2322.82699 ± 539.76707
Reduced Chi-S	4.71919E-9
Adj. R-Square	0.99589

Figure S29. Residuals and fit details of Fig. 10d.

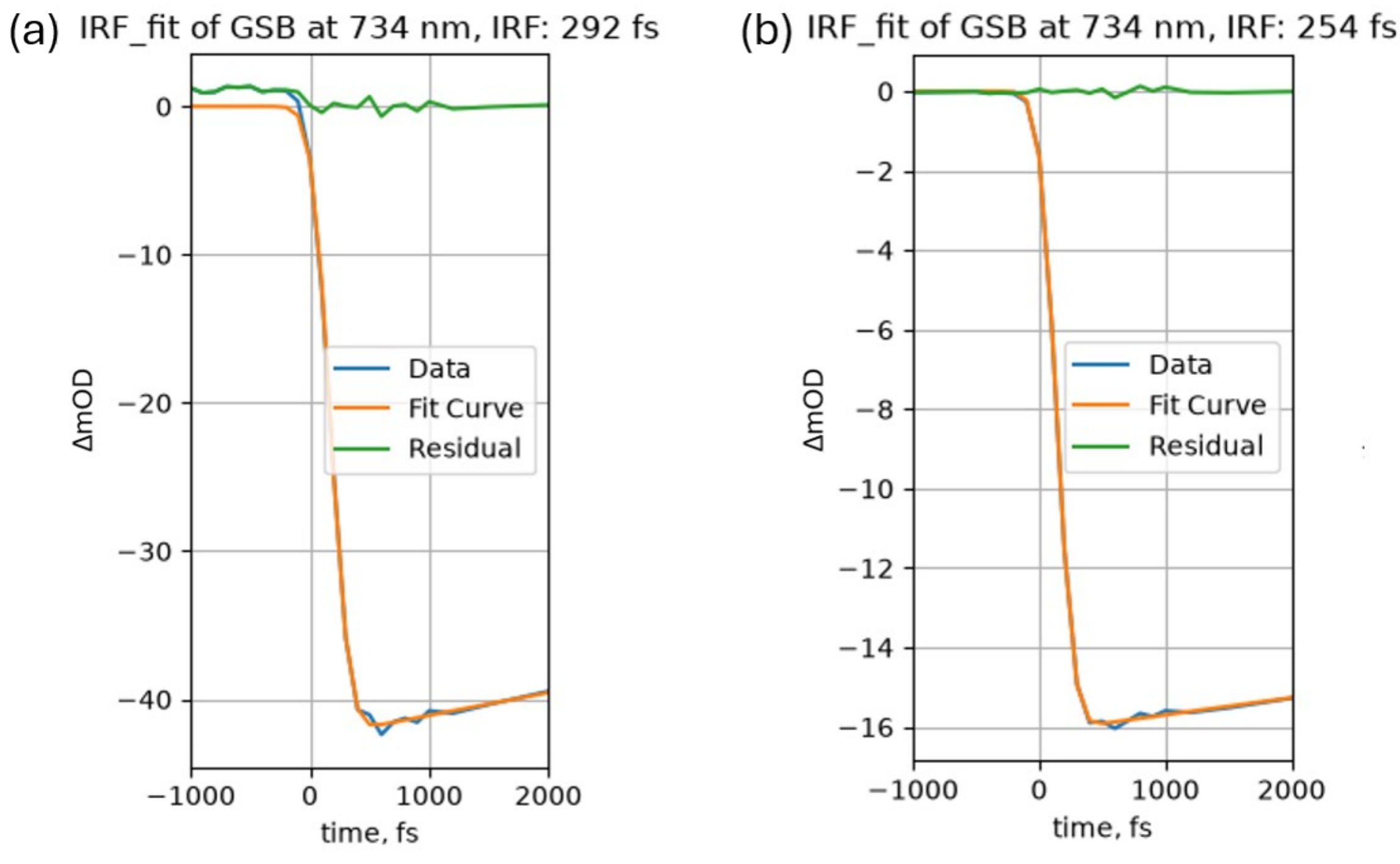

Figure S30. IRF fitting of GSB (734 nm) of (a) **1** in DMSO. (b) **1** in H_2O.

Table S1. Summary table of excited state dynamics of **1** and **2**.

	**Solvent**	τ_{IC} **(ps)**	τ_F **(ns)**[a] **[**Φ_F**]**	τ_{ISC} **(ps)**	τ_T **(µs)** [b]	Φ_T[c]
**1**	DMSO	-	1.9 [0.12]	-	3.0<br>196 (N_2)	0.57
**1**	DMSO-*d₆*	-	2.0	-	175 (N_2)	0.52
**1-*d₁₂***	DMSO	-	-	-	184 (N_2)	0.50
**2**	DMSO	-	-	1.7	0.0228	~1
**1**	H_2O	53	1.8 [0.0014]	-	-	0.067
**2**	H_2O	-	-	0.8	0.0140	~1
**1**	D_2O	76	2.5	-	0.088 + 3.9	0.066
**2**	D_2O	-	-	0.8	0.0197	~1
**1**	1:1 DMSO:D_2O	140	2.4	-	24 + 124 (N_2)	0.32
**2**	7:3 DMSO:D_2O	-	-	1.2	0.0281	~1
**1**	MeCN	2.4 + 121	1.6	-	0.048 + 0.389	0.34
**2**	MeCN	1.2–3.2<br>+ 42–98	-	-	0.0012–<br>0.0023	-
**1**	Water[d]<br>+ 3% pyridine	-	2.0<br>2.6 (D_2O)	-	1.9<br>40 (N_2)	0.35

[a] Measured via TCSPC. [b] Measured in air-saturated solution, except where noted as N_2 purged. [c] Φ_T approximated by % of ground state bleach remaining at 10 ns for **1**. [d] H_2O, except where specified.

References

[1] C. Rennie, E. Sitch, J. Hillis, R. Edkins, *ChemRxiv*. 2022, DOI: 10.26434/chemrxiv-2022-r25k8.

[2] E. R. Windle, C. C. Rennie, R. M. Edkins, S. J. Quinn, *Chem. Eur. J.*, 2025, **31**, e202403095

[3] G. M. Greetham, P. M. Donaldson, C. Nation, I. V. Sazanovich, I. P. Clark, D. J. Shaw, A. W. Parker M. Towrie, *Appl. Spectrosc.*, 2016, **70**, 645–653.

[4] I. H. M. van Stokkum, D. S. Larsen, R. van Grondelle, *Biochim. Biophys. Acta,* 2004, **1657**, 82-104.
